# A dimeric proteomimetic prevents SARS-CoV-2 infection by dimerizing the spike protein

**DOI:** 10.1038/s41589-022-01060-0

**Published:** 2022-06-02

**Authors:** Bhavesh Khatri, Ishika Pramanick, Sameer Kumar Malladi, Raju S. Rajmani, Sahil Kumar, Pritha Ghosh, Nayanika Sengupta, R. Rahisuddin, Narender Kumar, S. Kumaran, Rajesh P. Ringe, Raghavan Varadarajan, Somnath Dutta, Jayanta Chatterjee

**Affiliations:** 1 Molecular Biophysics Unit (MBU), Indian Institute of Science, Bangalore, India; 2 Virology Unit, Institute of Microbial Technology, Council of Scientific and Industrial Research (CSIR), Chandigarh, India; 3 Institute of Microbial Technology, Council of Scientific and Industrial Research (CSIR), Chandigarh, India

**Keywords:** Chemical tools, Protein design

## Abstract

Protein tertiary structure mimetics are valuable tools to target large protein–protein interaction interfaces. Here, we demonstrate a strategy for designing dimeric helix-hairpin motifs from a previously reported three-helix-bundle miniprotein that targets the receptor-binding domain (RBD) of severe acute respiratory syndrome-coronavirus-2 (SARS-CoV-2). Through truncation of the third helix and optimization of the interhelical loop residues of the miniprotein, we developed a thermostable dimeric helix-hairpin. The dimeric four-helix bundle competes with the human angiotensin-converting enzyme 2 (ACE2) in binding to RBD with 2:2 stoichiometry. Cryogenic-electron microscopy revealed the formation of dimeric spike ectodomain trimer by the four-helix bundle, where all the three RBDs from either spike protein are attached head-to-head in an open conformation, revealing a novel mechanism for virus neutralization. The proteomimetic protects hamsters from high dose viral challenge with replicative SARS-CoV-2 viruses, demonstrating the promise of this class of peptides that inhibit protein–protein interaction through target dimerization.

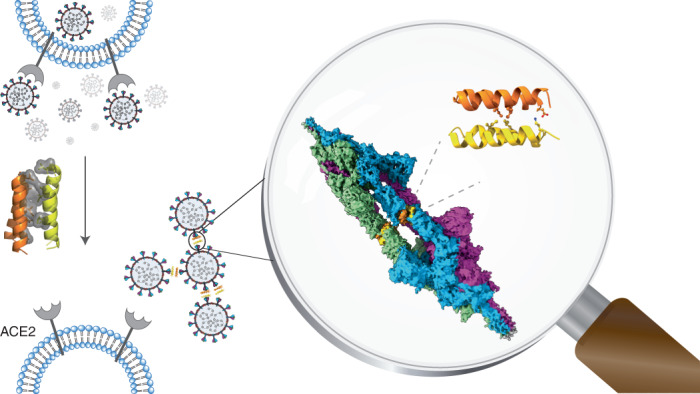

## Abstract (web summary)

A redesigned antiviral miniprotein based on a dimeric helix-hairpin motif binds and dimerizes the RBD of the SARS-CoV-2 spike protein and inhibits viral infection by inhibiting spike interaction with ACE2.

## Main

Conformationally stabilized protein secondary structure mimics of α-helices, β-sheets and loops have been extensively used as protein–protein interaction (PPI) inhibitors^1^. However, the efficient mimicry of relatively large and flat PPIs necessitates the use of more complex topologies that could be recapitulated by protein tertiary structure mimetics^2^. Helix-turn-helix motifs or helix-hairpins, wherein a pair of antiparallel amphipathic helices are connected by a covalent loop, are particularly well-suited for this purpose^3^. The design of synthetically amenable and conformationally stable monomeric helix-hairpin peptides was pioneered by Wells and coworkers, who showed the structural and functional mimicry of an IgG-binding three-helix-bundle Z-domain^4^. Their approach involved truncating the third helix that does not engage in target binding, incorporating charged residues at the exoface and stabilizing the helix-hairpin motif by a disulfide bridge. Subsequent work by Gellman and others successfully used this strategy to derive potent binders against human epidermal growth factor receptor 2 (HER2)^5^ and vascular endothelial growth factor (VEGF)^6^. In addition to disulfide bridges, monomeric helix-hairpin mimics targeting intracellular PPIs have been stabilized by substituting an interhelix salt bridge with a lactam bridge by Grossmann, Waldmann and coworkers, and covalent crosslinkers by Arora et al.^7,8^.

Dimeric helix-hairpin proteins such as Rop occur in nature^9^. However, seminal work of DeGrado^10^ and Baltzer^11^ toward the de novo design of four-helix-bundle proteins and helix-loop-helix motifs, respectively, established the principles for the design of dimeric helix-hairpins. To drive the formation of dimeric helix-hairpins, appropriately sculpting the heptad repeats in either helix with hydrophobic/hydrophilic residues is as important as the proper choice of loop residues^10,12^. However, unlike the β-turn in β-hairpins, which have been extensively studied and designed^13^, little attention has been paid to the design of interhelical loops. Thus, we sought to design dimeric helix-hairpin proteomimetics through loop optimization that could mimic the topology necessary for targeting complex PPIs and potentially induce dimerization of the target. The interaction between the receptor-binding domain (RBD) of severe acute respiratory syndrome-coronavirus-2 (SARS-CoV-2) and the human angiotensin-converting enzyme 2 (ACE2) served as a fertile ground for testing our hypothesis.

SARS-CoV-2, which emerged in late 2019, caused a global COVID-19 outbreak^14^ by infecting roughly 500 million humans and killing more than 6 million humans worldwide. SARS-CoV-2 infection is mediated by RBD of the spike glycoprotein on the viral surface through binding to the cell-surface receptor ACE2 (ref. ^15^). After the attachment of the virus to the ACE2 receptor, the spike protein is cleaved by the transmembrane serine protease 2 (TMPRSS2). This facilitates conformational transitions that induce membrane fusion and subsequent release of the viral RNA genome into the host cell cytoplasm^16^. While vaccinations against SARS-CoV-2 have been developed, the emergence of new strains of SARS-CoV-2 such as B.1.1.7 (alpha), B.1.351 (beta), B.1.1.28 (gamma), B.1.617.2 (delta), B.1.1.529 (omicron) decreases the protective ability of current vaccines, thus creating a huge challenge in controlling the COVID-19 pandemic^17^. The X-ray and cryogenic-electron microscopy (cryo-EM) structures of the SARS-CoV-2 spike protein bound to ACE2 revealed that the N-terminal helices of the ACE2 receptor mainly engage with the RBD of SARS-CoV-2 (ref. ^18^). Therefore, rational structure-guided design of α-helical peptides that mimic the ACE2 receptor interaction has been a sought-after strategy to develop pre-exposure prophylactics^19–23^. Nonetheless, most ACE2 derived single-helical peptide mimics are not potent enough to compete against the high-affinity SARS-CoV-2–ACE2 interaction (*K*_D_ of roughly 14 nM)^24^, and thus impractical for clinical development^21,25,26^. However, the recent de novo design approaches have led to the elucidation of ultra-stable and potent miniproteins that out-compete ACE2 binding^19,20^.

The computationally designed three-helix-bundle miniprotein LCB1 by Baker and coworkers^20^ served as a starting point for the design of dimeric helix-hairpin proteomimetic in the present study. By truncating LCB1 to two-thirds its size by removing one helix and subsequent optimization of the loop residues, we developed proteomimetics that show dimeric and higher oligomeric states. Irrespective of their oligomeric state, the proteomimetics bound to the RBD predominantly in a 2:2 stoichiometry and effectively compete out ACE2. The tight binding of the dimeric helix-hairpin (SIH-5) to the RBD led to the formation of a stable dimeric complex with the spike protein ectodomain, where all the three RBDs from either spike protein are firmly attached head-to-head in an open conformation, thus revealing a new mechanism for virus neutralization.

## Results

### Designing helix-hairpins to compete out ACE2 binding to RBD

**Fig. 1:**
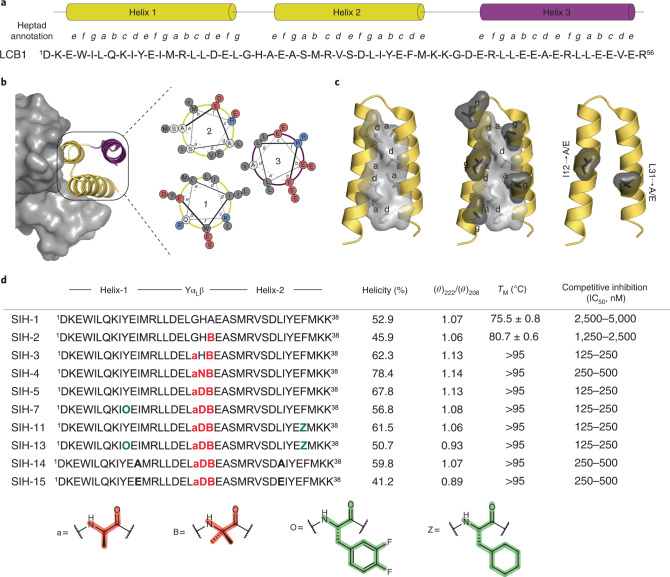
Rational design of dimeric helix-hairpin. **a**, Sequence and secondary structure map of LCB1. **b**, Cryo-EM structure of SARS-CoV-2 RBD in complex with LCB1 (7JZU). Helices 1 and 2 make direct contact with the RBD of SARS-CoV-2 and helix 3 (magenta) stabilizes LCB1 by packing against helices 1 and 2 (yellow). **c**, Removal of helix 3 exposes the hydrophobic residues at *g* positions in helices 1 and 2 (dark gray) and increases the width of the hydrophobic face. The hydrophobic residues (I12 and L31) that are substituted to obtain SIH-14 and SIH-15 are shown. The figure was generated using Pymol. **d**, Sequences, helicity, thermal stability and potency of the helix-hairpin peptides. The modified residues in the loop and helix are highlighted in red and green, respectively. Structure of unnatural amino acids: d-alanine (a), aminoisobutyric acid (B), 3,4-difluoro-l-phenylalanine (O) and l-cyclohexylalanine (Z). The midpoint of thermal transitions (*T*_M_) represents mean values ± s.d. derived from three independent experiments. (Helicity was measured at 20 °C in sodium-phosphate buffer, pH 7.4).

Toward the design of a helix-hairpin peptide, we analyzed the structure of the three-helix-bundle LCB1 (Fig. 1a,b and Supplementary Fig. 1). The in silico analysis of helix 3 lacking LCB1 revealed that the hydrophobic residues at the *a* and *d* positions of the heptad repeat from helix 1 and helix 2 engage in the classical knob-into-hole packing interaction^27^. This narrow hydrophobic core is flanked by the *e* and *g* positions and is mostly occupied by charged residues in dimeric coiled coils^28^. In the truncated LCB1, the *e* positions engage in binding to the RBD, whereas the *g* positions at the exoface are occupied by hydrophobic residues that increase the width of the narrow hydrophobic face (Fig. 1c). Although hydrophobic residues at the *e* and *g* positions were previously suggested to influence the oligomeric state of coiled coils^29^, Lu and coworkers demonstrated that altering the canonical 3–4 heptad repeat to a 3–3–1 hydrophobic repeat, the assembly of a stable antiparallel four-helix bundle could be achieved^30^.

**Extended Data Fig. 1:**
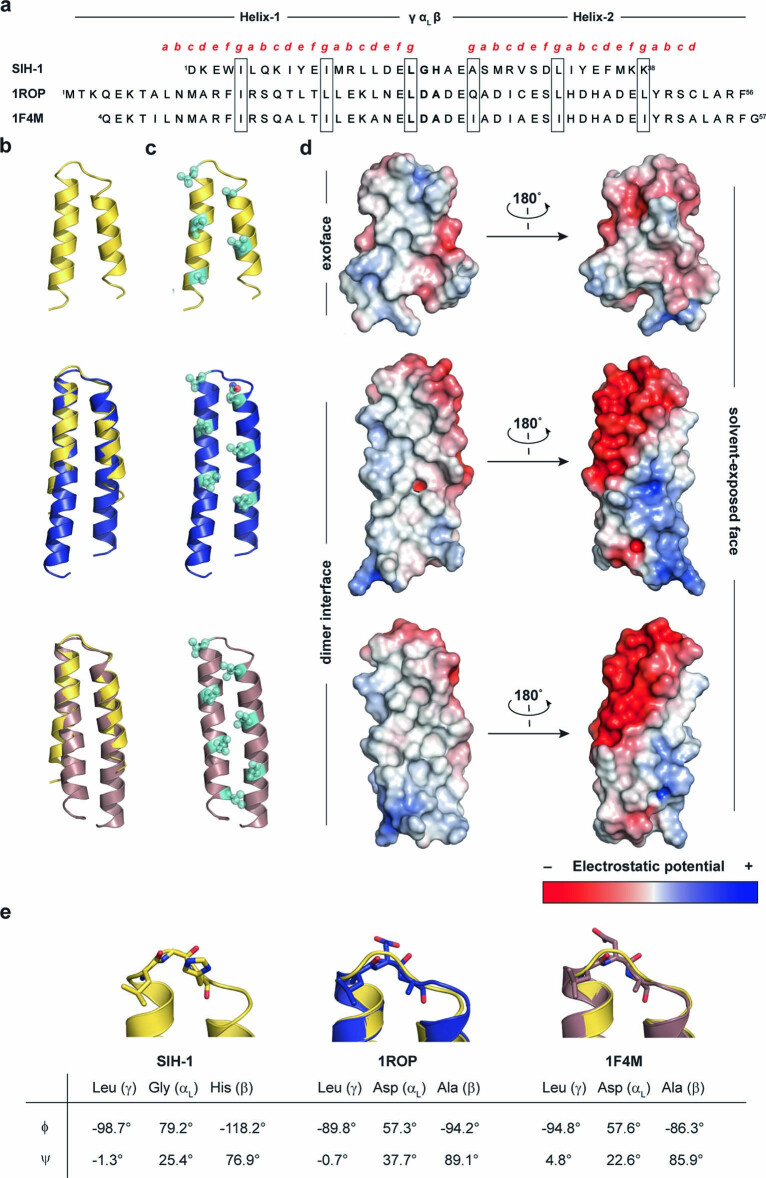
Comparison of SIH-1, 1ROP, and 1F4M. **a**, Sequences of SIH-1, naturally occurring homodimeric protein Rop (**1ROP**) and its engineered variant Ala_2_-Ile_2_–6 (**1F4M**). **b**, Structure of helix-3 truncated LCB1 (SIH-1) (yellow, PDB:7JZU) and its superimposition on Rop (blue, PDB:1ROP) and Ala_2_-Ile_2_–6 (copper brown, PDB:1F4M). **c**, The exoface of truncated LCB1 and the dimer interfaces of Rop and Ala_2_-Ile_2_–6, highlighting the residues at *g* sites in ball-and-stick representation. **d**, Electrostatic surface representation of SIH-1, Rop, and Ala_2_-Ile_2_–6. The surface is colored according to its electrostatic potential from red (negatively charged) to blue (positively charged). **e**, The residues forming the γ-α_L_-β loop in the helix-hairpins and their dihedral angles.

The structural comparison of helix 3 lacking LCB1, homodimeric Rop and a homodimeric Rop variant (Ala_2_-Ile_2_–6)^31^, shows stark similarity in the orientation of the side chains in the heptad repeat and the loop conformation (Extended Data Fig. 1). Here, the hydrophobic residues at the *g* site extend the narrow hydrophobic channel carved by the *a* and *d* residues resulting in an amphipathic hairpin. And the loop in all three adopts a comparable conformation as deduced from the *ϕ*, *ψ* dihedral angles. This led us to propose the crucial role of (1) loop residues to enforce a helix-hairpin through the optimal knob-into-hole packing interaction between the helices, and (2) the hydrophobic *g* residues in inducing dimerization.

**Fig. 2:**
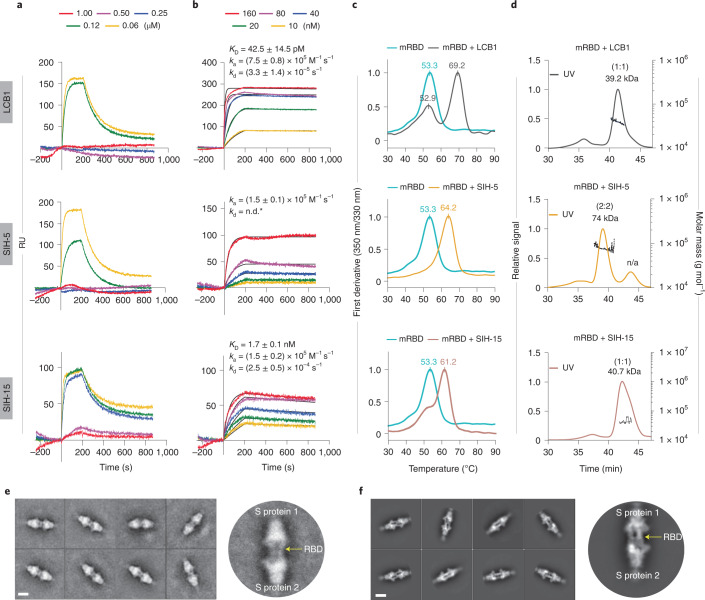
Biophysical characterization of synthetic helix-hairpin peptides. **a**, Competitive inhibition of SARS-CoV-2 RBD binding to immobilized ACE2 by the peptides determined by SPR. **b**, The direct binding affinity of peptides to SARS-CoV-2 RBD was assessed by SPR. Data are depicted as mean from two separate experiments ± s.d. n.d.*, No dissociation. **c**, The thermal stability of peptide–RBD complex was monitored by nanoDSF. Two independent measurements were done for each sample. **d**, The binding stoichiometry of the peptide–RBD complex determined through SEC–MALS. **e**, Negative staining, the data analysis was carried out in three sets with independent purification of Spike–SIH-5 complex. **f**, Cryo-EM reference-free 2D class averages of spike (S) protein in the presence of SIH-5. The experiment was repeated twice with similar results. The scale bar represents 10 nm. Source data

**Extended Data Fig. 2:**
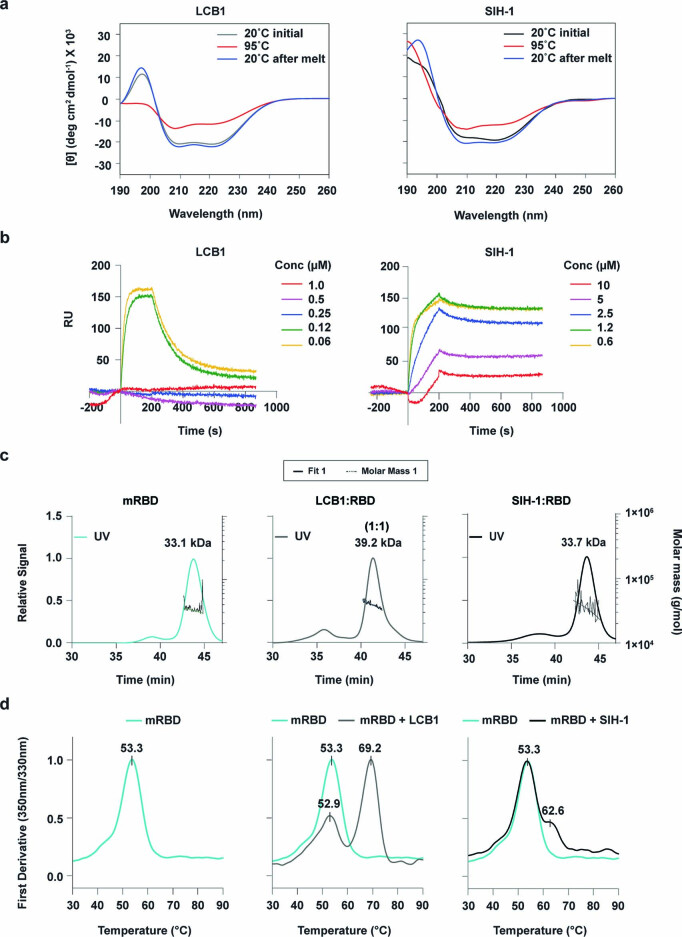
Truncation of helix 3 compromises the potency of LCB1. **a**, Circular dichroism (CD) spectra at different temperatures. **b**, Competitive inhibition of SARS-CoV-2 RBD binding to the immobilized ACE2 by the helix-hairpin peptides. **c**, Binding stoichiometry of peptide:RBD complex. **d**, Thermal stability of LCB1:RBD and SIH-1:RBD complexes. A subset of data from Fig. 2 is replicated for better comparison. Source data

To generate a dimeric helix-hairpin, we truncated the helix 3 of LCB1. SIH-1 showed comparable helicity to LCB1 and the ratio between 222 and 208 nm ellipticities, with a value >1 indicated the presence of coiled helices (Fig. 1d and Supplementary Fig. 2). SIH-1 was not thermostable with very low solubility in phosphate buffer (pH 7.4); however, it showed full reversibility after thermal denaturation (Extended Data Fig. 2). We next evaluated the potency of SIH-1 to compete with ACE2 in binding to the RBD. For this, we performed surface plasmon resonance (SPR) to monitor the binding of RBD onto immobilized ACE2 in the presence of increasing concentration of SIH-1 (ref. ^24^). LCB1 completely inhibits the ACE2 binding to RBD at 250 nM (Fig. 2a), whereas we observed a dose-dependent inhibition of ACE2 binding only at tenfold higher concentrations of SIH-1 (Extended Data Fig. 2). We speculated that the poor inhibitory activity of SIH-1 is owing to its ‘partially open’ hairpin conformation due to the suboptimal knob-into-hole interaction between the hydrophobic *a* and *d* sites.

To evaluate whether the native LCB1 sequence results in a dimeric SIH-1, we incubated equimolar amounts of SIH-1 and RBD and determined their binding stoichiometry through size-exclusion chromatography coupled with multiangle light scattering (SEC–MALS). We noted the absence of a SIH-1–RBD complex at the highest achievable concentration of SIH-1 (12 μM). We confirmed the observation through binding-induced thermal shift change of RBD using nanoscale differential scanning fluorimetry (nanoDSF), wherein we could detect a small population of SIH-1–RBD complex (Extended Data Fig. 2). Thus, this suggested that the native loop of SIH-1 is inefficient in inducing the hairpin dimerization, resulting in solvent exposure of the hydrophobic patch and, consequently, its poor solubility.

Next, we systematically substituted the loop residues connecting the two helices to enforce a compact helix-hairpin. We initially substituted Ala^22^ at the N terminus of helix 2 with the helix-promoting aminoisobutyric acid, which modestly increased the thermal stability of SIH-2 (Fig. 1d), although its solubility remained low (Supplementary Table 1). SIH-2 is a better competitor of ACE2 than SIH-1, however, still with low efficiency (Supplementary Fig. 4). Based on the dihedral angles (*ϕ*, *ψ*) of the residues -Leu^19^-Gly-His^21^- determined from the structure of LCB1, the loop can be categorized into the γ-α_L_-β conformation put forth by Efimov^3,32^. To introduce a conformationally constrained loop, we substituted the flexible Gly^20^ in SIH-2 with D-Ala to enforce a rigid left-handed α-helical (α_L_) conformation^33^. The sharp increase in helicity of SIH-3 (62.3%) against SIH-2 (45.9%) is indicative of the favorable influence of α_L_ conformation adopted by D-Ala on the helix-hairpin. The enhanced helicity leads to improved knob-into-hole interaction of the hydrophobic residues at the *a* and *d* sites from helix 1 and helix 2 to form a hydrophobic seam. This subsequently leads to dimerization of SIH-3 by forming a hydrophobic core, which is evident from the increased solubility, marked increase in thermal stability (*T*_M_), and its predominantly dimeric state (Supplementary Figs. 3 and 5).

To further improve the helicity of SIH-3, His^21^ adopting the β-conformation was substituted with Asn (SIH-4) and Asp (SIH-5) to introduce an N-cap interaction with the N terminus of helix 2 (ref. ^34^). The high helical content, thermostability and solubility of these loop-engineered variants result from a stable and compact helix-hairpin structure (Fig. 1d). Consequently, SIH-3, SIH-4 and SIH-5 efficiently out-compete ACE2 in a dose-dependent manner, with SIH-5 being the most potent (Fig. 2a and Supplementary Fig. 4).

To evaluate the oligomeric state of SIH-5, we performed sedimentation-velocity and sedimentation-equilibrium analytical ultracentrifugation, which indicated that SIH-5 predominantly exists in a dimeric state (Supplementary Figs. 5 and 6). The dimeric state of SIH-5 led us to assess its use as a scaffold for targeting SARS-CoV-2 with mutations in the RBD and other PPIs. Thus, the structural and functional tolerance of SIH-5 on amino acid substitutions was determined. We incorporated isosteric unnatural amino acids at six different sites on SIH-5 that included residues at the RBD binding interface, buried residues between helices 1 and 2, and solvent-exposed residues (Supplementary Fig. 2).

### Binding of helix-hairpin to RBD increase its stability

All the tested substitutions retained the stability of the helix-hairpins and efficiently inhibited the RBD–ACE2 interaction (Supplementary Figs. 3 and 4). However, in particular, three analogs SIH-7 (Tyr^10^*3,4*difPhe), SIH-11 (Phe^35^Cha) and SIH-13 (Tyr^10^*3,4*difPhe;Phe^35^Cha), despite their occurrence in varying oligomeric states (Supplementary Fig. 5), were potent competitors of ACE2. The comparable potency of SIH-5, SIH-7, SIH-11 and SIH-13 to LCB1 in inhibiting RBD binding to ACE2, led us to evaluate their binding affinity to RBD by SPR^24^. All the peptides bound to RBD with binding affinities in the range of 0.9–3 nM (Supplementary Fig. 7). For SIH-5 we could not determine the binding affinity since the sensorgram at the highest concentration (160 nM) showed a rise in signal post 200 s dissociation time (Fig. 2b). Thus, we re-analyzed the data considering 200 s as the dissociation time, which returned a binding affinity of roughly 900 pM. LCB1 bound to RBD with a binding affinity of 42 pM (Fig. 2b). Thus, the helix-hairpins showed more than 25-fold loss in binding affinity to RBD relative to LCB1. However, the comparable efficacy of the peptides and LCB1 in preventing the binding of RBD to ACE2 in the competition assay (Fig. 1d) was perplexing. Thus, we monitored the binding-induced thermal shift change of RBD in the presence of inhibitors by nanoDSF (Fig. 2c)^24^, where the change in *T*_M_ reports on the tight binding and stability of the inhibitor–RBD complex. On binding, LCB1 increased the melting temperature of RBD by 16 °C, whereas the SIH peptides increased the melting temperature of RBD maximally by 11 °C, confirming their weaker affinity to RBD. Although LCB1 binds tightly to RBD, two distinct thermal unfolding events corresponding to the free RBD (52.9 °C) and LCB1–RBD complex (69.2 °C) were observed. Curiously, a single thermal unfolding corresponding to the SIH–RBD complex was observed for SIH-5 and SIH-13 (Supplementary Fig. 8), indicating the absence of free RBD under the experimental condition.

### Helix-hairpins induce dimerization of RBD

**Extended Data Fig. 3:**
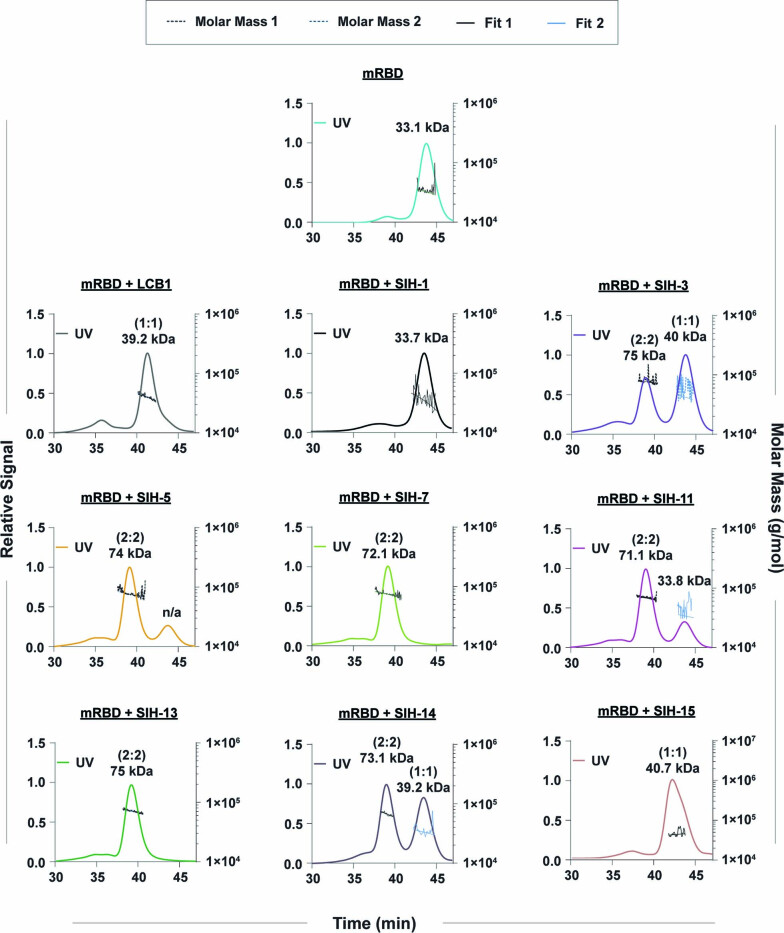
Binding stoichiometry of LCB1/SIH:RBD. The binding stoichiometry of LCB1/SIH peptides with SARS-CoV-2 RBD was determined by incubating equimolar concentrations of protein and ligand for 1 h at 4 °C followed by SEC-MALS analysis. A subset of data from Fig. 2 are replicated for better comparison and clarity. Source data

Next, we determined the binding stoichiometry of the SIH peptides to the RBD through SEC–MALS (Fig. 2d and Extended Data Fig. 3). The RBD eluted as a single peak with a molecular weight of roughly 33.1 kDa. Incubation of LCB1 (6.8 kDa) with RBD resulted in a single stable complex (39.2 kDa), indicating a binding stoichiometry of 1:1 (ref. ^20^). Incubation of SIH-5 and SIH-13 (4.6 kDa) with RBD resulted in a complex that eluted earlier than RBD with a molecular weight of roughly 75 kDa, suggesting a 2:2 binding stoichiometry. Thus, the formation of stable SIH-5–RBD and SIH-13–RBD dimer results in a single thermal unfolding as observed in nanoDSF (Supplementary Fig. 8). In contrast, SIH-3 (4.6 kDa) formed two distinct complexes with RBD, where the early eluting peak (75 kDa) correspond to the 2:2 complex and the later peak (40 kDa) correspond to a 1:1 (SIH-3–RBD) binding stoichiometry. Therefore, a stable dimeric helix-hairpin (SIH-5) can simultaneously engage two RBDs forming a stable complex. Despite the higher oligomeric state of SIH-13 (Supplementary Fig. 5), the formation of a 2:2 complex in the presence of RBD suggests a strong dimeric interface in SIH-13, but a relatively weak dimer–dimer association in the oligomeric state, which is disrupted on binding to the RBD. A similar phenomenon is observed for SIH-7 and SIH-11 (Supplementary Fig. 5 and Extended Data Fig. 3).

### Cryo-EM confirms the dimerization of spike protein by SIH-5

**Fig. 3:**
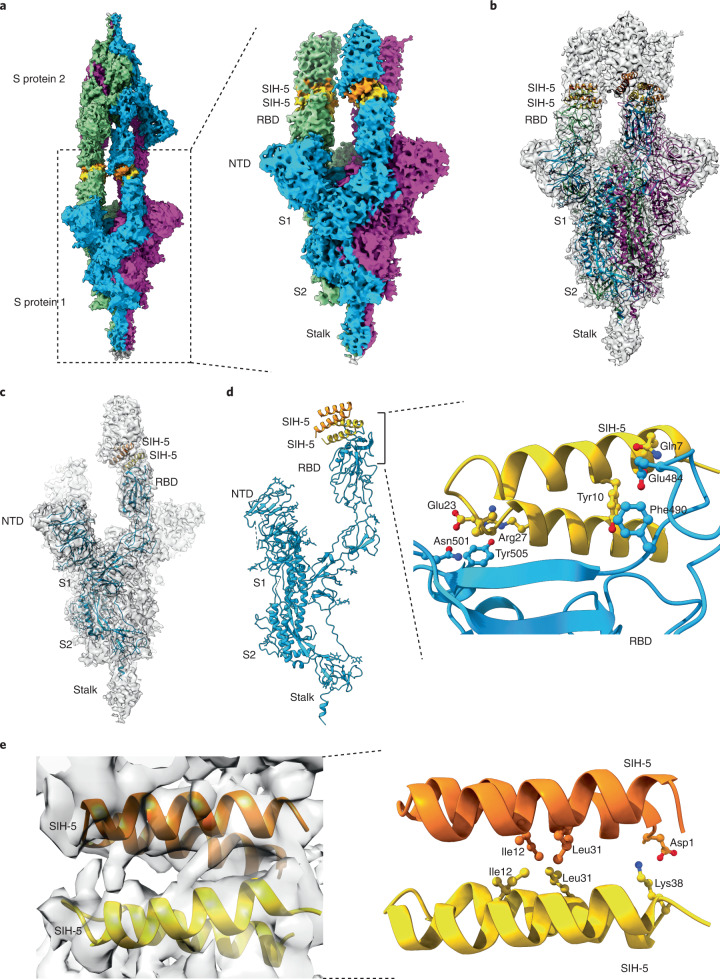
Synthetic helix-hairpin peptides induce dimerization of spike protein. **a**, Solid representation of cryo-EM 3D model of 3-RBD up dimeric spike protein in the presence of SIH-5. The three protomers of spike protein are colored in blue, green and purple, respectively. The SIH-5 protomers are colored in yellow and orange. The right panel shows an enlarged view of cryo-EM 3D model of a single 3-RBD up spike protein along with the dimeric SIH-5. NTD, N-terminal domain. **b**, Transparent representation of cryo-EM 3D model fitted with the calculated atomic model. **c**, Transparent representation of a single protomer of spike protein with dimeric SIH-5 fitted with the calculated atomic model. The spike protein protomer is colored in blue and SIH-5 dimer is colored in yellow and orange, respectively. **d**, The left panel shows the single protomer of spike protein with dimeric SIH-5 of the calculated atomic model. The right panel shows the enlarged view of spike protein RBD region (blue) bound to dimeric SIH-5, a single protomer is shown (yellow). The interacting residues are highlighted as ball-and-sticks. **e**, Transparent representation of dimeric SIH-5. The key residues for dimerization are highlighted as ball-and-sticks.

**Extended Data Fig. 4:**
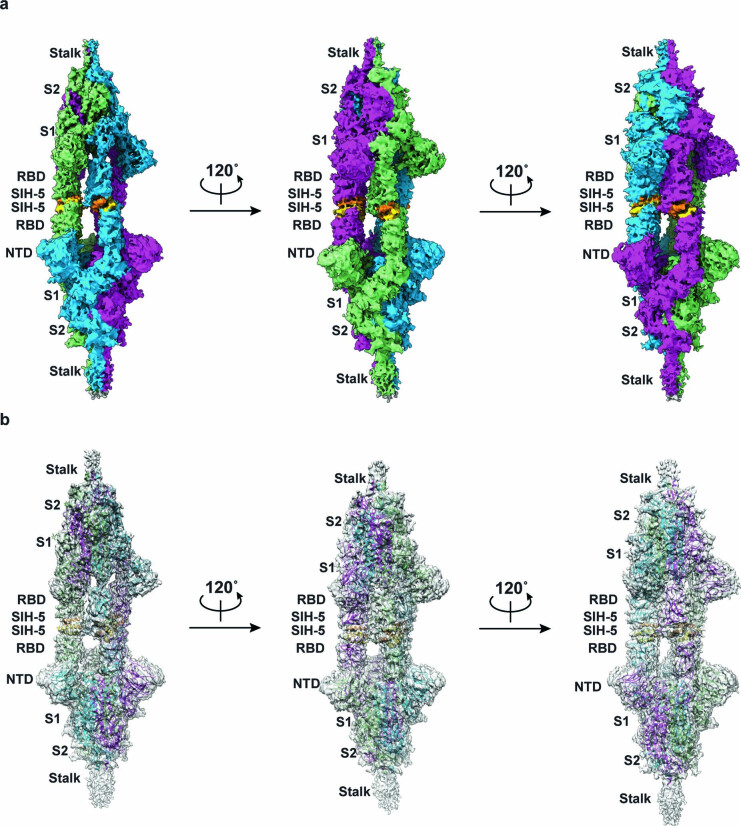
Cryo-EM model of 3-RBD up spike dimer. **a**, Solid representation of 3-RBD up spike dimer in the presence of SIH-5 at different orientations. The three protomers of spike protein are colored blue, green, and purple. The dimeric peptide is colored yellow and orange. **b**, Transparent representation of 3-RBD up spike dimer in the presence of SIH-5 at different orientations. The atomic model of spike protein with SIH-5 peptide was fitted using rigid body fitting in chimera.

To investigate the structure of dimeric SIH-5 bound to RBD and its consequence on the spike protein, we resorted to electron microscopy at physiological pH where 68% of the spike protein are present in an 1-RBD up conformation^35^. Room temperature negative staining transmission electron microscopy (TEM) followed by reference-free two-dimensional (2D) class averages were performed to visualize the SIH-5–spike protein complex. Negative staining indicated the absence of aggregation or distortion of the spike protein in the presence of SIH-5 (Supplementary Fig. 9). Nonetheless, the TEM images showed the occurrence of elongated spike protein particles, and the 2D reference-free class averages confirmed the association of two spike proteins (Fig. 2e). To obtain finer detail of the dimeric complex, we visualized the entire assembly at cryogenic conditions using single-particle cryo-EM. The three-dimensional (3D) classification indicated that most particles adopt a dimeric state (Fig. 2f and Supplementary Figs. 10 and 11). The structure solved to a global 5.4 Å resolution at 0.143 Fourier shell correlation (Supplementary Fig. 12), showed a dimeric spike protein ectodomain trimer with all three RBDs from either spike protein oriented ‘up’ in an open conformation and attached head-to-head (Fig. 3a and Extended Data Fig. 4).

**Extended Data Fig. 5:**
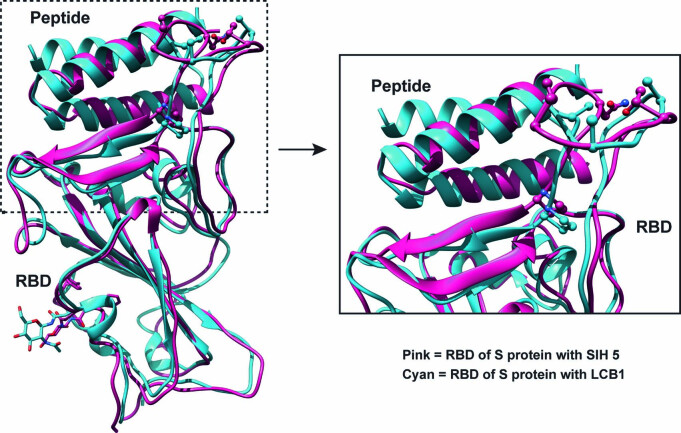
Comparison between the atomic models of LCB1:RBD (7JZU) and SIH-5:RBD (7X7N). Superimposition of atomic models of LCB1 bound RBD and SIH-5 bound RBD of spike (S) protein shows the difference in the RBD region. In the right panel, we noted the displacement of the Tyr473 ˗ Tyr489 loop towards SIH-5.

To identify the interacting residues between SIH-5 and spike protein, we improved the resolution of the single spike protein trimer bound to SIH-5, which was solved at a resolution range of 3.5–4.5 Å using a local resolution calculation and a global resolution of 4.47 Å at 0.143 Fourier shell correlation (Supplementary Fig. 13). The side chains of the S2 region in spike protein are visible in high-resolution maps, which allowed us to calculate the atomic model from the cryo-EM map (Supplementary Fig. 14). The atomic model successfully docked into the dimeric spike protein’s cryo-EM map, wherein the two spike protein models fit appropriately. The SIH-5 fit with high accuracy within the additional density present between the RBDs of either spike protein (Fig. 3b,c and Supplementary Video 1). The fitting of SIH-5 revealed an antiparallel four-helix bundle formed by the two helix-hairpin protomers, where both the loops are at one end and the N and C termini at the other (Fig. 3e). The total interacting surface area of SIH-5 with RBD is 944 Å^2^. However, at this resolution, we could identify only a few interacting partners (Fig. 3d). Of note, Gln^7^, Aib^22^ and Glu^23^ of SIH-5 interact via H-bonding interaction with Glu^484^ and Asn^501^ of the RBD that are known to undergo mutations^17^. The comparison of SIH-5–RBD spike protein complex with LCB1–RBD revealed that SIH-5 binds to RBD in the same fashion as LCB1, however, with a displacement in the loop (Tyr^473^–Tyr^489^), which moves toward the SIH-5 (Extended Data Fig. 5). Last, we noted that the dimer interface of SIH-5 is stabilized by a crucial electrostatic interaction between Asp^1^ and Lys^38^ from either protomer and several hydrophobic interactions (Fig. 3e).

### Homodimerization amplifies the potency of helix-hairpins

**Extended Data Fig. 6:**
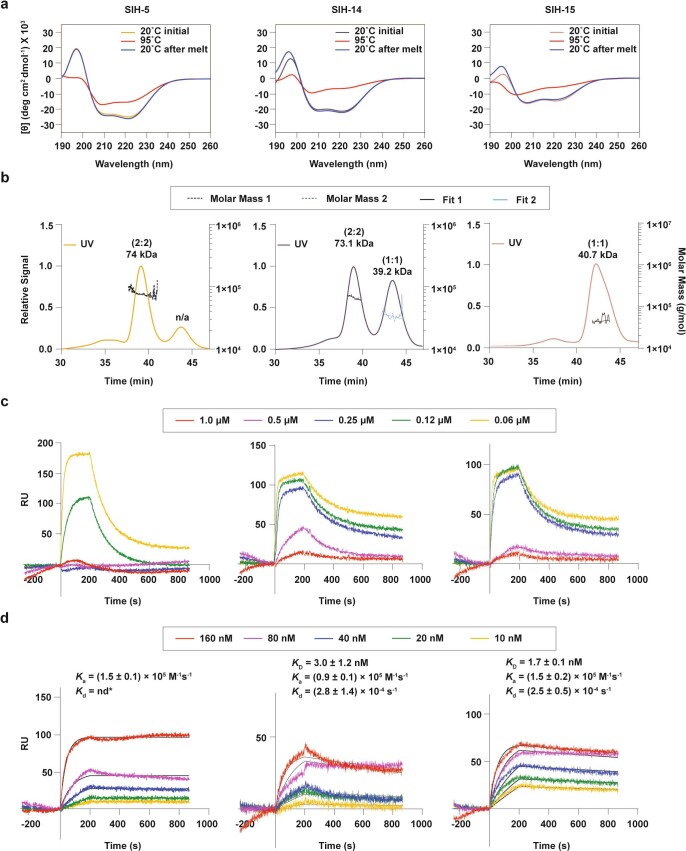
Hydrophobic residues at g sites induce homodimerization and amplify the potency of helix-hairpins. **a**, Circular dichroism (CD) spectra at different temperatures. **b**, Binding stoichiometry of peptide:RBD complex. **c**, Competitive inhibition of SARS-CoV-2 RBD binding to the immobilized ACE2 by the helix-hairpin peptides. **d**, The direct binding affinity of helix-hairpin peptides to SARS-CoV-2 RBD. A subset of data from Fig. 2 are replicated for better comparison. Source data

**Extended Data Fig. 7:**
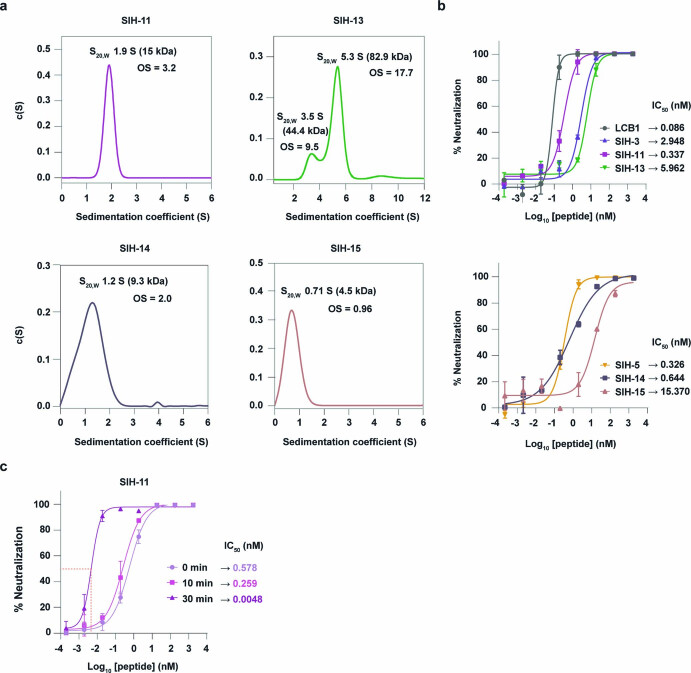
Monomeric and higher oligomeric states lead to inefficient virus neutralization. **a**, Sedimentation-velocity analytical ultracentrifugation data of SIH-11, SIH-13, SIH-14, and SIH-15. *OS – oligomeric state. **b**, Pseudovirus neutralization assay. Data represents the average and standard deviation of replicates (n = 2). The data for LCB1 and SIH-5 from Fig. 4 are replicated for better comparison. All pseudoviral assays were repeated thrice. **c**, Kinetics of the pseudoviral infection in the presence of SIH-11. Data represents the average and standard deviation of replicates (n = 2). All pseudoviral assays were repeated thrice. Source data

To evaluate the beneficial role of dimeric helix-hairpins in target engagement, we sought to develop monomeric helix-hairpins. Since the hydrophobic residues at the *g* sites are crucial for homodimerization, we substituted the Ile12 and Leu31 in SIH-5 with Ala and Glu to obtain SIH-14 and SIH-15, respectively (Figs. 1c and 3e). As the dimer interface transition from a hydrophobic to the polar surface, we observe a gradual reduction in helicity and *θ*_222_/*θ*_208_ of the helix-hairpins (Fig. 1d and Extended Data Fig. 6), resulting in monomeric SIH-15 (Extended Data Fig. 7). The partial destabilization of SIH-5 homodimer on alanine substitution results in 1:1 and 2:2 binding stoichiometries of SIH-14–RBD (Extended Data Fig. 6), while the complete destabilization by glutamate substitution results in a 1:1 complex of SIH-15–RBD (Fig. 2d). The loss of a stable homodimeric structure weakens the binding affinity of SIH-14 (3 nM) and SIH-15 (1.7 nM) to RBD against SIH-5 (0.9 nM). Despite the comparable binding affinities of SIH-15 and SIH-13 (1.6 nM) to RBD, we note a twofold lower potency of monomeric SIH-15 in inhibiting the RBD binding to ACE2 (Fig. 1d). Finally, on subjecting to thermal stress, the 1:1 SIH-15–RBD complex shows a distinct peak of RBD as opposed to the 2:2 SIH-5–RBD complex (Fig. 2c). Thus, the homodimerization of helix-hairpins results in bivalent inhibitors that form high-affinity and thermally stable 2:2 ligand–target complexes.

### Virus neutralization by helix-hairpins

**Fig. 4:**
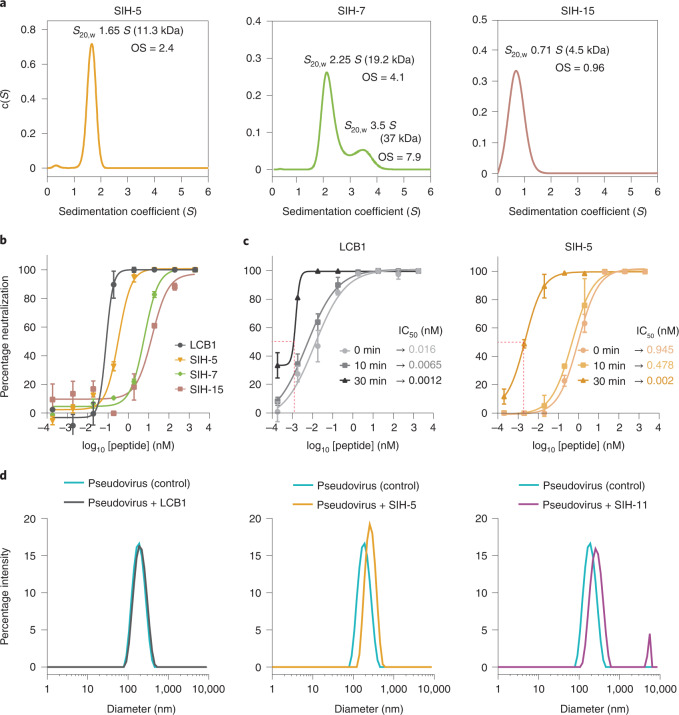
The effect of oligomeric state of helix-hairpin peptides on virus neutralization. **a**, Sedimentation-velocity analytical ultracentrifugation data of selected helix-hairpin peptides. OS, oligomeric state. **b**, Dose-dependent effect of the peptides in pseudovirus neutralization assay with hACE2-overexpressing HEK293T cells. The data represent the average and standard deviation of replicates (*n* = 2). All pseudoviral assays were repeated three times. **c**, Kinetics of the pseudoviral infection in the presence of LCB1 and SIH-5. SIH-5 displayed significant enhancement in virus neutralization from 0 to 30 min preincubation compared to LCB1. The data represent the average and standard deviation of replicates (*n* = 2). All pseudoviral assays were repeated three times. **d**, DLS intensity distribution for pseudovirus (control) and pseudovirus incubated with LCB1, SIH-5 and SIH-11. A single representative intensity distribution out of three independent DLS measurements for each condition is provided. Source data

We next used surrogate virus to evaluate the ability of the helix-hairpins to block the interaction of SARS-CoV-2 spike with the cellular receptor ACE2 and thereby inhibit the viral entry (Fig. 4 and Extended Data Fig. 7). In the standard pseudovirus neutralization assay, SIH-5 and SIH-11 were very potent with half-maximum inhibitory concentration (IC_50_) of 326 and 337 pM, respectively, approaching the potency of LCB1 (86 pM). Although the SIH-5 variants with noncanonical substitution in the helix, SIH-7, SIH-11 and SIH-13 formed a stable 2:2 complex with RBD, none could outperform SIH-5 in neutralizing the virus. The oligomeric state of these helix-hairpins sheds light on their relative potency of pseudovirus neutralization (Fig. 4a and Extended Data Fig. 7). The comparable binding affinities to RBD and the oligomeric state of SIH-5 (*K*_D_ 0.9 nM, oligomeric state 2.4) and SIH-11 (*K*_D_ 1.3 nM, oligomeric state 3.2) result in their efficient neutralization potency. Whereas two distinct and large oligomeric states in SIH-7 (*K*_D_ 2.9 nM, oligomeric state 4.1 and 7.9) and SIH-13 (*K*_D_ 1.6 nM, oligomeric state 9.5 and 17.7) lead to inefficient virus neutralization with IC_50_ of 5.8 and 5.96 nM, respectively, thus, highlighting the necessity of controlling the oligomeric state of SIH peptides to achieve a potent entry inhibition.

Concurrently, despite the stronger binding affinity of monomeric SIH-15 than the dimeric SIH-14 to RBD, roughly 20- and 45-fold lower neutralization potency of SIH-15 (IC_50_ 15.37 nM) against SIH-14 (IC_50_ 644 pM) and SIH-5 (IC_50_ 326 pM)﻿, respectively, emphasize the contribution of spike trimer dimerization in viral neutralization (Fig. 4b and Extended Data Fig. 7).

The spike protein is in the prefusion state on the surface of free virions. It undergoes molecular breathing^36^ that exposes the RBDs over time to enable interaction with ACE2. To assess the differential mode of action of LCB1 and the hairpin peptides, we preincubated the virus with the most potent peptides for different time points and determined the inhibition of viral entry (Fig. 4c). The entry inhibition by SIH-5 and LCB1, where the compounds were not preincubated with the virus (0 min) or preincubated for 10 min before the infection, correlated well with their respective binding affinities to RBD. At these time points, LCB1 was roughly 60–75-fold more potent than SIH-5 in neutralizing the virus. However, SIH-5 was equipotent to LCB1 in neutralizing the virus when preincubated for 30 min. An identical trend in neutralizing the virus was observed for SIH-11 (Extended Data Fig. 7). The significant enhancement (>450-fold) in the neutralization potency of SIH-5 at 30 against 0 min, as opposed to LCB1 (>10-fold), is indicative of the large structural reorganization for the dimerization of the spike proteins on the surface of virions by SIH-5. Since LCB1 neutralizes the virus through binding to the RBD without inducing spike dimerization, it shows more rapid neutralization kinetics than SIH-5.

The dimerization of the spike protein on the surface of virions would result in higher-order complexes/viral aggregates in the solution. Therefore, we incubated SIH-5 and SIH-11 with the pseudovirus and determined the size of viral particles by dynamic light scattering (DLS) (Fig. 4d). Since LCB1 binds to the spike protein in 1:1 stoichiometry, it served as a control to distinguish the mode of action of dimeric helix-hairpins. The viral particles had an average diameter of 155.5 ± 0.5 nm, agreeing well with the previous reports^37^. On incubating with LCB1, we observed a modest increase in the diameter of the viral particles. However, a stark increase in the size of viral particles, suggestive of aggregation^37^ was observed with SIH-5 and SIH-11, wherein SIH-11 (Supplementary Table 3) formed a discrete higher-order population. Furthermore, the sharp peak shape of the SIH-5 and SIH-11 treated pseudovirus, as opposed to a broad distribution, suggests a stoichiometric basis for viral aggregation.

### Prophylactic activity of SIH-5 in hamsters

**Fig. 5:**
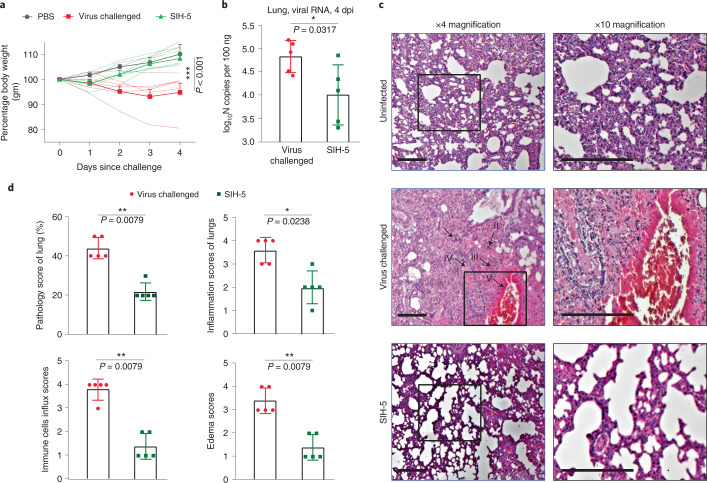
SIH-5 prophylaxis protects against SARS-CoV-2 infection. **a**, Body weight measurements through day 4 in three groups of Syrian hamsters: (1) control-PBS treated (*n* = 3), (2) challenged with 5 × 10^6^ PFU dose of SARS-CoV-2 (*n* = 5) and (3) SIH-5 treated (day 0 at −8 h) and challenged with 5 × 10^6^ PFU dose of SARS-CoV-2 (*n* = 5). The thick line represents average values, and the error bars indicate the standard deviation. Statistical significance was measured by a two-way analysis of variance test. ***P < 0.001. **b**, Viral load in the lungs of the hamsters at 4 days postinfection. Error bars indicate the standard deviation. Statistical significance was measured by a two-tailed Mann–Whitney test, *P* = 0.0317 (*P < 0.05, **P < 0.01). **c**, Representative histopathology of lung sections at 4 days postinfection in three groups; *n* = 3 for group (1), *n* = 5 for groups (2) and (3). In the middle panel (virus-challenged animals), the congestion of parenchyma and bronchopneumonia (I), alveolar edema and leukocytic alveolitis (II), blood hemorrhage (III), necrosis in bronchi filled blood and tissue exudates, acute bronchiolitis and infiltration of immune cells (IV), perivascular inflammation and vascular congestion (V) are highlighted. The scale bars represent 50 µm. **d**, Histopathological semi-quantitative scoring for lung pathology (*P* = 0.0079), inflammation (*P* = 0.0238), immune cells influx (*P* = 0.0079) and edema (*P* = 0.0079). Error bars indicate the standard deviation. Statistical significance was measured by a two-tailed Mann–Whitney test. *P < 0.05, **P < 0.01. Source data

Encouraged by the potency of SIH-5 and SIH-11 in neutralizing the virus, we evaluated the cytotoxicity of these peptides on Vero-E6 cells (Supplementary Fig. 16). At the highest concentration of the peptides tested (20 μM), SIH-5 and SIH-11 showed >75% cell viability at 48 h. A selectivity index (calculated by dividing the 50% cytotoxic concentration with IC_50_) of >50,000 was observed for both the peptides, suggesting their potential as antivirals. This led us to evaluate SIH-5 in preventing SARS-CoV-2 infection in vivo. We used the well-established Syrian hamster model^38,39^ and performed a single-dose prophylactic experiment. An intranasal dose of 2.5 mg kg^−1^ of SIH-5 was administered to the hamsters 8 h before the viral challenge with 5 × 10^6^ plaque-forming units (PFU) of SARS-CoV-2 US strain (USA-WA1/2020) (Supplementary Fig. 17). SIH-5 was efficient in preventing weight loss in hamsters from the second day postinfection (Fig. 5a) and accompanied a significantly reduced viral load in the lungs 4 days postinfection (Fig. 5b). We also monitored the clinical signs of disease progression in the three groups (Supplementary Fig. 18 and Table 4). Histopathology of the lung tissues at 4 days postinfection showed that the virus-challenged hamsters had a larger percentage area of lungs affected than the prophylactic and the unchallenged groups (Fig. 5c). The virus-challenged group showed moderate to severe pulmonary inflammation, bronchitis, bronchial and alveolar necrosis, and infiltration of lung tissues by immune cells (neutrophils, macrophages and lymphocytes). Although these phenotypes were observed in the prophylactic group, there was a significant reduction in severity and incidence (Fig. 5d).

### Loop and *g* site optimization results in hyperstable hairpin

**Fig. 6:**
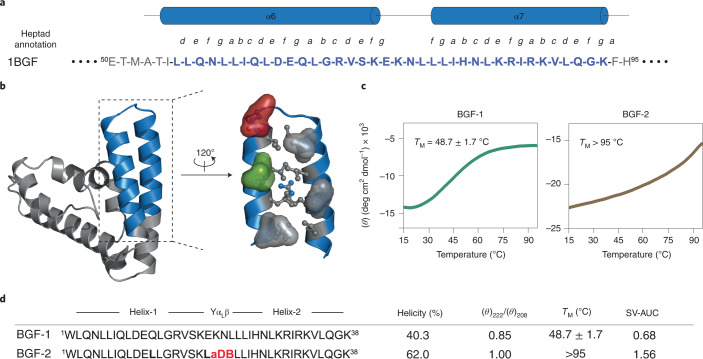
General strategy for stabilizing helix-hairpin peptides. **a**, Sequence and secondary structure map of an antiparallel coiled-coil region of the N-terminal domain of the transcription factor STAT-4. **b**, The X-ray crystal structure of the N-terminal domain of STAT-4 (PDB 1BGF). The antiparallel coiled-coil region chosen for truncation is highlighted in blue. The residues at *a* and *d* positions are shown in ball-and-stick representation. The surface of the residues at *g* positions are colored by residue: Glu is red, Gln is green, hydrophobic residues Leu and Ile are gray. **c**, Circular dichroism thermal unfolding transitions monitored at 222 nm. Data were fit to a two-state unfolding model to obtain the melting temperature (*T*_M_). The *T*_M_ represents mean values ± s.d. derived from three independent experiments. **d**, Sequences, helicity, thermal stability and oligomeric state of the BGF variants. The modified residues in the loop are highlighted in red and the modification at *g* sites are highlighted in bold. Leu1 to Trp1 is substituted to allow for absorbance measurement at 280 nm wavelength. Source data

To demonstrate the broad applicability of the design strategy to develop thermostable dimeric helix-hairpins, we chose an antiparallel coiled coil formed by helices α6 and α7 from the N-terminal domain of the transcription factor STAT-4 (signal transducers and activators of transcription), which mediate several PPIs^40^. Because our design allowed for the stabilization of helix-hairpins with less than three heptad repeats in each helix, we truncated STAT-4 to an identical length as SIH-1 (Fig. 6a,b). The 38-residue wild-type sequence (BGF-1) showed a modest helical signature and low thermal stability (*T*_M_ = 48.7 °C) (Fig. 6c and Supplementary Fig. 19). However, by introducing -D-Ala-Asp-Aib- at the interhelical loop and substituting the polar Gln^12^ and Glu^19^ at the *g* sites with hydrophobic Leu, we obtained BGF-2 that showed a significantly enhanced helicity and *θ*_222_/*θ*_208_, indicative of dimerization (Fig. 6d and Supplementary Fig. 20). BGF-2 showed exceptional thermal stability (*T*_M_ > 95 °C), which presumably results from the formation of a hydrophobic core achieved through the loop and *g*-site engineering.

## Discussion

In the pursuit of stable proteomimetics to target PPIs, we explored dimeric helix-hairpin peptides that present two identical faces for interacting with the target of interest. The de novo designed miniprotein LCB1, which bears structural similarity to the Z-domain of staphylococcal protein A derived three-helix-bundle affibody molecules^41^, was used for the investigation. The three critical steps used in the design of dimeric helix-hairpins are (1) truncation of the third helix; (2) widening of the narrow hydrophobic channel carved by the *a* and *d* residues through incorporating hydrophobic residue at the *g* sites and (3) optimization of the turn residues to enforce a rigid conformation inducing the proximity of helices 1 and 2. The design of helix-hairpins with fewer than three heptad repeats is challenging. Our findings indicate that interhelical loop re-engineering coupled with *g* site substitution to obtain a 3–3–1 hydrophobic repeat in the helices results in stable dimeric helix-hairpins. We note that subtle alteration in the amphipathicity of the helix-hairpin on substitution with hydrophobic amino acids reorganizes the oligomeric state of the dimeric helix-hairpin. However, when subjected to target binding, the high affinity between the helix-hairpin and the target protein dissociates the transient oligomeric states of the ligand into a dimeric ligand–protein complex that is stable under thermal stress.

The inhibition of PPI through target dimerization^42^, allowed us to prevent the binding of SARS-CoV-2 onto cell-surface ACE2 by dimerizing the spike protein. The dimerization of the spike protein on the surface of virions leads to noncovalent crosslinking of the individual spike proteins and enhanced blocking of the RBD through avidity effects. This significantly contributes to virus neutralization as observed for dimeric SIH-5 against monomeric SIH-15. It is worth noting that the head-to-head dimerization of spike trimer has also been identified in the SARS-CoV-2 kappa variant of concern (B.1.617.1). However, unlike in the SIH-5 induced spike trimer dimerization that inhibits the viral entry into cells, the labile dimer in the kappa variant has been suggested to increase the viral fitness against neutralizing antibodies by masking the antibody-accessible region of RBD^43^. Thus, the spike trimer dimerization is a unique mechanism to tune the viral fitness^44^. Despite its weaker affinity to RBD, the new binding mode of SIH-5 allowed us to achieve comparable potency in neutralizing the virus as the high-affinity RBD binder LCB1. The in vivo efficacy of the dimeric helix-hairpin devoid of toxicity suggests the potential for their development as new therapeutics. We see two different routes for the use of this class of molecules, one in which SIH-5 is used as a scaffold, and by substituting the solvent-exposed residues engaged in RBD binding, specific interaction with the target of interest is achieved. While the other involves a stepwise optimization of the turn residues and *g* sites of the helix-hairpin extracted from the PPI interfaces^45^ or three-helix-bundle affibody molecules^4–6,41^ to develop dimeric helix-hairpins. The exceptional stability of these target–ligand–ligand–target complexes reminiscent of irreversible covalent inhibitors would encourage the exploration of this unique class of proteomimetics to target PPIs while achieving the favorable pharmacology displayed by targeted covalent inhibitors^46^.

## Methods

### Peptide synthesis and purification

Synthetic helix-hairpin peptides (SIH-1 to SIH-15, BGF-1 and BGF-2) were synthesized on Rink Amide AM resin (0.8 mM g^−1^) on a 150 mg scale using standard Fmoc-based strategy as discussed earlier^13^. The global deprotection of protected peptides was carried out with 95% TFA, 2.5% triisopropylsilane and 2.5% water for 2 h. Deprotected crude peptide was precipitated in chilled ether. The white solid was pelleted by centrifugation and dissolved in 25% ACN/H_2_O for purification. Purifications were performed using a semipreparative column (Phenomenex C18, 250 × 10 mm, 110 Å, 5 µm) at a flow rate of 4 ml min^−1^ by reversed-phase–high-performance liquid chromatography (RP–HPLC) using gradient 35–75% B solvent over 30 min (solvent A: 0.1% TFA in water, solvent B: 0.1% TFA in acetonitrile). The purity was checked using an analytical column (Phenomenex C18, 250 × 4.6 mm, 100 Å, 5 µm) at a flow rate of 1 ml min^−1^ by RP–HPLC and the identity was confirmed by electrospray ionization–mass spectrometry.

LCB1 was synthesized on 2Cl-TCP resin (1.3 mmol g^−1^) using standard Fmoc-based chemistry. The global deprotection of protected protein was carried out with 95% TFA, 2.5% triisopropylsilane and 2.5% water for 2 h. Deprotected crude protein was precipitated in chilled ether. The white solid was pelleted by centrifugation and dissolved in 6 M Guanidine HCl in phosphate buffer for purification. Protein was purified by RP–HPLC (solvent A 0.1% TFA in water, solvent B 0.1% TFA in acetonitrile, gradient 25–85% B solvent over 30 min). The purity was checked by RP–HPLC and the identity was confirmed by electrospray ionization–mass spectrometry.

### Solubility measurement

Solubility of SIH-1 to SIH-5 was determined in sodium-phosphate buffer (pH 7.4). Approximately 2 mg of each peptide was added to 500 µl of buffer in 1.5-ml Eppendorf tubes. The tubes were shaken at 25 °C for 2 h on a 900 r.p.m. vortex shaker. The samples were centrifuged at 12,000 r.p.m. for 15 min to remove the precipitate and the supernatant was analyzed by ultraviolet–visible light (UV–vis) spectrometry.

### Circular dichroism spectroscopy

Far-UV circular dichroism spectra of the peptides were recorded on a JASCO-715 spectropolarimeter using 0.1 cm path-length cuvette at 50 μM concentration (SIH-1 and SIH-2 were measured at 15 μM concentration) in 20 mM sodium-phosphate buffer (pH 7.4). Scans were carried out at 20 and 95 °C and again at 20 °C after fast refolding (10 min) over the range of 190–260 nm with 0.5-nm increments and a 2-nm bandwidth. Thermal unfolding experiments were monitored at 222 nm over a temperature range of 4–98 °C with 0.5 °C interval in 20 mM sodium-phosphate buffer (pH 7.4). The samples were equilibrated for 2 min at each temperature. Temperature-dependent circular dichroism data were fit to a two-state unfolding model to obtain the melting temperature (*T*_M_).

Molar extinction coefficients were calculated for the peptides based on the number of Trp and Tyr residues in their sequences. In the case of SIH-6 and SIH-8, the molar extinction coefficient for unnatural amino acids l-2-napthylalanine and l-1-napthylalanine were used 3,936 M^−1^ cm^−1^ as reported earlier^47^. Mean residue ellipticity ((*θ*) deg cm^2^ dmol^−1^) and percentage helicities were calculated using equations (1) and (2), respectively.

1 $$\left[ \theta \right] = \theta _{{{{\mathrm{observed}}}}}/(10 \times l \times c \times n)$$

where *θ*_observed_ is the ellipticity measured in millidegrees, *l* is the path length of the cuvette (cm), *c* is the concentration in moles per liter and *n* is the number of residues in the peptide.

2 $$\% {{{\mathrm{helicity}}}} = \left[ \theta \right]_{222}/\left[ \theta \right]_{{{{\mathrm{max}}}}}$$

where [*θ*]_max_ = (−44,000 + 250 *T*) (1 − *k*/*N*_p_); *T* is the temperature (°C), *N*_p_ is the number of peptide units and *k* = 3 (C-terminal amidated peptide) and 4 (unblocked peptide).

### Expression and purification of mammalian expressed RBD (mRBD) from Expi293F

Proteins were expressed and purified as described previously^24,39^. Transfections were performed according to the manufacturer’s guidelines (Gibco, Thermo Fisher). Briefly, cells were passaged 1 day before transfection. On the day of transfection, cells were diluted to 3.0 × 10^6^ cells per ml. Desired plasmids were complexed with ExpiFectamine293 and transiently transfected into Expi293F cells. Transfected cells were treated after 16 h with Enhancer 1 and Enhancer 2. Five days posttransfection, culture supernatant was collected, proteins were affinity purified by immobilized metal affinity chromatography using Ni Sepharose 6 Fast flow resin (GE Healthcare) as described previously^39^. Briefly, the supernatant was twofold diluted with 1× PBS (pH 7.4) bound to a column equilibrated with PBS (pH 7.4). A ten-column volume wash of 1× PBS (pH 7.4), supplemented with 25 mM imidazole was performed. Finally, the bound protein was eluted with a gradient of 200–500 mM imidazole in PBS (pH 7.4). The eluted fractions were pooled and dialyzed three times using a 3–5 kDa (molecular weight cutoff) dialysis membrane (40 mm flat width) (Spectrum Laboratories) against PBS (pH 7.4). Protein concentration was determined by absorbance (A_280_) using NanoDrop 2000c with the theoretical molar extinction coefficient calculated using the ProtParam tool (ExPASy).

### Tag removal

The His-tag was removed by subjecting proteins to digestion with PreScission HRV-3C protease (Protein, HRV-3C = 50:1) in PBS (pH 7.4) buffer and incubating at 4 °C for 16 h. The untagged protein was separated from the remaining tag protein and protease by immobilized metal affinity chromatography using Ni Sepharose 6 Fast flow resin (GE Healthcare). The unbound tag-free protein was collected, and protein concentration was determined by absorbance (A_280_) using NanoDrop 2000c with the theoretical molar extinction coefficient calculated using the ProtParam tool (ExPASy).

### SDS–PAGE

SDS–PAGE was performed to estimate the purity of the recombinant proteins. Protein samples were denatured by boiling with sample buffer containing SDS, with or without DTT.

### SPR-binding of SIH peptides titrated-RBD complex to immobilized ACE2-hFc

Synthetic ACE2 competing peptides were titrated (1 μM–62.5 nM or 10–0.625 μM) with 100 nM monomeric mRBD. The complexed mixtures were subjected to ACE2-hFc receptor competition studies by SPR on a ProteOn XPR36 Protein Interaction Array v.3.1 (Bio-Rad). An amine coupling GLM sensor chip was activated with sulfo-NHS, EDC (Sigma) reaction. Immunoglobulin capture selective Protein G (Sigma) was covalently coupled to roughly 3,500–4,000 response unit (RU) using 10 mM sodium acetate buffer pH 4.0 at a flow rate of 30 µl min^−1^ for 300 s in the desired channels. Finally, the channels were quenched with 1 M ethanolamine to block the excess sulfo-NHS esters. ACE2-hFc was immobilized to roughly 800 RU at a flow rate of 30 µl min^−1^ for 100 s, excluding a single blank channel that acts as the reference channel. Peptide titrated-RBD complexes were assayed for competitive binding to ACE2-hFc by monitoring binding for 200 s association time and 600 s dissociation time. The channels were preequilibrated for roughly 200–250 s before binding experiments. The reference channel acted as nonspecific binding control. Post each kinetic assay, the channels were regenerated with 0.1 M Glycine-HCl (pH 2.7). Ligand immobilization was performed after each kinetic assay. Various concentration of synthetic peptide (1,000, 500, 250, 125, 62.5 nM) in 1× PBST were titrated with 100 nM of mRBD and used for binding studies. The kinetic traces were obtained and analyzed on Proteon Manager 3.1.

### SPR-direct binding kinetics determination of immobilized RBD to synthetic peptides

Direct binding kinetics to immobilized mRBD were assayed by titration with synthetic ACE2 competing peptides (160–10 nM). Briefly, amine coupling GLM sensor chip was activated with sulfo-NHS, EDC (Sigma) reaction. mRBD was covalently coupled to roughly 3,500 RU using 10 mM sodium acetate buffer pH 4.5 at a flow rate of 30 µl min^−1^ for 300 s in the desired channels. Finally, the channels were quenched with 1 M ethanolamine to block the excess sulfo-NHS esters. Binding assays were performed at a flow rate of 30 µl min^−1^ for 200 s association and 600 s dissociation phases, with a single blank channel that acted as the reference channel. The binding affinity for SIH-5 was calculated considering 200 s (200–400 s) as the dissociation time. The channels were preequilibrated for roughly 200–250 s before binding experiments. The reference channel acted as a nonspecific binding control. Post each kinetic assay, the channels were regenerated multiple times with 4 M MgCl_2_. Various concentrations of synthetic peptide (160, 80, 40, 20, 10 nM) in 1× PBST were used for binding studies. The kinetic traces were obtained and analyzed using Proteon Manager 3.1.

### Analytical ultracentrifugation

#### Sedimentation-velocity analytical ultracentrifugation

The sedimentation-velocity analytical ultracentrifugation data for SIH-3, SIH-5, SIH-7, SIH-11, SIH-13, SIH-14 and SIH-15 were collected between the concentration range of 20–50 µM, and the data for BGF-1 and BGF-2 were collected at 100 μM concentration using an optima XL-I analytical ultracentrifuge equipped with absorbance optics with an An-50Ti 8 place rotor (Beckman Inc.). Peptides were dissolved in the buffer (20 mM Phosphate buffer pH 7.4). Sedimentation-velocity experiments were carried out at 40,000 or 42,000 r.p.m. at 20 °C using two-channel charcoal-filled centerpieces with Sapphire glass windows. Samples were loaded onto the two-sector centerpiece (400 µl of reference cells and 390 µl of sample cells). The velocity data were collected by scanning samples at a wavelength of 280 nm with a spacing of 0.003 cm and an average of three scans per step. The standard partial specific volumes of proteins were taken into a calculation that had a value of 0.73 cm^3^ g^−1^ at 20 °C. Buffer density (roughly 1.00 g cm^−^^3^) and viscosity (roughly 0.01002 poise) were calculated using the program SEDNTERP and these values were kept fixed for all the peptides. Assembly states of peptides were analyzed by direct curve fitting of sedimentation boundaries using Sedfit^48^. Fit to data was selected based on the root mean square deviations (r.m.s.d.) less than 0.008. The sedimentation coefficients were normalized to 20 °C in water, *S*_20,w_ under standard conditions.

#### Sedimentation-equilibrium analytical ultracentrifugation

Sedimentation-equilibrium experiments were carried out at 25,000, 33,000 and 42,000 r.p.m. at 20 °C using six channel charcoal-filled centerpieces with Sapphire windows. The equilibrium data of SIH-5 were collected by scanning samples at 280 nm at a spacing of 0.003 cm with an average of five scans per step. The equilibrium data were edited using WinREEDIT (J. Lary, National Analytical Ultracentrifuge Center) program, and edited data sets were then analyzed using nonlinear least squares using the program WINNONLIN (D. Yphantis, University of Connecticut; M. Johnson, University of Virginia and J. Lary, National Analytical Ultracentrifuge Center).

### nanoDSF thermal melt studies

Equilibrium thermal unfolding of mRBD (−10x His-tag) protein with and without synthetic peptide was carried out using a nanoDSF (Prometheus NT.48) as described previously^39^. The mRBD was incubated with different synthetic helix-hairpin peptides (1:1 stoichiometry) for at least 10 min at room temperature before analysis. Two independent measurements were carried out in duplicate with 10 μM of mRBD protein in the presence or absence of equimolar concentration of peptide using a temperature range of 15–95 °C at 100% LED power and initial discovery scan counts (350 nm) ranging between 5,000 and 10,000.

### SEC–MALS

SEC–MALS of mRBD and the peptide–mRBD complex were performed using PBS (pH 7.4) buffer equilibrated analytical Superdex-200 10/300GL gel filtration column (GE Healthcare) on a Shimadzu HPLC. The mRBD and synthetic peptides were complexed in equimolar amounts (30:30 µM), 60 min at 4 °C before the assay. However, due to the low solubility of SIH-1 in phosphate buffer, the mRBD was incubated with SIH-1 at 12 µM concentration to obtain equimolar amounts (12:12 µM). Gel filtration resolved protein peaks were subjected to in-line refractive index (Waters Corp.) and MALS (mini DAWN TREOS, Wyatt Technology Corp.) detection for molar mass determination. The acquired data from UV, MALS and refractive index (RI) were analyzed using ASTRA 6.1 software (Wyatt Technology).

### Negative staining–TEM (NS–TEM) sample preparation and data acquisition

Freshly prepared spike protein and SIH-5 samples were incubated in the ratio 1:3 (spike protein:SIH-5) for 20 min at 25 °C. The reaction mixture was analyzed for the presence of uniformly distributed homogeneous spike protein–SIH-5 complexes using conventional NS–TEM as previously described^35^. In brief, carbon-coated Cu grids were hydrophilized for 30 s using a GloQube glow discharge system before the sample application. 3.5 μl of protein-peptide sample (roughly 0.1 mg ml^−1^) was applied on the glow discharged grids and incubated at room temperature for 1.5 min. The surplus sample was gently blotted off using Whatman filter paper, and negative staining was performed for 30 s with 1% fresh uranyl acetate solution. The stained grids were observed, and data were acquired at room temperature using a Talos L120C transmission electron microscope (Thermo Fisher Scientific) equipped with a Ceta (4,000 × 4,000 K) camera. Images were recorded with calibrated pixel sizes of 2.38 Å per pixel.

#### NS–TEM data processing

The negative staining electron micrographs were assessed using the EMAN v.2.1 software package. Spike protein–SIH-5 peptide complexes were picked manually as well as automatically. Coordinates of the 1,500 selected particles were extracted using e2boxer.py in EMAN v.2.1 software. Reference-free 2D classification was performed using simple_prime2D of the SIMPLE v.2.0 software package.

### Cryo-EM sample preparation and data collection

Quantifoil R1.2/1.3 (Electron Microscopy Sciences) 300 mesh gold grids were glow discharged for 130 s at 20 mA in GloQube glow discharge system (Gatan Inc.) before sample application. Here, 3 μl of freshly prepared spike protein–SIH-5 sample was applied onto the glow discharged holey grids and incubated at room temperature for 10 s. The excess sample was blotted for 8 s at 100% humidity, followed by rapid plunging into liquid ethane using an FEI Vitrobot Mark IV plunger. Cryo-EM data were captured in Thermo Scientific 200 kV Talos Arctica transmission electron microscope (FEI) equipped with K2 Summit Direct Electron Detector (Gatan Inc.). Data were acquired using automated data collection software LatitudeS (Gatan Inc.) at a magnification of ×42,200 and pixel size 1.17 Å at specimen level. 80 e^−^/Å^2^ total electron dose over defocus ranging from −0.75 to −2.5 μm was subjected to the sample. Data were recorded for a total of 20 frames for 8 s.

#### Cryo-EM data processing

Cryo-EM raw movie files were first imported into RELION v.3.1, and beam-induced motion correction was performed using MotionCor2 software. The motion-corrected images were then imported into cisTEM software^49^, and only data up to 8 Å resolution were screened and selected for further processing. A total of 4,794 of the best micrographs were processed using RELION v.3.1 software. The contrast transfer function of these micrographs was estimated using RELION implementation of CTFFIND v.4.1.13. Following an initial round of manual picking, reference-free 2D classification was performed to use as a reference for automated particle picking. A total of 1,057,624 particle coordinates were extracted at a box size of 600 pixels binned by 2, yielding a pixel size of 2.34 Å. The dataset was initially screened robustly by performing three rounds of 2D classification. The best classes from 2D classification were selected, and 376,308 particles were split into three classes for 3D classification (without imposing any symmetry, C1 symmetry).

Class 2 (101,296 particles) was selected for further refinement. The selected particle set for Class 2 was extracted at a box size of 600 pixels yielding a pixel size of 1.17 Å. Further auto-refinement was performed using a soft mask generated against dimeric spike molecules. The refined map sharpened with a *B* factor of −120 was resolved at 6.3 Å resolution. The particles were further subjected to contrast transfer function refinement with correction for beam-tilt fitting, anisotropic magnification correction and per-particle defocus. This was followed by Bayesian polishing. The ‘shiny’ particles obtained were subjected to another round of auto-refinement with a soft mask followed by map sharpening with a *B* factor of −120 and resolved at 5.4 Å resolution. Local resolution estimation was performed using unfiltered auto-refined maps with ResMap.

To understand the structural details of spike protein and SIH-5 interaction, masked auto-refinement was performed based on a single spike protein. For masked refinement, a soft mask was applied on a single spike protein with a 3-RBD up SIH-5 bound state (101,296 particles), at a pixel size of 1.17 Å with a box size of 256 pixels. The auto-refined map was sharpened and resolved at 4.8 Å resolution. The particles were further subjected to contrast transfer function refinement with correction for beam-tilt fitting, anisotropic magnification correction and per-particle defocus. This was followed by Bayesian polishing. The ‘shiny’ particles obtained were subjected to another round of auto-refinement with a soft mask followed by map sharpening with a *B* factor of −120 and resolved at 4.47 Å resolution. For atomic model building, the 3D auto-refined map was sharpened using PHENIX. Local resolution estimation was performed using unfiltered auto-refine maps with ResMap.

#### Atomic model building and structural analysis

SIH-5 bound 3-RBD up single S protein map resolved at 4.47 Å was docked with the Protein Data Bank (PDB) 7KMS (ref. ^50^) (for spike trimer), and rigid body fitting was performed in UCSF Chimera. PDB 7KMS was taken as the initial model to perform phenix.dock_in_map in PHENIX followed by refinement with respect to the cryo-EM map using phenix.real_space_refine. Then, the homology model for SIH-5 was fitted manually in UCSF Chimera. The whole model combining spike and peptide was docked in the cryo-EM map using phenix:dock_in_map followed by refinement with respect to the cryo-EM map using phenix:real_space_refinement. The refined atomic model and the cryo-EM density map were imported to COOT, and the side chain fit was corrected by manual fitting. After every round of fitting in COOT, the model was refined in PHENIX using phenix.real_space_refine. The final atomic model used for assessment was analyzed using MolProbity and validated using phenix:validation_tool in PHENIX.EM ringer score^51^ was calculated using PHENIX. All structural assessments and representations were done using UCSF Chimera and UCSF Chimera X.

### SARS-CoV-2 pseudovirus preparation and entry-inhibition assay

Pseudoviral entry-inhibition/neutralization assays were performed with SARS-CoV-2 pseudovirus described earlier^39^. Briefly, human embryonic kidney 293T (HEK293T) cells were transiently transfected with plasmid DNA pHIV-1 NL4·3Δenv-nanoLuc and Spike-Δ19-D614G by using the Profection mammalian transfection kit (Promega Inc.) following the instructions in the kit manual. Pseudovirus decorated with SARS-CoV-2 spike proteins were harvested 48 h after transfection, clarified by filtration via 0.45-μm filters and stored at −80 °C until further use. 293T cells expressing human ACE-2 (293T-hACE-2) (BEI Resources, NIH, catalog no. NR-52511) were cultured in DMEM (Gibco) supplemented with 5% FBS (fetal bovine serum) and penicillin-streptomycin (100 U ml^−1^).

Entry-inhibition assays were done three times in two replicates by using synthetic peptides. The pseudovirus was incubated with serially diluted peptide in a total volume of 100 μl for 1 h at 37 °C as a standard assay format. The 293T-hACE2 cells were then trypsinized, and 1 × 10^4^ cells per well were added to make up the final volume of 200 μl per well. The plates were further incubated for 48 h in a humidified incubator at 37 °C with 5% CO_2_. The time-course entry-inhibition assay was performed by incubating the virus with peptides for 0, 10 and 30 min. The 293T-hACE2 cells were plated in a 96-well plate a day before, containing 100 μl of growth media per well. The virus and peptide mixture was added onto the cells after the incubation period, and the plate was further incubated for 48 h: 0 min was indicated when no incubation of the virus with peptide was done. After 48 h of incubation of cells, 140 μl of cell culture media was removed and 50 μl of nano-Glo luciferase substrate (Promega Inc.) was added. After lysing the cells for 2–3 min, 80 μl of lysate was transferred to white plates and luminescence was measured using a Cytation-5 multi-mode reader (BioTek Inc.) The luciferase activity measured as Relative luminescence units from SARS-CoV-2 pseudovirus in the absence of peptide was considered as 100% infection and was used as a reference to calculate the percentage inhibition in the presence of peptides. The inhibitory concentration (IC_50_) at which 50% inhibition of viral entry was seen was calculated.

### Pseudovirus preparation and DLS measurements

Exponentially growing HEK293T cells were transfected at the cell density of 50% by using SARS-CoV-2 spike and HIV-1-NL4.3 expression constructs. After 12 h of transfection, the media was removed, cells were washed with plain DMEM and fresh FBS-free DMEM containing penicillin-streptomycin was added. After 30 h of further incubation, virus supernatant was harvested, spun to remove cell debris and filtered through a 0.22 µM filter to obtain a clean virus supernatant. Pseudovirus production was examined by infectivity in HEK293T-hACE2 cells.

The freshly prepared virus was incubated at 37 °C with 200 nM of LCB1/SIH-5/SIH-11 for 1 h. After incubation, the virus-peptide suspension was used for virus size determination. The average particle size distribution of virion-peptide complexes and their zeta potential was determined by a Malvern Zetasizer Nano ZS DLS system. All DLS data were collected and analyzed using Malvern Zetasizer software (v.8.01). The average diameters of pseudovirus or pseudovirus–peptide complexes were calculated as the mean size of particle population ± s.d. from three independent experiments.

### Cell viability assay

The cell viability assay was performed by using the MTT (3-(4,5-dimethylthiazol-2-yl)-2,5-diphenyltetrazolium bromide) assay. Briefly, Vero-E6 or A549 cells were plated at 20,000 per well in 100 μl of growth medium (DMEM with 5% FBS and penicillin-streptomycin) in 96-well microtiter plates for 16–24 h at 37 °C in a humidified chamber with 5% CO_2_. The next day, 50 μl of media was removed from each well and 50 μl of the peptide was added in a dose-dependent manner. 10% DMSO (final concentration v/v) was used as a positive control for the induction of cytotoxicity. The plate was further incubated for 48 h (37 °C, 5% CO_2_), and 10 μl of MTT solution was added to each well. The plate was incubated for another 4 h in the humidified CO_2_ incubator, after which 100 μl of dissolving solution (10% SDS in 0.01 M HCl) was added to each well. The plate was then incubated in a CO_2_ incubator overnight. The next day, the plate was read for absorbance at 570 nm. The percentage viability of cells was recorded in comparison to the untreated cells.

### Hamster experiments

Regarding ethics, all animal experiments were performed in the Virus BSL-3 Laboratory at the Centre for Infectious Disease Research, Indian Institute of Science, Bangalore, India, following CPCSEA (The Committee for the Purpose of Control and Supervision of Experiments on Animals) and ARRIVE guidelines^52^. The work plans were reviewed and approved by the Indian Institute of Science, Institute Animal Ethical Committee. Required number of Syrian golden hamsters (*Mesorectums auratus*) of both sexes (80–100 gm of weight) were procured from CPCSEA registered, Biogen Laboratory Animal Facility (Bangalore, India). The hamsters were housed in individually ventilated cages, maintained at 23 ± 1 °C temperature and 50 ± 5% relative humidity.

#### Prophylactic treatment of peptide

After acclimatization of 7 d in individually ventilated cages at the IISc Virus BSL-3 Laboratory, the hamsters were divided into three groups: (1) control-PBS treated (*n* = 3), (2) challenged with 5 × 10^6^ PFU dose of SARS-CoV-2 (*n* = 5) and (3) SIH-5 treated (day 0 at −8 h) and challenged (*n* = 5). After taking the basal weight of all groups, hamsters were anesthetized with xylazine (10 mg kg^−1^ body weight) and ketamine (150 g kg^−1^ per body weight) cocktail intraperitoneally. Hamsters were treated intranasally (i.n.) either with a dose of 2.5 mg kg^−1^ SIH-5 in 100 μl of PBS or with PBS for the control group. Hamsters were challenged, 8 h later, with 100 μl of i.n. administered SARS-CoV-2 US strain (USA-WA1/2020 obtained from BEI resources) at 5 × 10^6^ PFU per hamster, by sedating/anesthetizing the hamsters with xylazine (10 mg kg^−1^ body weight) and ketamine (150 g kg^−1^ body weight) cocktail intraperitoneally. In addition, the control group of three hamsters were dosed similarly with PBS. The health of hamsters, body temperatures, body weights and clinical signs were monitored daily by an expert veterinarian. Clinical sign scoring systems were developed following the previous studies^19^ with some modifications. In the present experiment considering 14 clinical signs, we measured average clinical scores based on the points system as follows, lethargy (1 point), rough coat (1 point), sneezing (1 point), mucus discharge from nose or eyes (1 point), half-closed/watery eyes (1 point), huddling in the corner (1 point), ear laid back (1 point) hunched back (1 point), head tilt (1 point), moderate dyspnea (2 points) and body weight loss 2–5% (1 point), 5–10% (2 points) and 10–20% (3 points) and shaking or shivering (1 point).

On the fourth day after the challenge, all the hamsters were humanely euthanized by an overdose of xylazine through intraperitoneal injection. The left lobe of the lung was harvested and fixed in 4% paraformaldehyde for histopathological examination of the lungs. The right lobes were frozen at −80 °C for determining the virus copy number by quantitative PCR with reverse transcription (RT–qPCR).

#### Histopathological examination

After necropsy, the left lobe of the lung of each hamster was fixed in 4% of paraformaldehyde, and the next day, these were processed, embedded in paraffin and cut into 4 µm sections by microtome for hematoxylin and eosin staining. The lung sections were microscopically examined and evaluated for different pathological scores by a veterinary immunologist. Four different histopathological scores were assigned as follows: (1) percentage of the infected part of lung tissues considering the consolidation of lung, (2) lung inflammation scores, considering the severity of alveolar and bronchial inflammation, (3) immune cell influx score, considering the infiltration of lung tissue with the numbers of neutrophils, macrophages and lymphocytes and (4) edema score, considering the alveolar and perivascular edema. The scores and parameters were graded as absent (0), minimal (1), mild (2), moderate (3) or severe (4)^53^.

#### RNA extractions and RT–qPCR to quantitate subgenomic virus copy in lungs

The freeze–thawed right lower lobe of each hamster lung was homogenized in 1 ml of RNAiso Plus Reagent (Takara), and total RNA was isolated as per the manufacturer’s protocol. The quantity and quality (260/280 ratios) of RNA extracted was measured by NanoDrop. The extracted RNA was diluted to 27 ng μl^−1^ in nuclease-free water. The viral subgenomic copy number was quantified using 100 ng of RNA per well for 10 μl of the reaction mixture using AgPath-ID One-Step RT–PCR kit (AM1005, Applied Biosystems). The following primers and probes were used 2019-nCoV_N1-Fwd- 5′-GACCCCAAAATCAGCGAAAT-3′; 2019-nCoV_N1-Rev- 5′-TCTGGTTACTGCCAGTTGAATCTG-3′; 2019-nCoV_N1 Probe (6-FAM/BHQ-1) ACCCCGCATTACGTTTGGTGGACC (Sigma Aldrich) for targeting SARS-CoV-2 N-1 gene. The subgenomic virus copy number per 100 ng of RNA was estimated by generating a standard curve from serial dilution of SARS-CoV-2 genomic RNA standard.

### Statistical analysis

Data are described as mean ± s.d. unless otherwise stated. The *P* value for hamster weight change between virus challenged and SIH-5 treated was analyzed by a two-way analysis of variance test using the GraphPad Prism software. The *P* values for viral load in the lungs and histopathological scores were analyzed by a two-tailed Mann–Whitney test using the GraphPad Prism software (v.8.0.1).

### Reporting summary

Further information on research design is available in the Nature Research Reporting Summary linked to this article.

## Online content

Any methods, additional references, Nature Research reporting summaries, source data, extended data, supplementary information, acknowledgements, peer review information; details of author contributions and competing interests; and statements of data and code availability are available at 10.1038/s41589-022-01060-0.

## Supplementary information

Supplementary Information Supplementary Figs. 1–20, Tables 1–4, Note and References.

Reporting Summary

Supplementary Video 1 Cryo-EM 3D model of a single 3-RBD up spike protein bound to SIH-5.

## Data Availability

The cryo-EM maps and atomic models have been deposited in the Electron Microscopy Data Bank and the PDB with accession codes: EMD-32388, EMD-33042 and PDB entry ID 7X7N. All other data are present as source data provided with this paper or in the Supplementary Information.

## Source data

Source Data Fig. 2 Statistical source data.
Source Data Fig. 4 Statistical source data.
Source Data Fig. 5 Statistical source data.
Source Data Fig. 6 Statistical source data.
Source Data Extended Data Fig. 2 Statistical source data.
Source Data Extended Data Fig. 3 Statistical source data.
Source Data Extended Data Fig. 6 Statistical source data.
Source Data Extended Data Fig. 7 Statistical source data.

## Extended data

are available for this paper at 10.1038/s41589-022-01060-0.
