# Supplementary material for: A dimeric proteomimetic prevents SARS-CoV-2 infection by dimerizing the spike protein
Source: Nat Chem Biol. 2022 Jun 2;18(10):1046–55. doi: 10.1038/s41589-022-01060-0 (PMC9512702; doi:10.1038/s41589-022-01060-0)
Supplement: Supplementary file 1 — Supplementary Figs. 1–20, Tables 1–4, Note and References. [file 41589_2022_1060_MOESM1_ESM.pdf]

nature portfolio

https://doi.org/10.1038/s41589-022-01060-0

Supplementary information

A dimeric proteomimetic prevents SARS-CoV-2 infection by dimerizing the spike protein

In the format provided by the authors and unedited

Supplementary Information

A dimeric proteomimetic prevents SARS-CoV-2 infection by dimerizing the spike protein

Bhavesh Khatri[1], Ishika Pramanick[1⊥], Sameer Kumar Malladi[1⊥], Raju S Rajmani[1], Sahil Kumar[2], Pritha Ghosh[1], Nayanika Sengupta[1], R. Rahisuddin[3], Narender Kumar[3], S. Kumaran[3], Rajesh P Ringe[2], Raghavan Varadarajan[1], Somnath Dutta*[1], Jayanta Chatterjee*[1]

⊥ Equally contributed to the work

[1]Molecular Biophysics Unit (MBU), Indian Institute of Science, Bangalore 560012, India.

[2]Virology Unit, Institute of Microbial Technology, Council of Scientific and Industrial Research (CSIR), Chandigarh 160036, India.

[3]Institute of Microbial Technology, Council of Scientific and Industrial Research (CSIR), Chandigarh 160036, India.

Table of Contents:

Supplementary Figures 1-20 .... S1
Supplementary Tables 1-4 .... S31
Supplementary Note S35
Supplementary References S53

Supplementary Figures

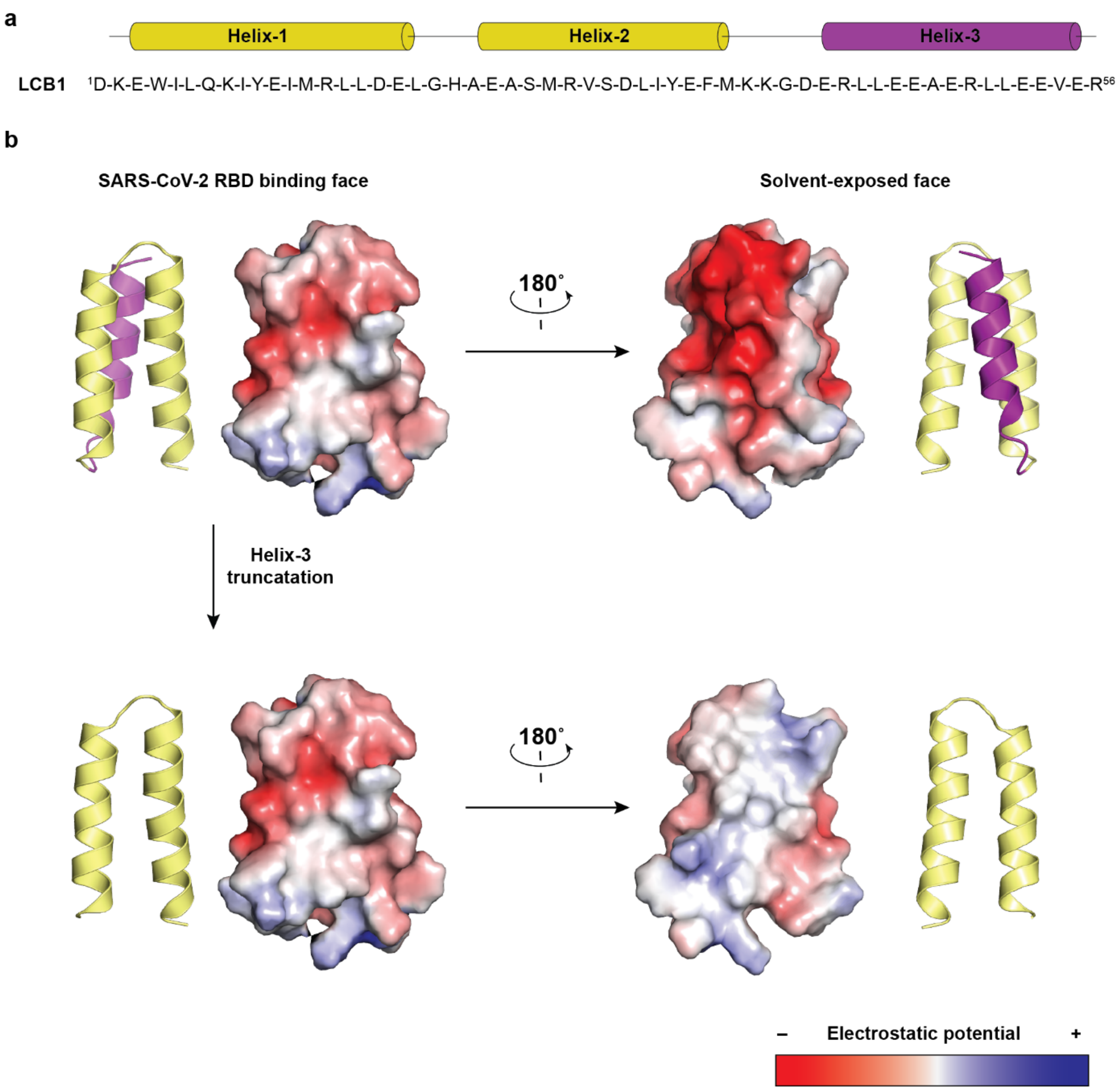

Supplementary Fig. 1. In silico analysis of LCB1. a, Sequence and secondary structure map of three-helix bundle LCB1 miniprotein. **b**, Electrostatic surface representation of LCB1. The surface is colored according to its electrostatic potential from red (negatively charged) to blue (positively charged). The buried hydrophobic surface from helix-1 and helix-2 (exoface) gets exposed upon deletion of helix-3. The figure was generated using Pymol.

a

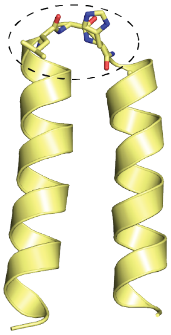

Residue	ϕ (°)	ψ (°)
Leu^{19} (γ)	-98.7	-1.3
Gly^{20} (α_L)	79.2	25.4
His^{21} (β)	-118.2	76.9

b

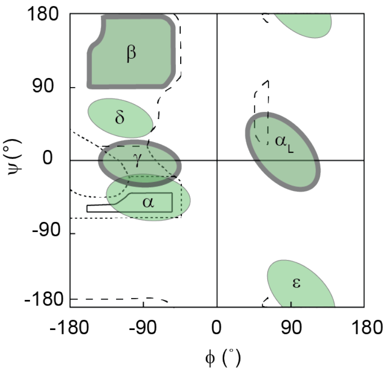

c

— Helix-1 —Yα_L-β— Helix-2 —		Helicity (%)	$[\theta]_{222}/[\theta]_{208}$	T_M (°C)	Competitive Inhibition* (IC_{50}, nM)
**LCB1**	^{1}DKEWILQKIYEIMRLLDELGHAEASMRVSDLIYEFMKK^{38} GDERLLEEAERLLEEVER^{56} (**Helix-3**)	56.8	1.02	>95	125-250
**SIH-1**	^{1}DKEWILQKIYEIMRLLDELGHAEASMRVSDLIYEFMKK^{38}	52.9	1.07	75.5 ± 0.8	2500-5000
**SIH-2**	^{1}DKEWILQKIYEIMRLLDELGHBEASMRVSDLIYEFMKK^{38}	45.9	1.06	80.7 ± 0.6	1250-2500
**SIH-3**	^{1}DKEWILQKIYEIMRLLDELaHBEASMRVSDLIYEFMKK^{38}	62.3	1.13	>95	125-250
**SIH-4**	^{1}DKEWILQKIYEIMRLLDELaNBEASMRVSDLIYEFMKK^{38}	78.4	1.14	>95	250-500
**SIH-5**	^{1}DKEWILQKIYEIMRLLDELaDBEASMRVSDLIYEFMKK^{38}	67.8	1.13	>95	125-250
**SIH-6**	^{1}DKEJILQKIYEIMRLLDELaDBEASMRVSDLIYEFMKK^{38}	53.8	1.21	83.6 ± 1.1	500-1000
**SIH-7**	^{1}DKEWILQKIOEIMRLLDELaDBEASMRVSDLIYEFMKK^{38}	56.8	1.08	>95	125-250
**SIH-8**	^{1}DKEWILQKIJ'EIMRLLDELaDBEASMRVSDLIYEFMKK^{38}	56.3	1.28	>95	250-500
**SIH-9**	^{1}DKEWILQKIYEIURLLDELaDBEASMRVSDLIYEFMKK^{38}	53.0	1.16	>95	125-250
**SIH-10**	^{1}DKEWILQKIYEIMRXLDELaDBEASMRVSDLIYEFMKK^{38}	60.5	1.15	>95	250-500
**SIH-11**	^{1}DKEWILQKIYEIMRLLDELaDBEASMRVSDLIYEZMKK^{38}	61.5	1.06	>95	125-250
**SIH-12**	^{1}DKEWILQKIYEIMRLLDELaDBEASMRVSDLIYEFUKK^{38}	43.0	1.10	>95	250-500
**SIH-13**	^{1}DKEWILQKIOEIMRLLDELaDBEASMRVSDLIYEZMKK^{38}	50.7	0.93	>95	125-250
**SIH-14**	^{1}DKEWILQKIYEAMRLLDELaDBEASMRVSDAIYEFMKK^{38}	59.8	1.07	>95	250-500
**SIH-15**	^{1}DKEWILQKIYEEMRLLDELaDBEASMRVSDEIYEFMKK^{38}	41.2	0.89	>95	250-500

d

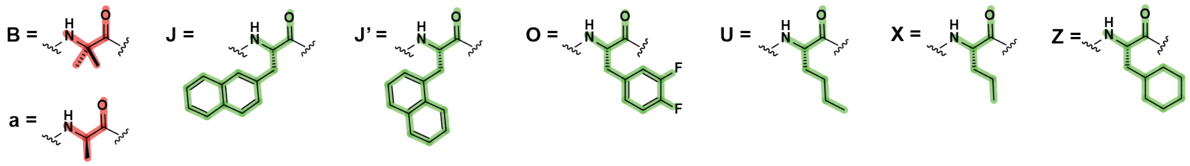

Supplementary Fig. 2. Engineering the interhelical loop and residues in helices 1 and 2. a, Truncated LCB1 with the -γα_Lβ- conformation of loop residues Leu^{19}, Gly^{20}, and His^{21}, respectively. **b**, The Ramachandran map depicting the various conformation zones. **c**, Sequences, helicity, thermal stability, and competitive potency against ACE2 in binding to RBD of LCB1 and helix-hairpin peptides. **d**, Structure of unnatural amino acids, D-Alanine (**a**), Aminoisobutyric acid (**B**), 3-(2-Naphthyl)-L-alanine (**J**), 3-(1-Naphthyl)-L-alanine (**J'**), *3,4*-Difluoro-L-Phenylalanine (**O**), L-Norleucine (**U**), L-Norvaline (**X**) and L-Cyclohexylalanine (**Z**). (Helicity was measured at 20 °C in sodium-phosphate buffer, pH 7.4). All the synthesized SIH analogs are given, a subset of which is present in **Fig. 1**.

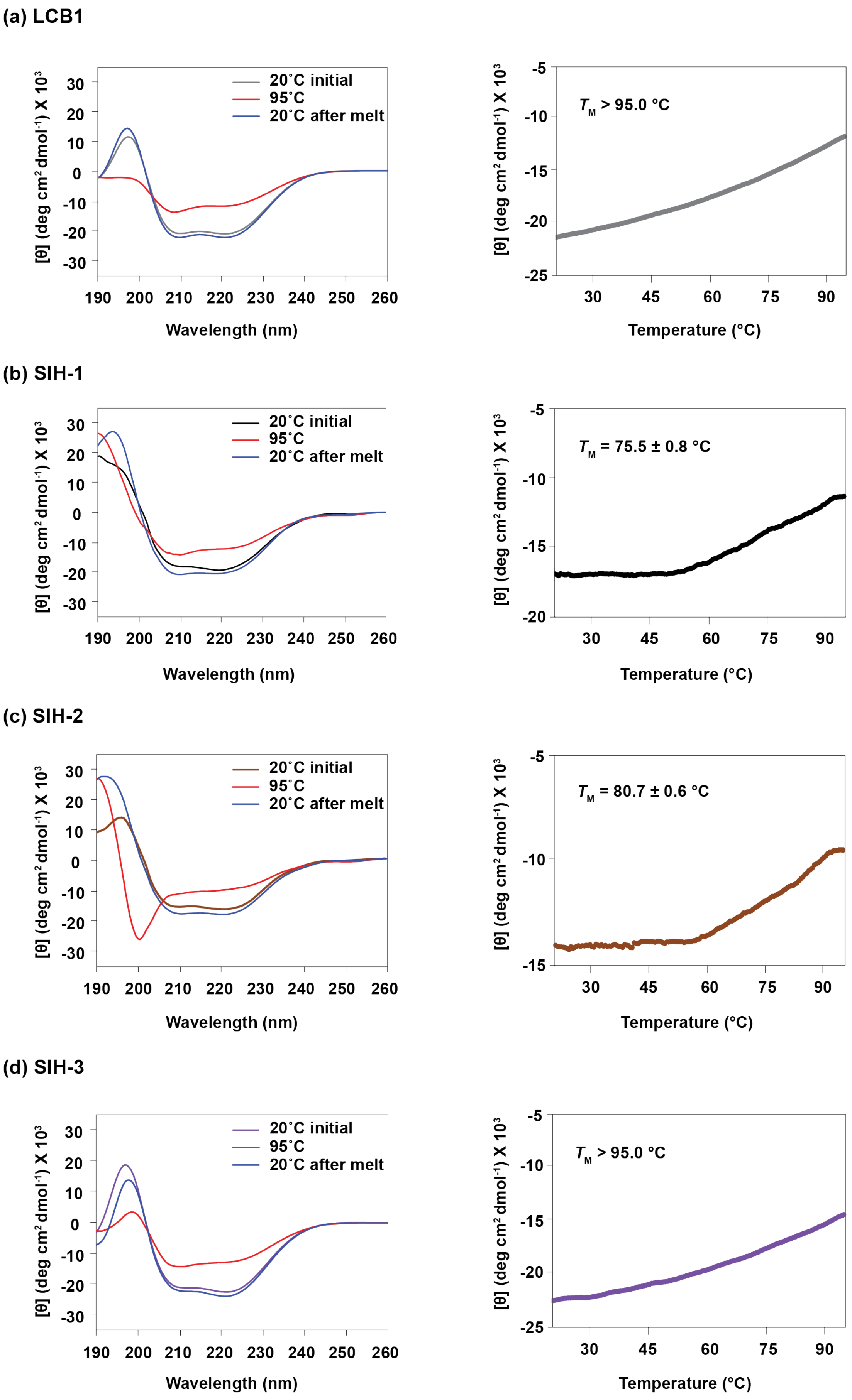
(a) LCB1
(b) SIH-1
(c) SIH-2
(d) SIH-3
20˚C initial
95˚C
20˚C after melt
[θ] (deg cm² dmol⁻¹) X 10³
Wavelength (nm)
Temperature (°C)
T_M > 95.0 °C
T_M = 75.5 ± 0.8 °C
T_M = 80.7 ± 0.6 °C
T_M > 95.0 °C

(e) SIH-4

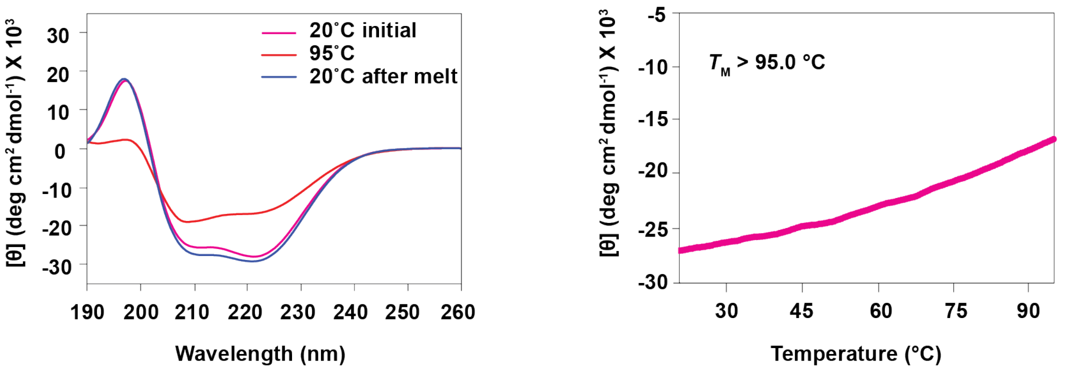

(f) SIH-5

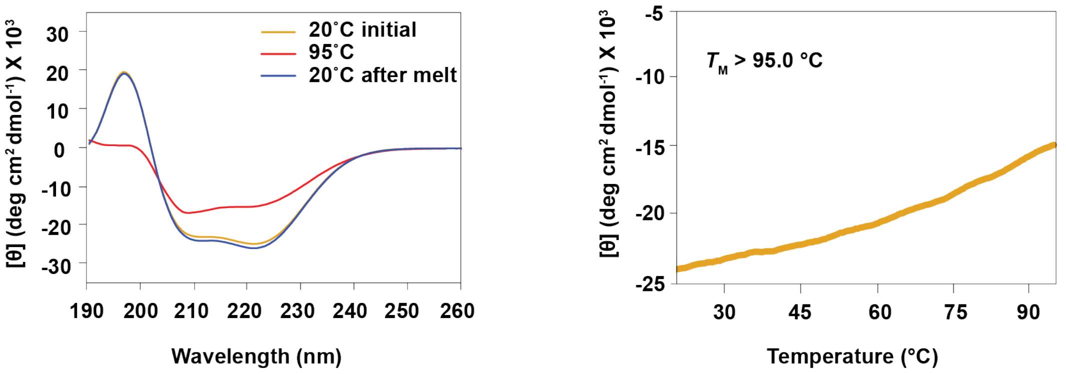

(g) SIH-6

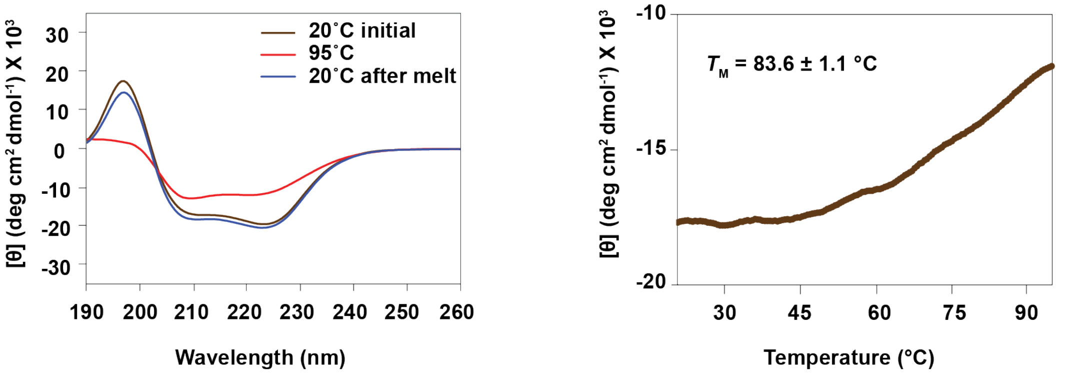

(h) SIH-7

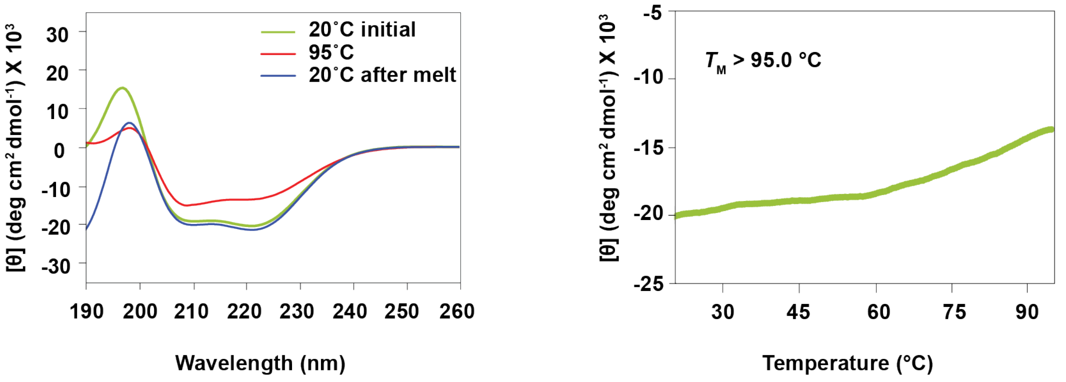

(i) SIH-8

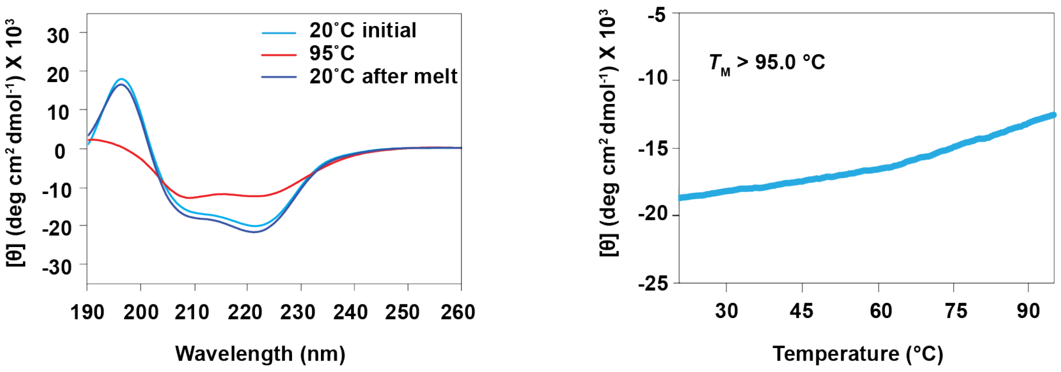

(j) SIH-9

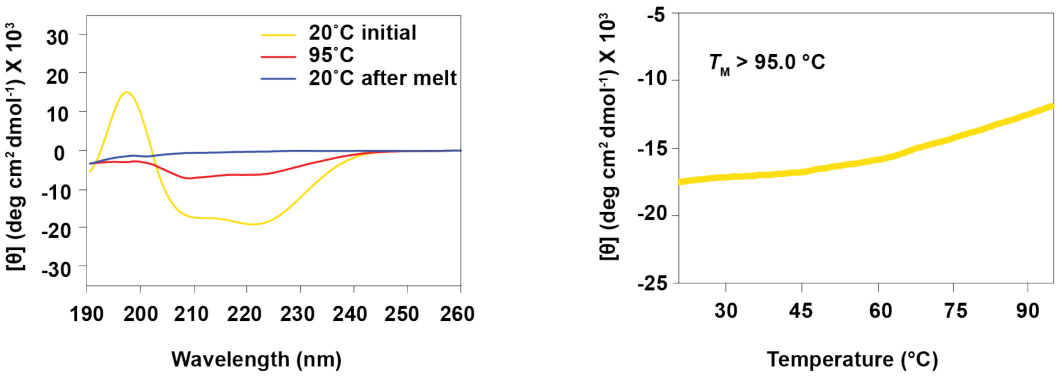

(k) SIH-10

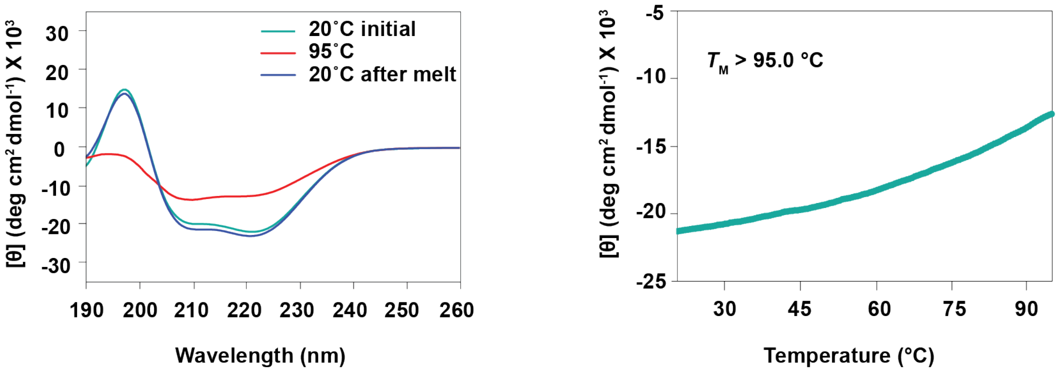

(l) SIH-11

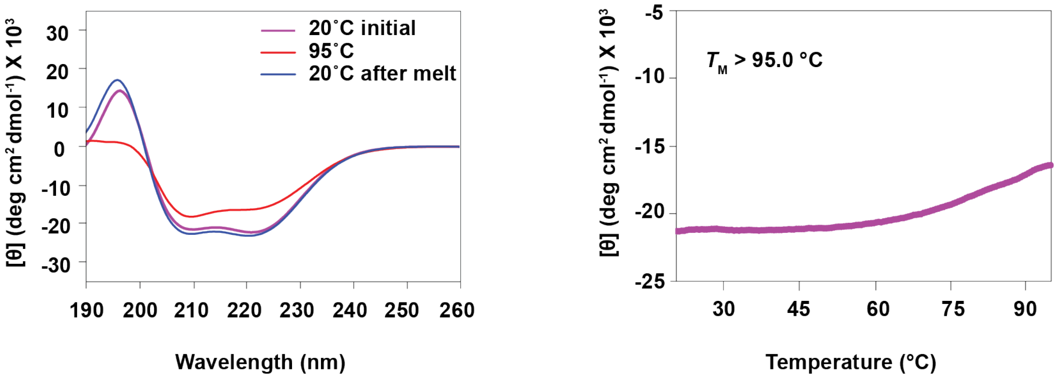

(m) SIH-12

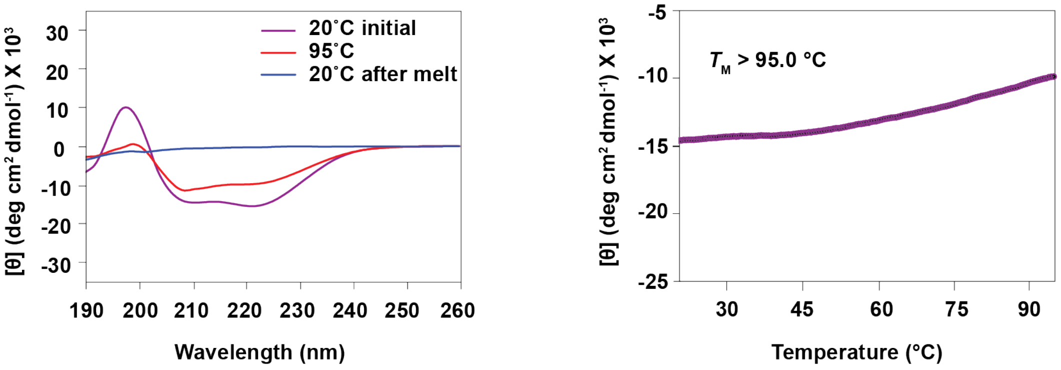

(n) SIH-13

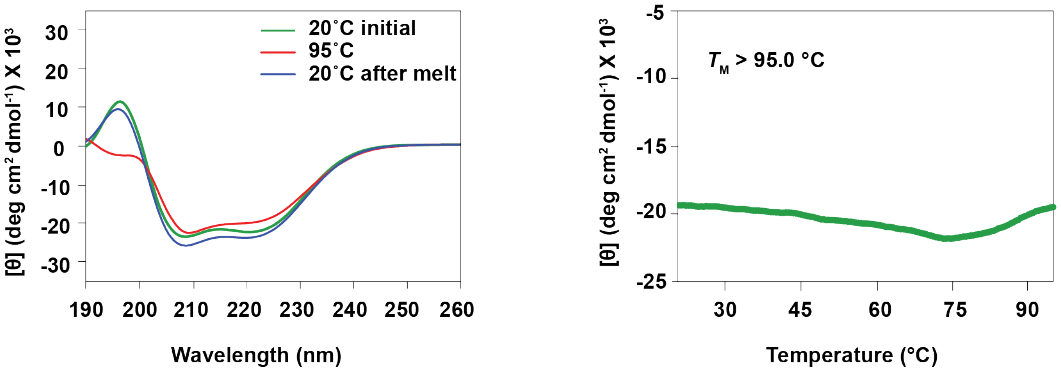

(o) SIH-14

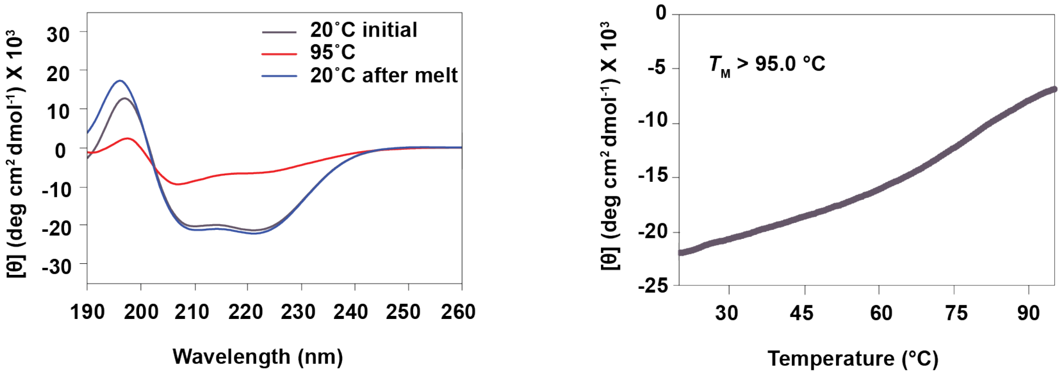

(p) SIH-15

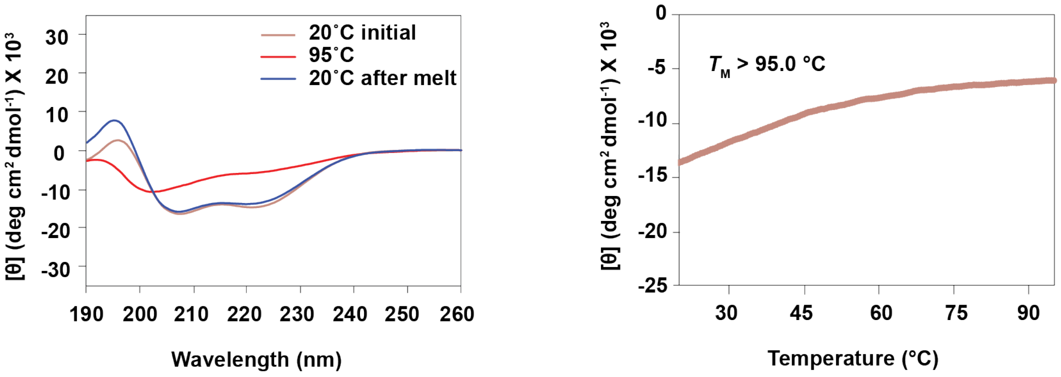

Supplementary Fig. 3. Thermal stability and reversibility of LCB1 and helix-hairpin peptides. Circular dichroism (CD) spectra of the synthetic peptides at different temperatures (left panel). The structural reversibility after removal of thermal stress is observed by comparing the CD spectrum at 20 °C and 20 °C after melt. CD thermal unfolding was monitored at 222 nm (right panel). Data were fit to a two-state unfolding model to obtain the melting temperature. The data for all the analogs are presented for comparison and clarity.

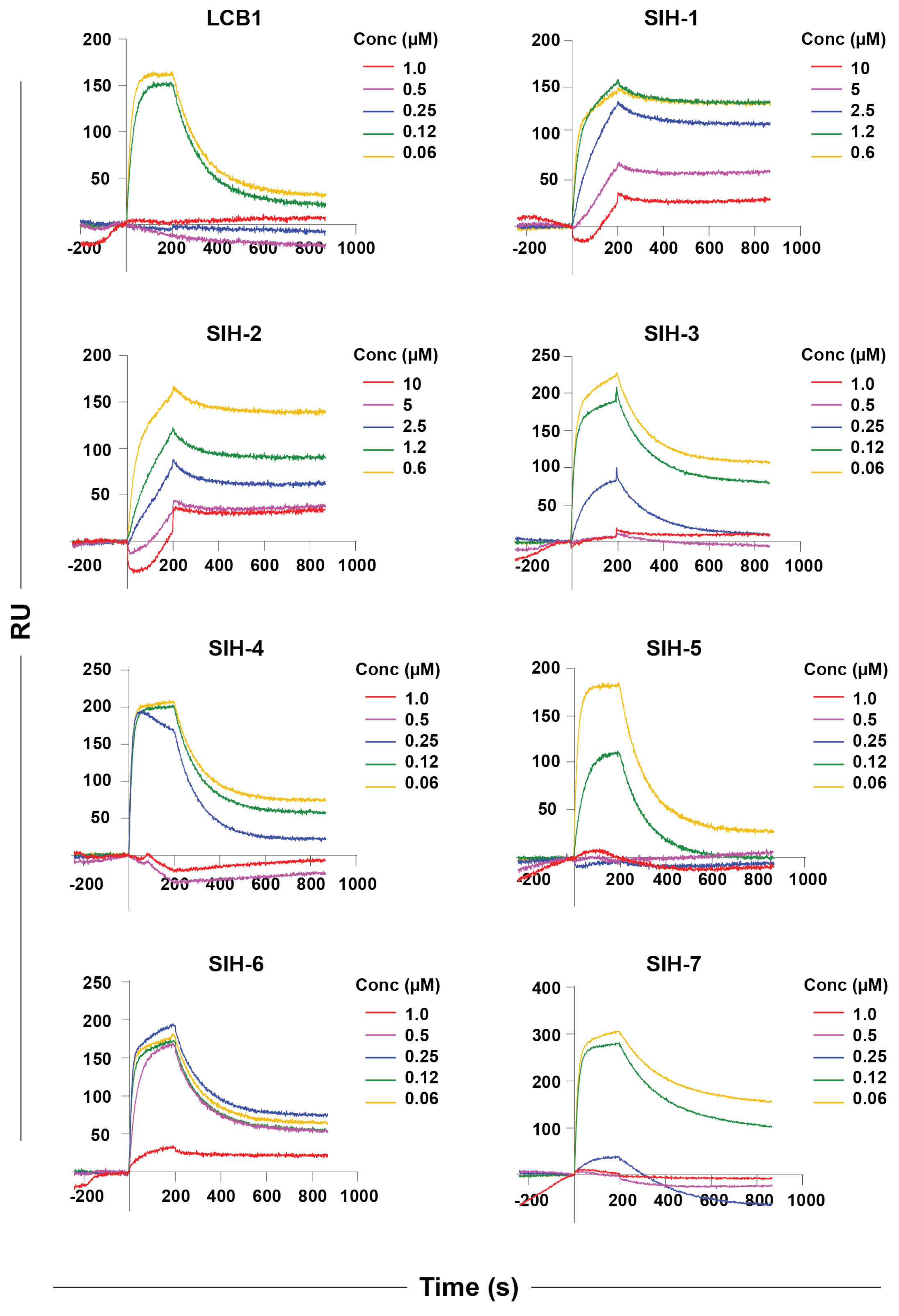
LCB1
SIH-1
SIH-2
SIH-3
SIH-4
SIH-5
SIH-6
SIH-7
Conc (μM)
1.0
0.5
0.25
0.12
0.06
10
5
2.5
1.2
0.6
RU
Time (s)

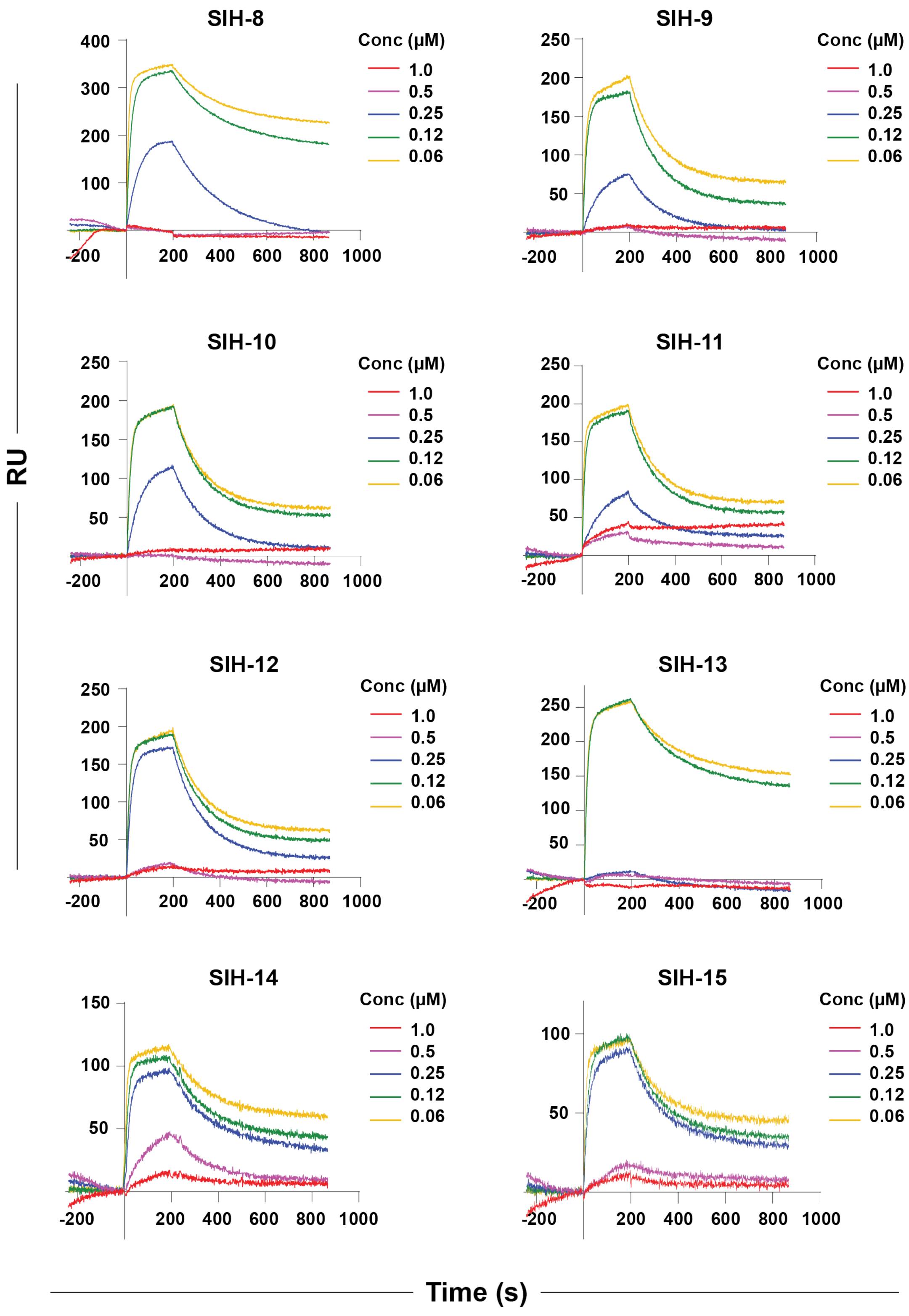

Supplementary Fig. 4. Competitive binding assay. Competitive inhibition of SARS-CoV-2 RBD binding to the immobilized ACE2 by LCB1 and the helix-hairpin peptides monitored by SPR. A subset of data from the main figures and extended data figures are replicated to present data for all the analogs for comparison and clarity.

(a) SIH-3

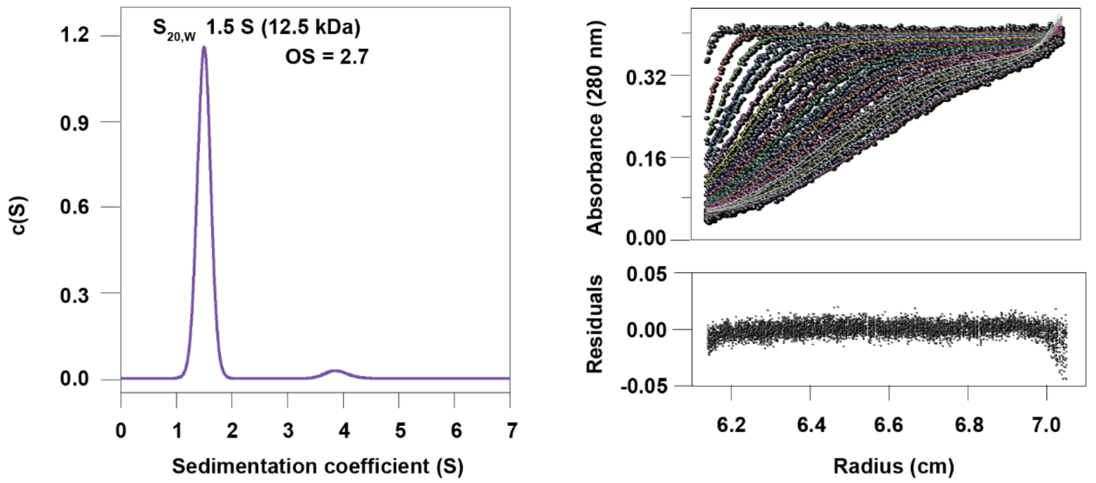

(b) SIH-5

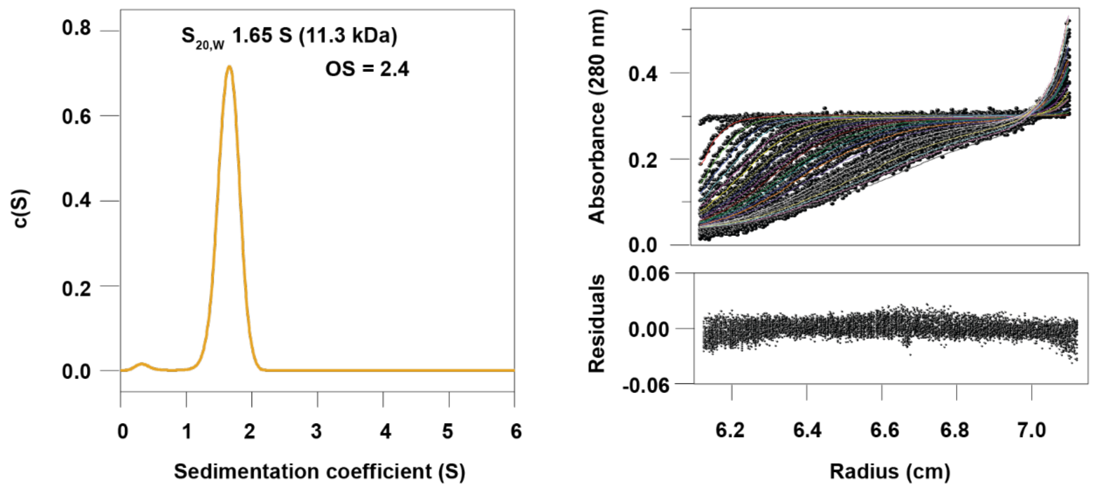

(c) SIH-7

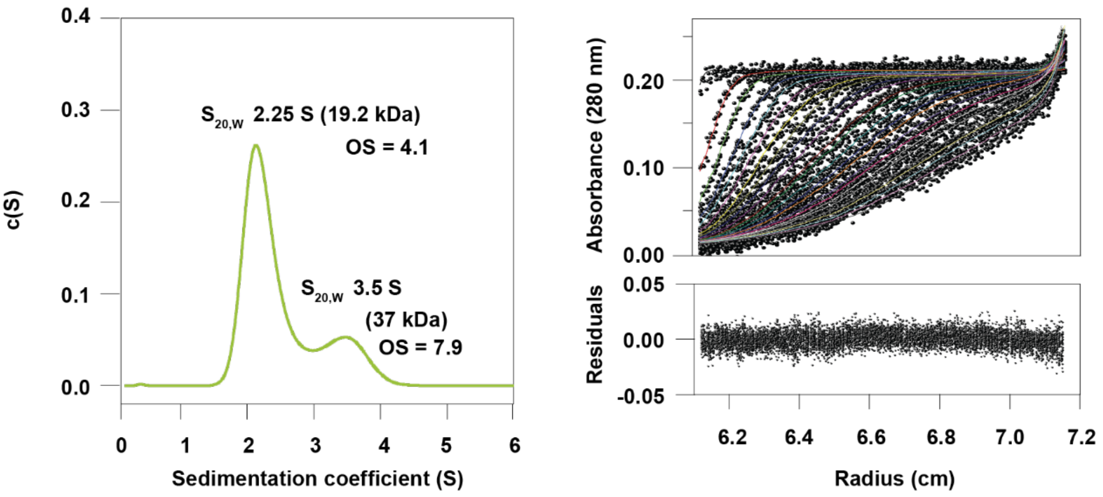

(d) SIH-11

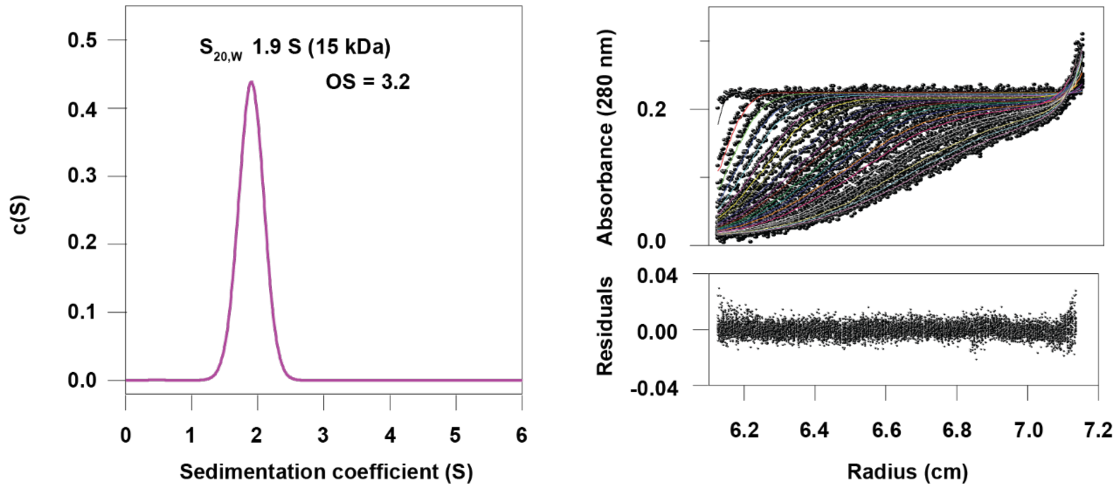

(e) SIH-13

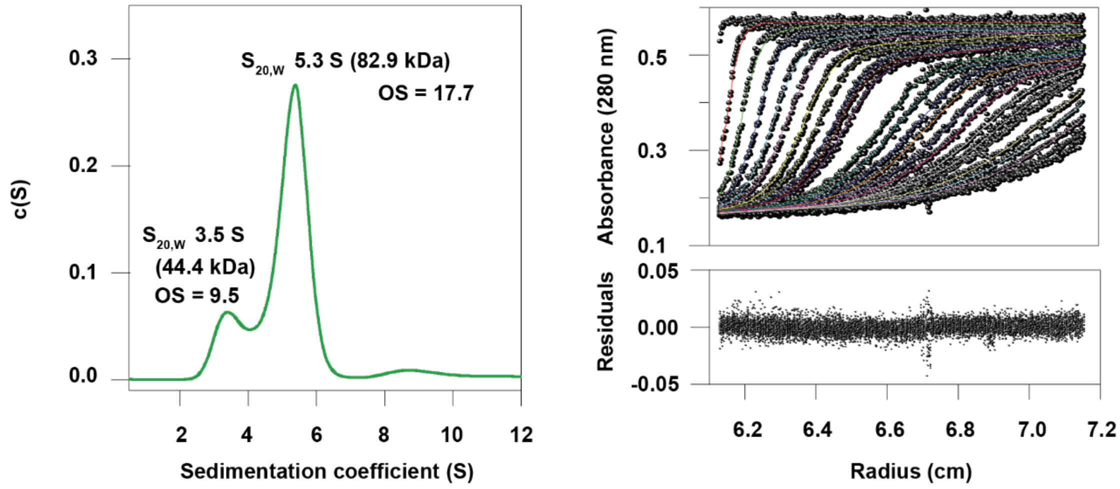

(f) SIH-14

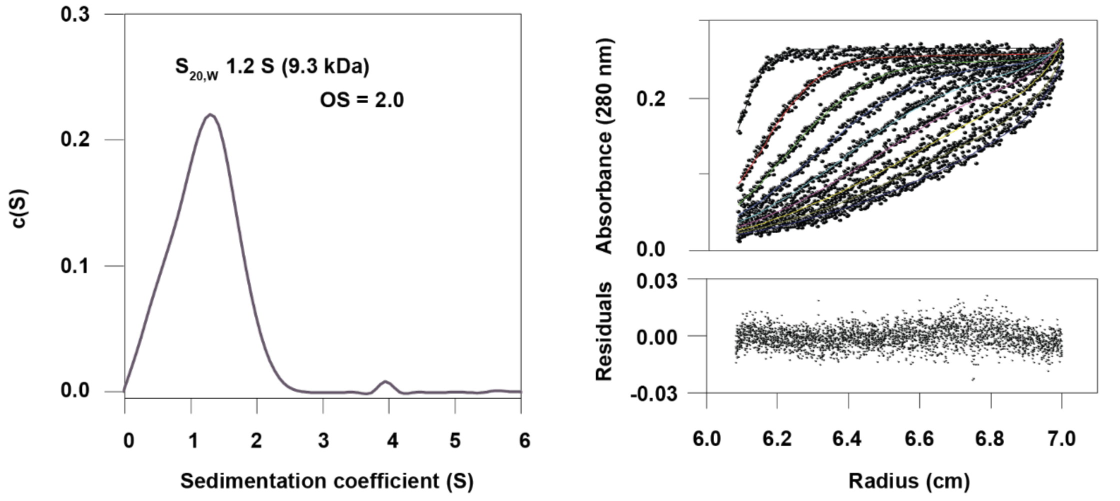

(g) SIH-15

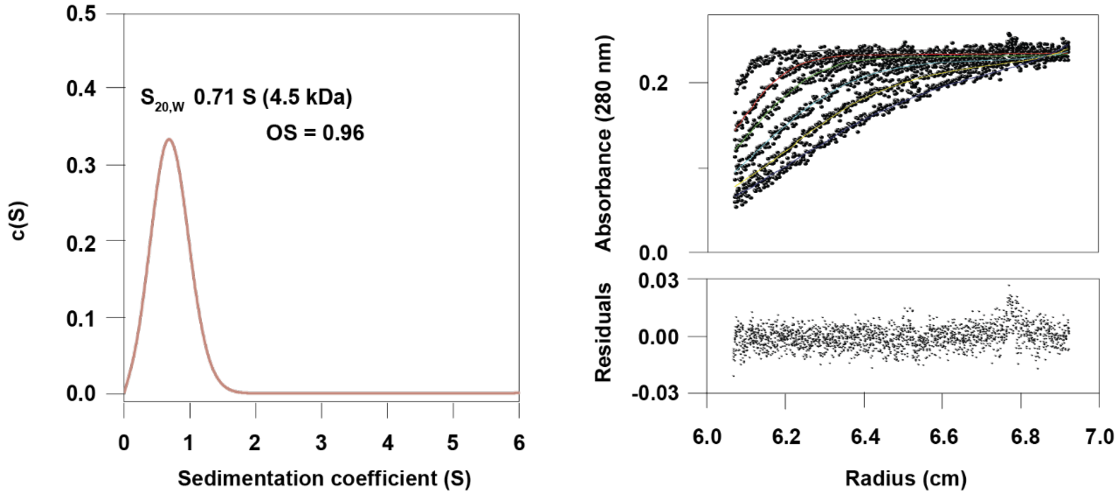

Supplementary Fig. 5. Oligomeric state of helix-hairpin peptides. Sedimentation-velocity analytical ultracentrifugation (SV-AUC) data of helix-hairpin peptides in sodium phosphate buffer (pH 7.4). *OS – oligomeric state. A subset of data from the main figures and extended data figures are replicated to present data for all the analogs for comparison and clarity.

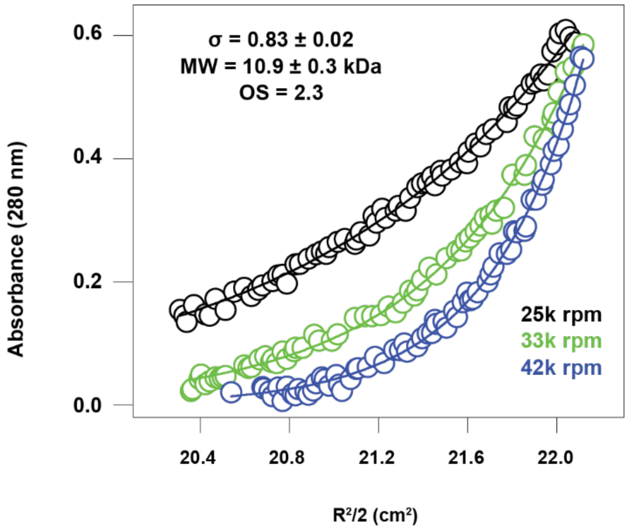

Supplementary Fig. 6. Oligomeric state of SIH-5. Sedimentation-equilibrium analytical ultracentrifugation data of SIH-5 in sodium phosphate buffer (pH 7.4). *OS – oligomeric state.

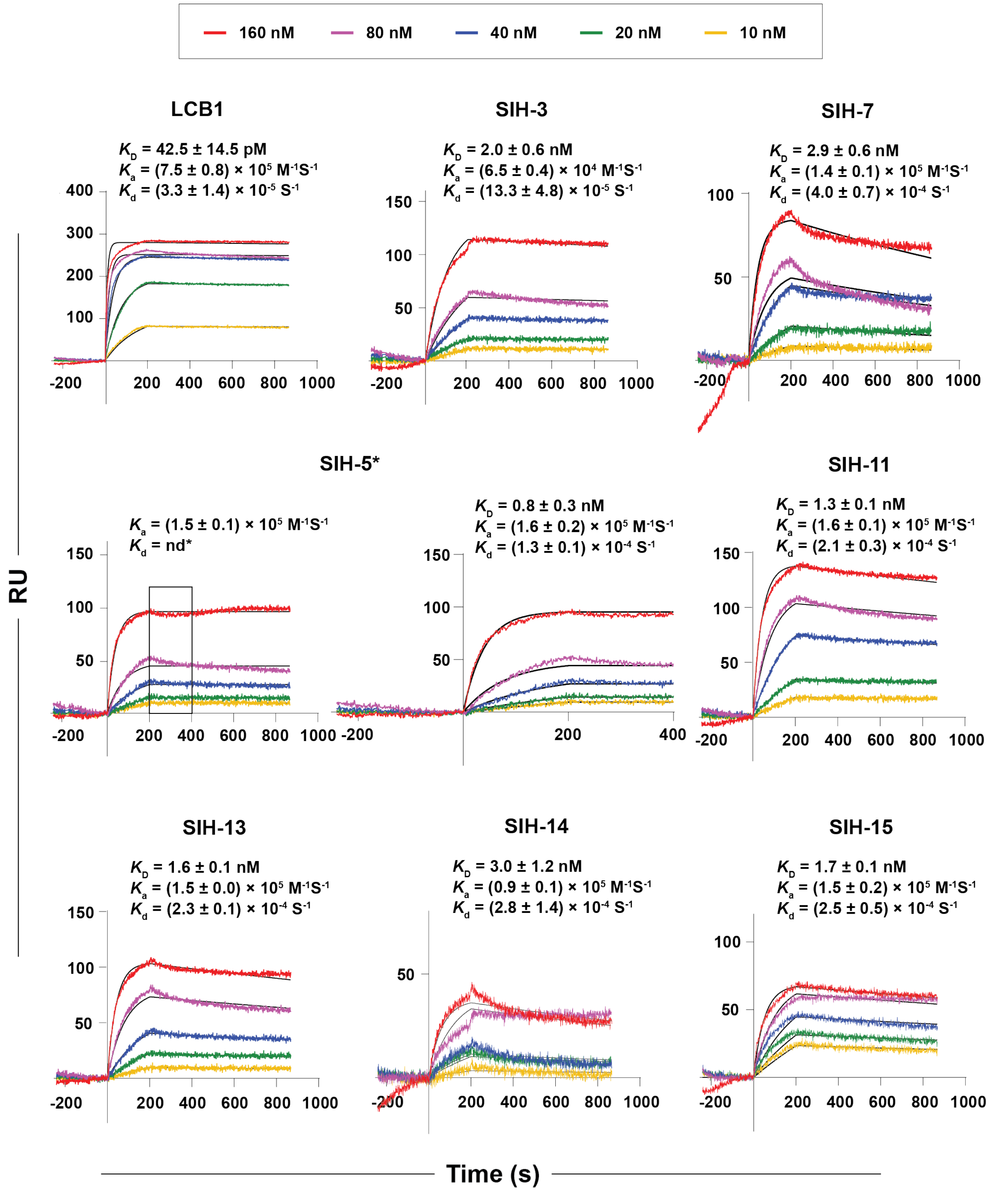

Supplementary Fig. 7. Binding affinities. The direct binding affinity of LCB1 and helix-hairpin peptides to SARS-CoV-2 RBD monitored by SPR. *The binding affinity of SIH-5 was calculated by considering 200 s (200 s to 400 s) as the dissociation time. The data represents mean values ± s.d. derived from two independent experiments. A subset of data from the main figures and extended data figures are replicated to present data for all the analogs for comparison and clarity.

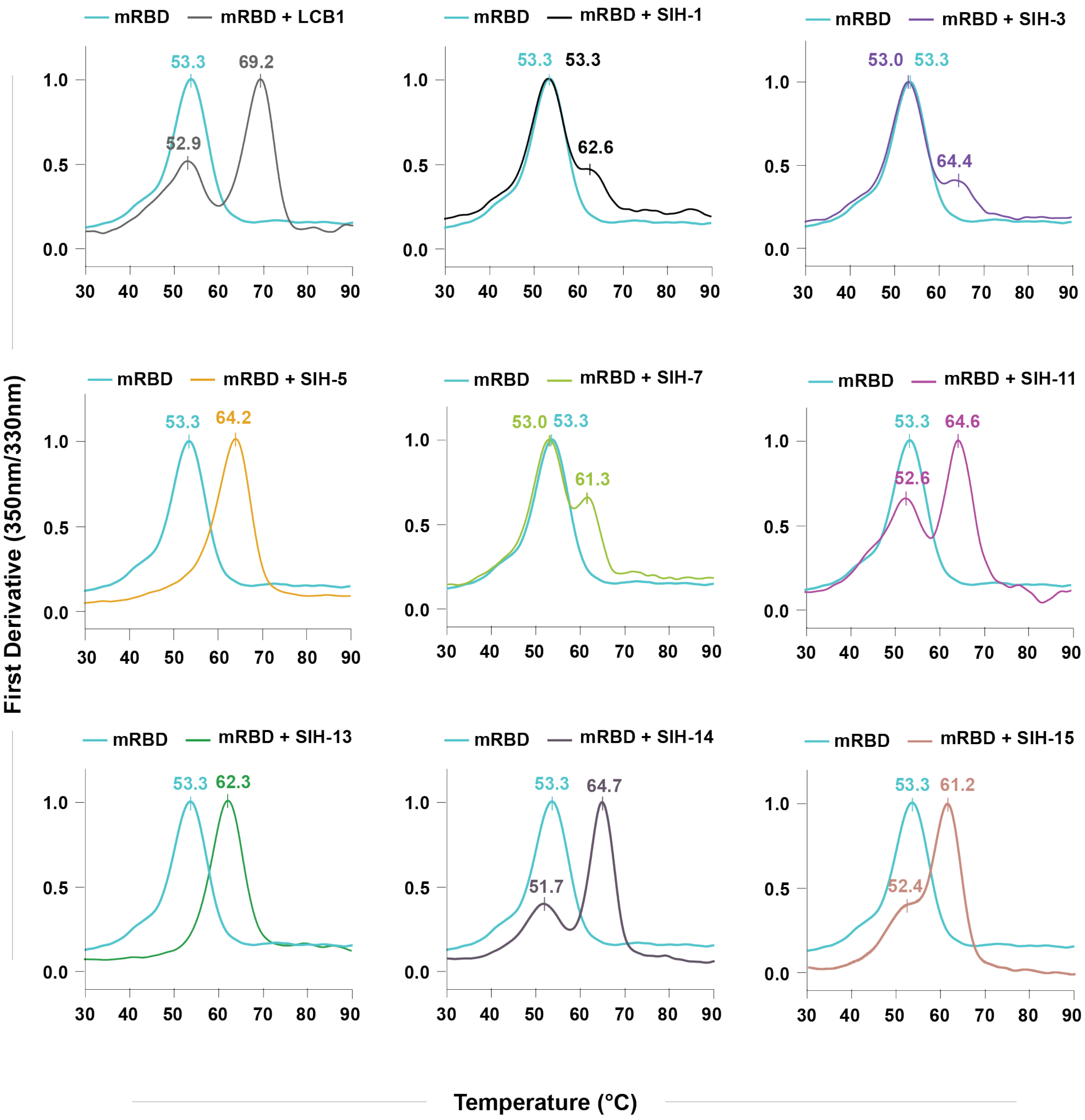

Supplementary Fig. 8. Stability of LCB1/SIH:RBD complex. The thermal stability of LCB1 and the helix-hairpin peptides in complex with SARS-CoV-2 RBD monitored by nanoDSF. The RBD was incubated with different synthetic helix-hairpin peptides (1:1 stoichiometry) in sodium phosphate buffer (pH 7.4) for at least 10 minutes at room temperature before analysis. A subset of data from the main figures and extended data figures are replicated to present data for all the analogs for comparison and clarity.

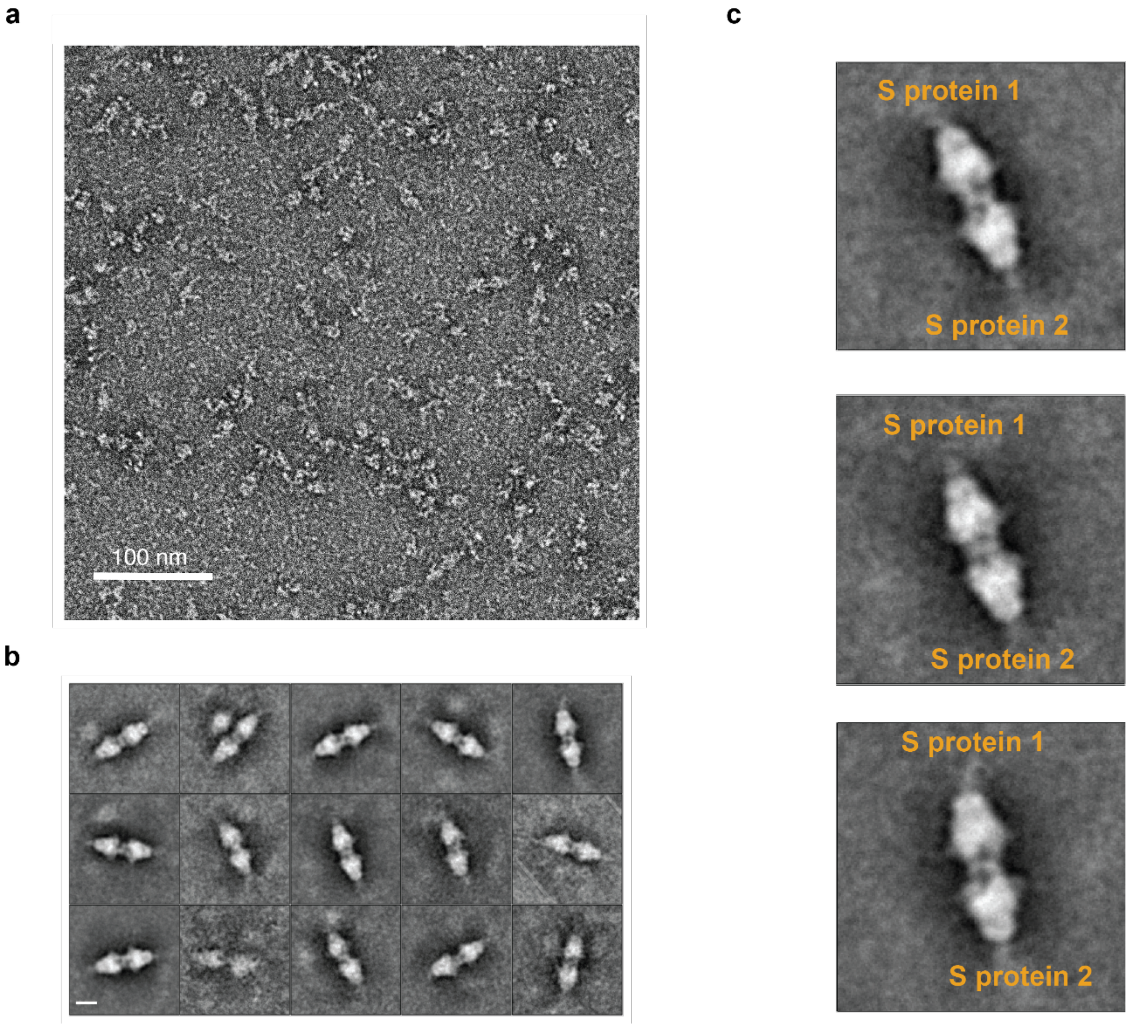

Supplementary Fig. 9. Negative staining micrograph and reference-free 2D class averages of spike (S) protein in the presence of SIH-5. a, Representative negative staining micrograph of spike protein in the presence of SIH-5 visualized using EMAN 2.1[1]. Spike protein particles are homogeneously distributed. **b**, Representative reference-free 2D class averages (extended from **Fig. 2e**) of spike protein in the presence of SIH-5 calculated using SIMPLE 2.0[2]. The scale bar represents 10 nm. Spike protein is stable in the presence of peptide and forms a dimer. **c**, Zoomed-in view of 2D class averages (selected from **Fig. 2e** and **Supplementary Fig. 9b**) of spike protein in the presence of SIH-5. In the dimeric complex, two spike proteins (S protein 1 and S protein 2) interact through the RBD region. NS EM based data analysis was performed in three sets with independent purification of Spike SIH-5 complex.

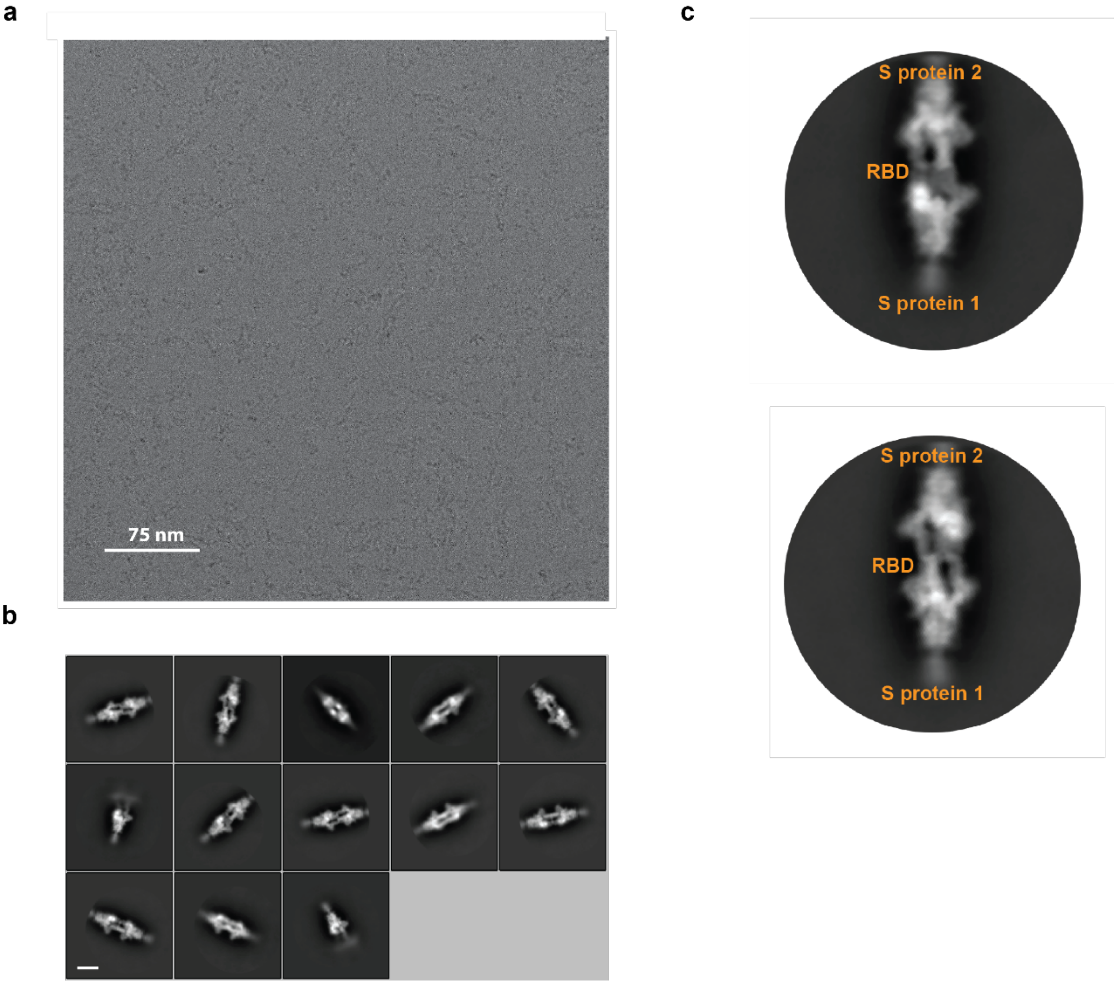

Supplementary Fig. 10. Cryo-EM micrograph and reference-free 2D class averages of spike (S) protein in the presence of SIH-5. a, Representative cryo-EM micrograph (one out of two independent experiments) of spike protein in the presence of SIH-5 after beam-induced motion correction using MotionCor2 software[3]. Spike protein particles are homogeneously distributed. **b**, Representative reference-free 2D class averages (extended from **Fig. 2f**) of spike protein in the presence of SIH-5. The scale bar represents 10 nm. Spike protein is stable in the presence of peptide and forms the dimeric complex. **c**, Zoomed-in view of 2D class averages (selected from **Fig. 2f** and **Supplementary Fig. 10b**) show the dimeric spike protein with two Spike protein trimers interacting through the RBDs.

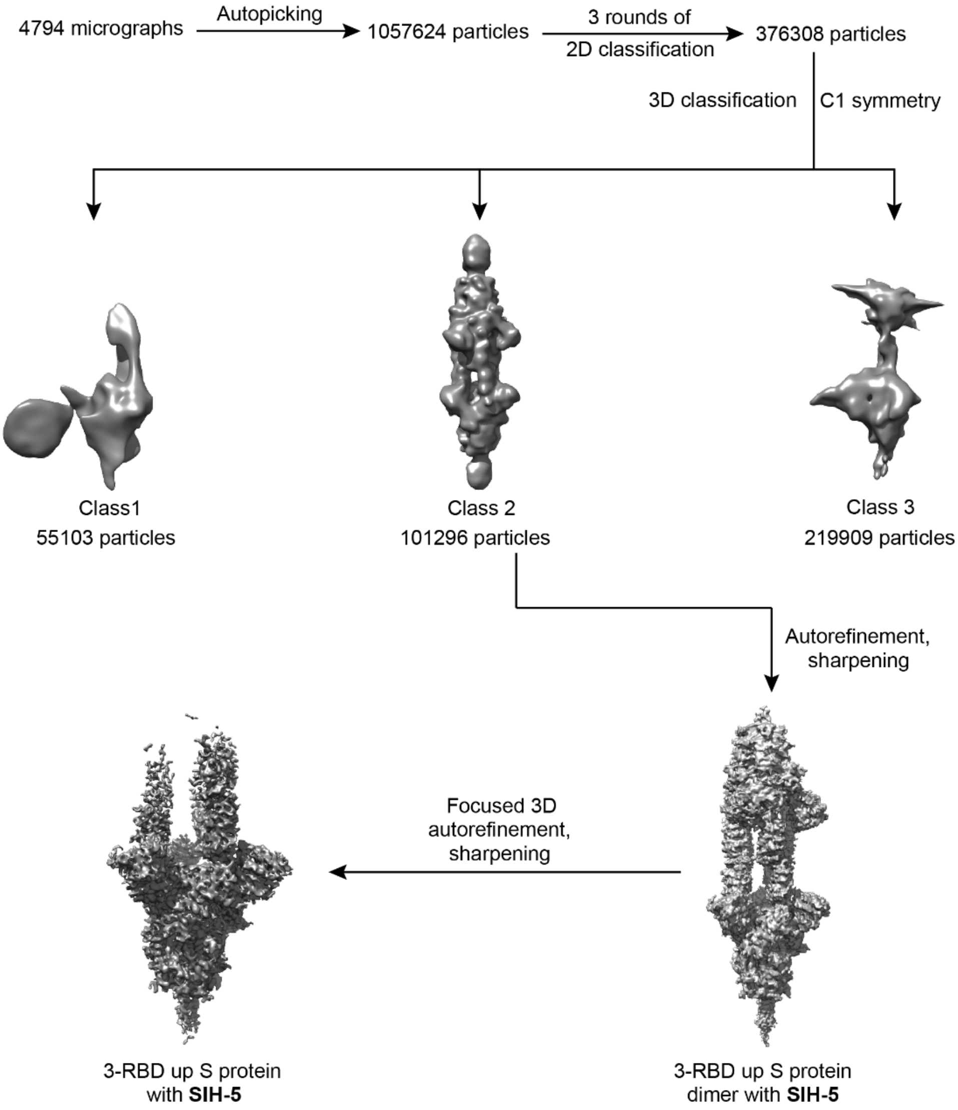

Supplementary Fig. 11. Pipeline of data processing using single-particle cryo-EM and 3D classification of spike (S) protein in the presence of SIH-5: Cryo-EM data processing workflow and structure determination of spike protein in the presence of SIH-5 done in RELION 3.1[4]. The detailed method is provided in the method section. Class 2 is the dimeric complex of the spike protein. All 3D visualization was done in chimera[5] and chimeraX[6,7].

a

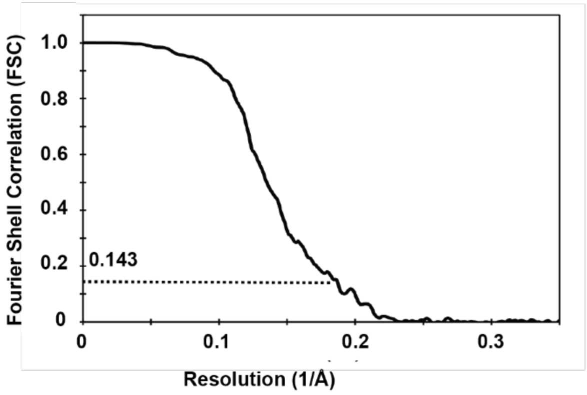

b

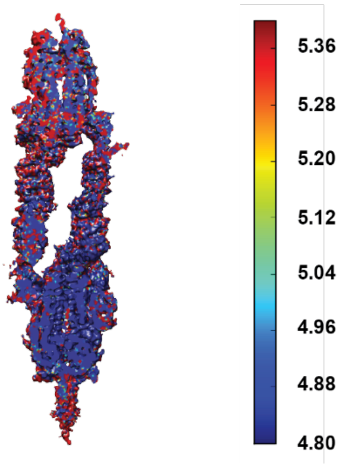

Supplementary Fig. 12. FSC calculation and local resolution estimation for dimeric 3-RBD up spike protein. a, Gold standard Fourier Shell Correlation (FSC) calculation at 0.143 for dimeric 3-RBD up spike protein in the presence of SIH-5. FSC curve shows a resolution of 5.4 Å. **b**, Local resolution calculation for dimeric 3-RBD up spike protein.

a b

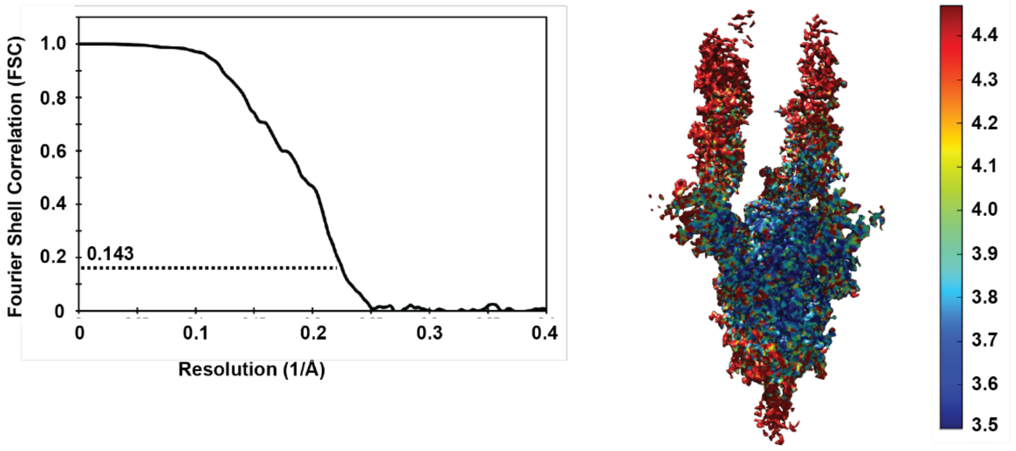

Supplementary Fig. 13. FSC calculation and local resolution estimation for single 3-RBD up spike protein after masked auto-refinement. a, Gold standard Fourier Shell Correlation (FSC) calculation at 0.143 for single 3-RBD up spike protein after masked auto-refinement. FSC curve shows a resolution of 4.47 Å. **b**, Local resolution calculation for high-resolution single 3-RBD up spike protein after masked auto-refinement in ResMap [8].

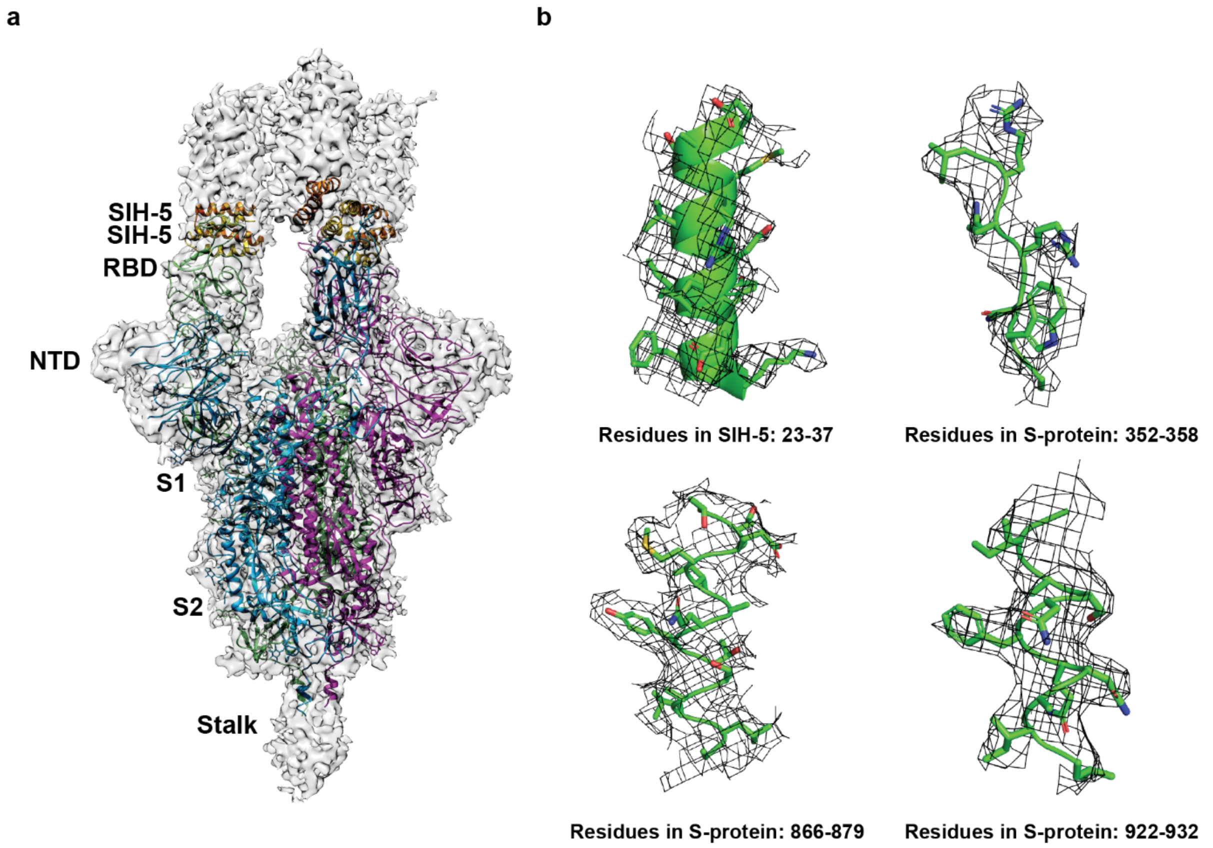

Supplementary Fig. 14. Atomic model fitting in the cryo-EM map of single spike (S) protein with dimeric SIH-5 after masked auto-refinement. a, Transparent representation of the cryo-EM 3D model of single 3-RBD up spike protein and dimeric SIH-5 fitted with the calculated atomic model was built in phenix and coot. The image (**Fig. 3b**) is replicated for comparison and clarity. **b**, Side chain fitting at different regions of the spike protein shows the proper fitting of amino acid residues in the cryo-EM map. The electron density map is contoured at 4.28σ.

a) Pseudovirus (control)

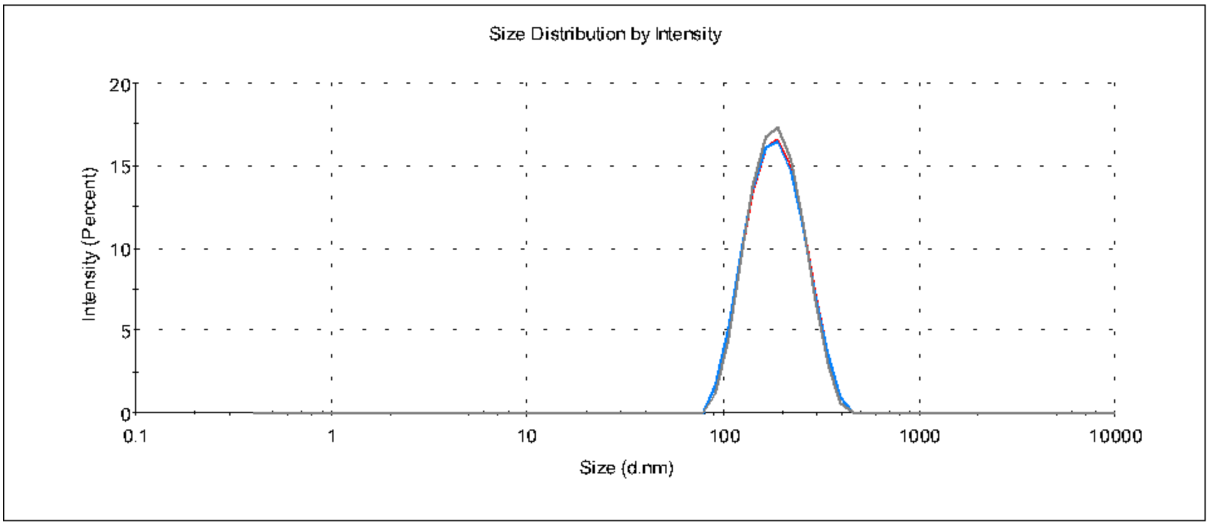

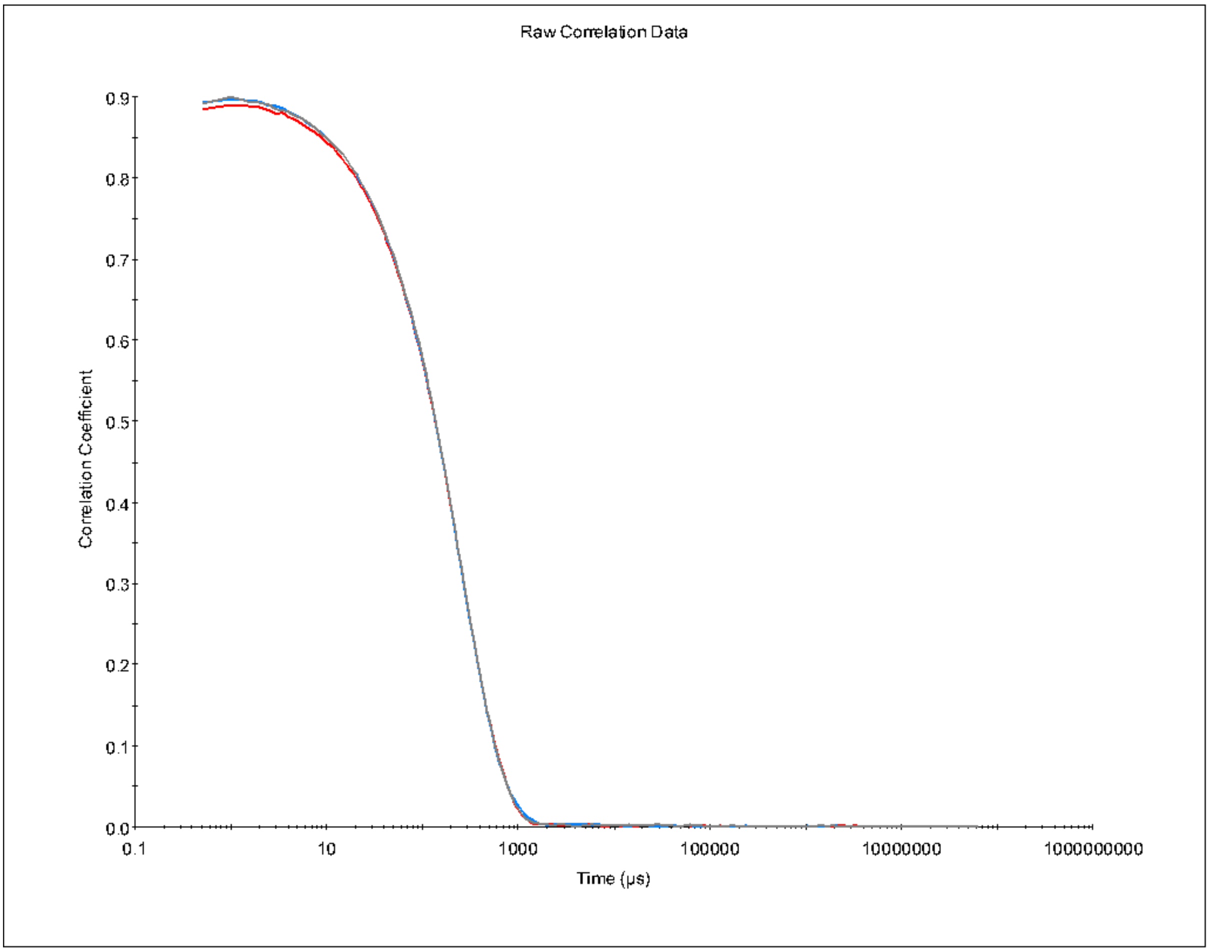

b) Pseudovirus + LCB1

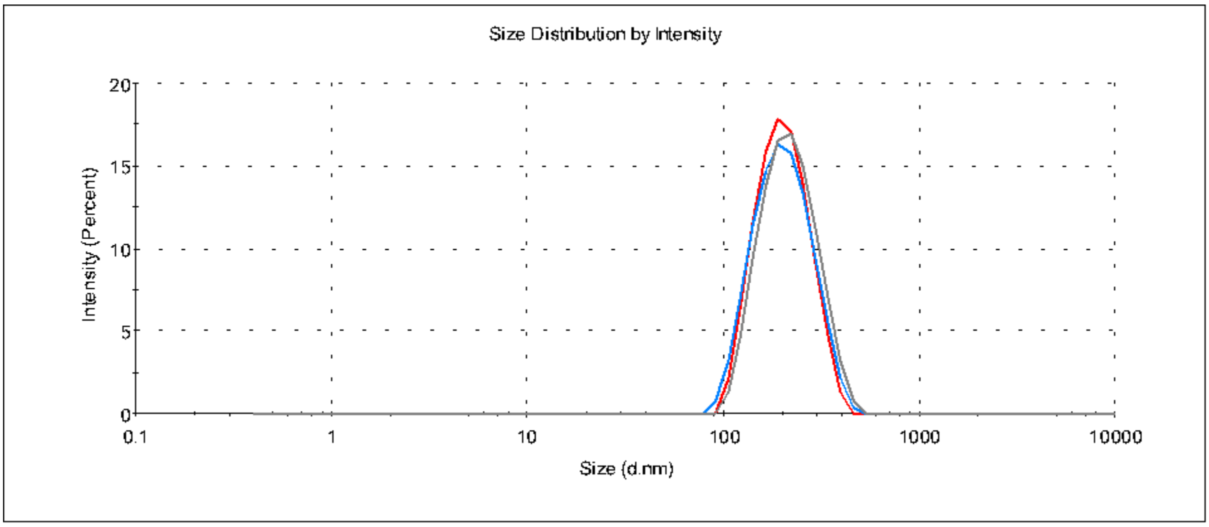

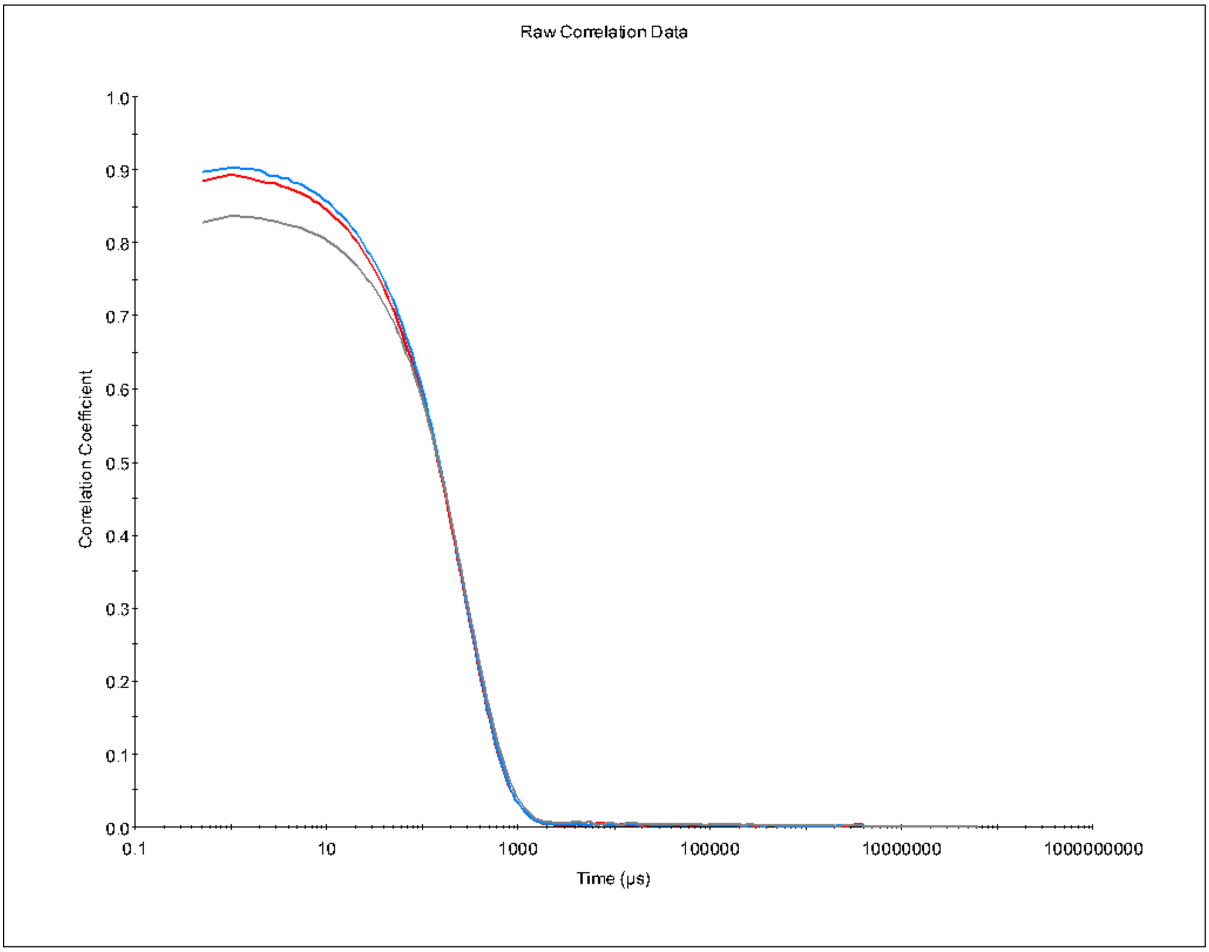

c) Pseudovirus + SIH-5

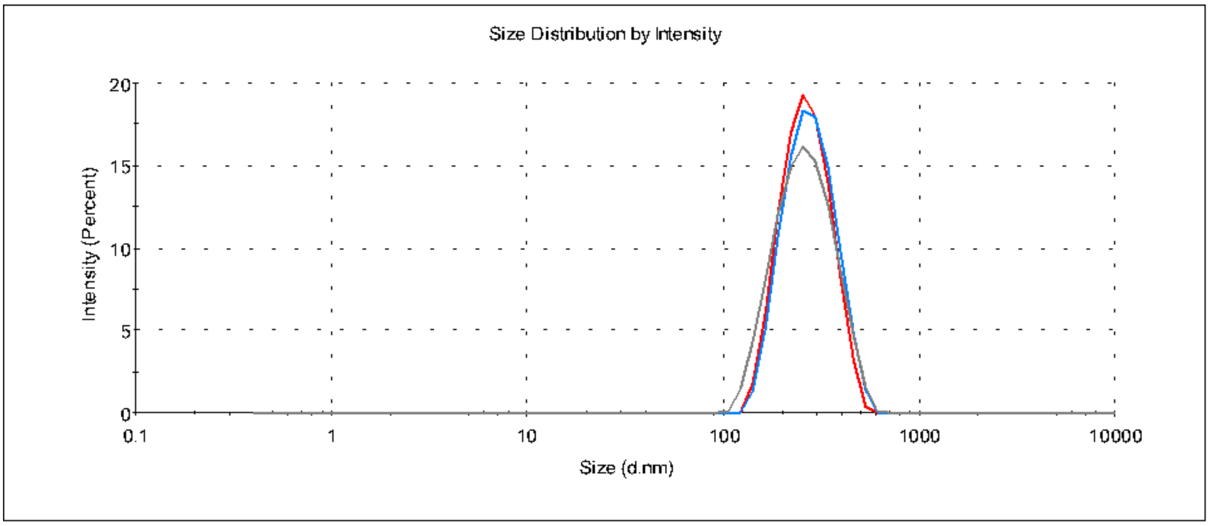

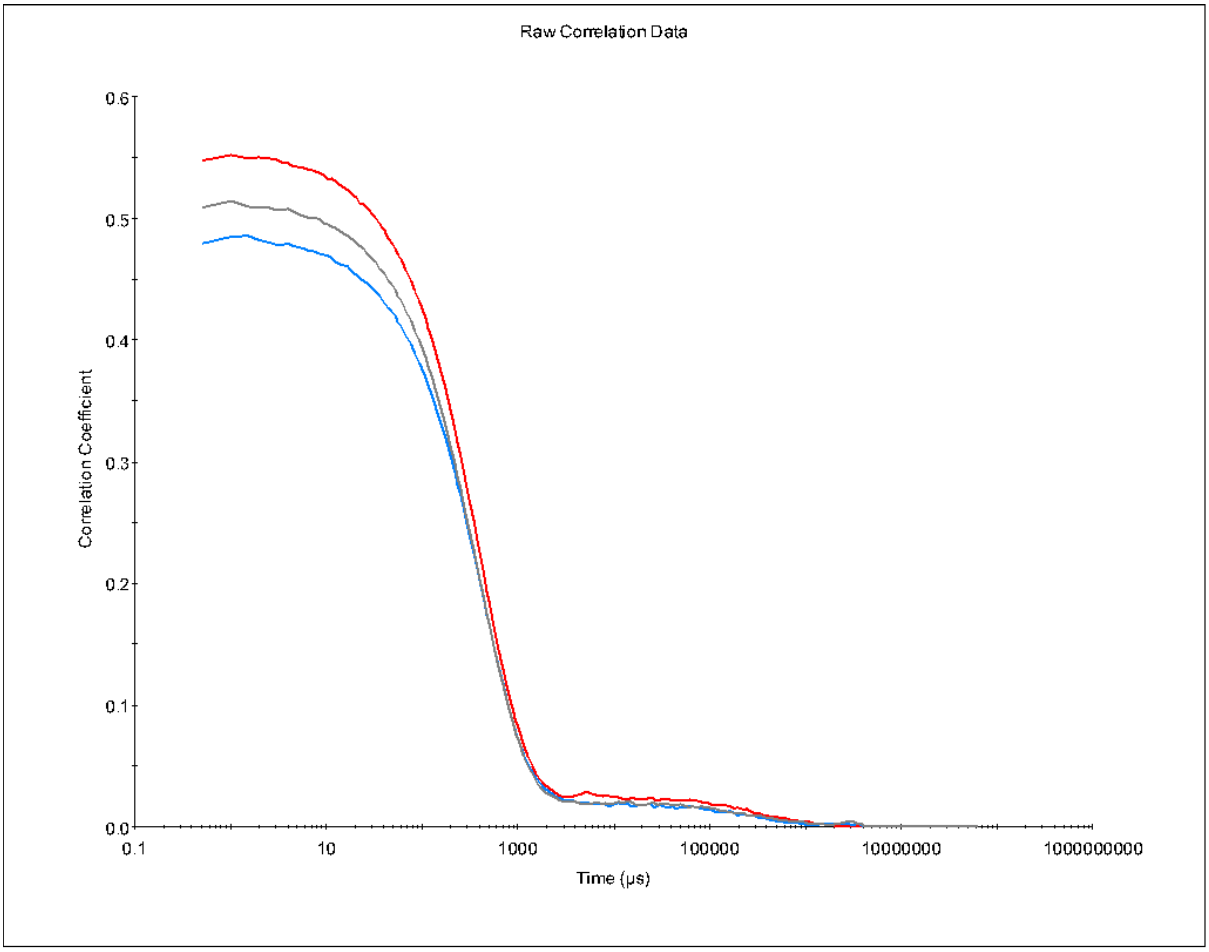

d) Pseudovirus + SIH-11

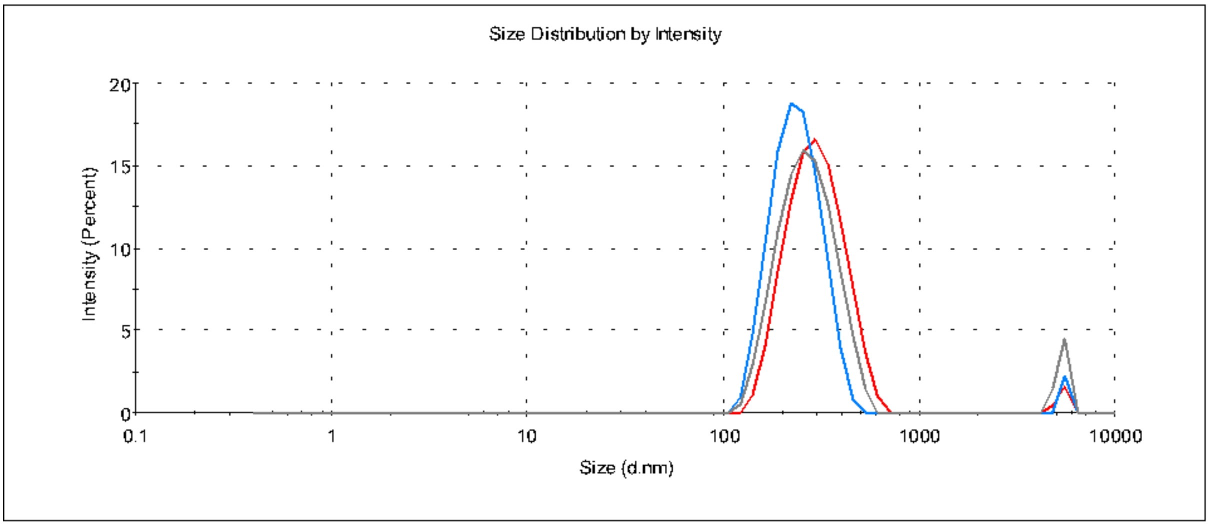

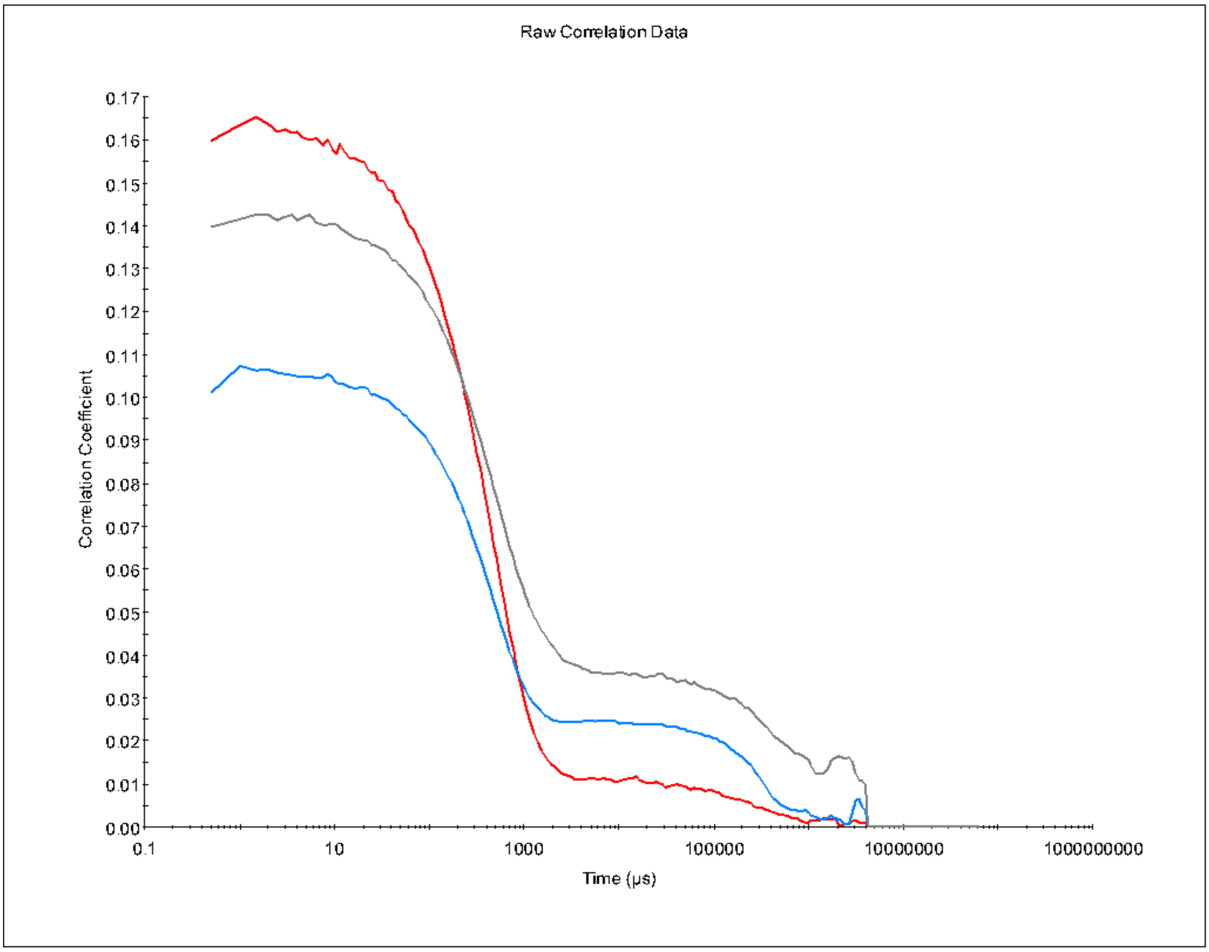

Supplementary Fig. 15. Virus particle aggregation in the presence of helix-hairpin peptides. DLS intensity distribution (upper panel) and the correlation curves (lower panel) for pseudovirus (a), pseudovirus + LCB1 (b), pseudovirus + SIH-5 (c), and pseudovirus + SIH-11 (d). The data derived from three independent experiments (grey, blue, and red lines) are overlayed.

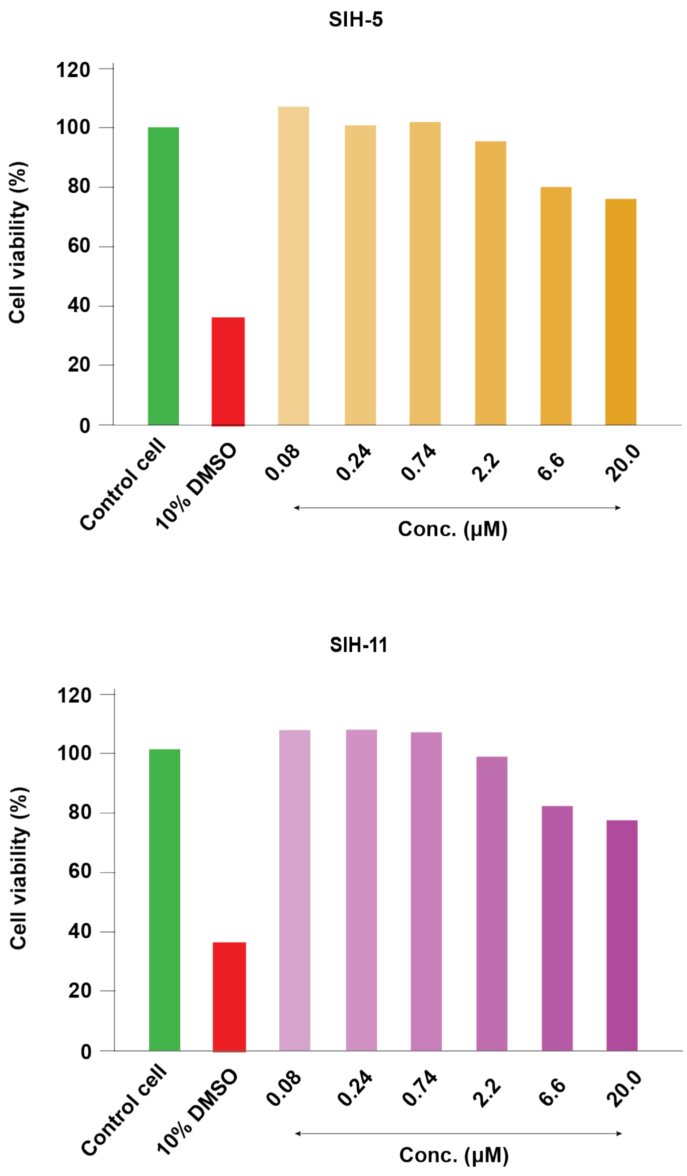

Supplementary Fig. 16. Cytotoxicity of SIH-5 and SIH-11. 48 h cell viability (determined by MTT assay) of Vero-E6 cells (monkey kidney) incubated with different concentrations of SIH-5 or SIH-11. Mean data from two independent experiments are plotted.

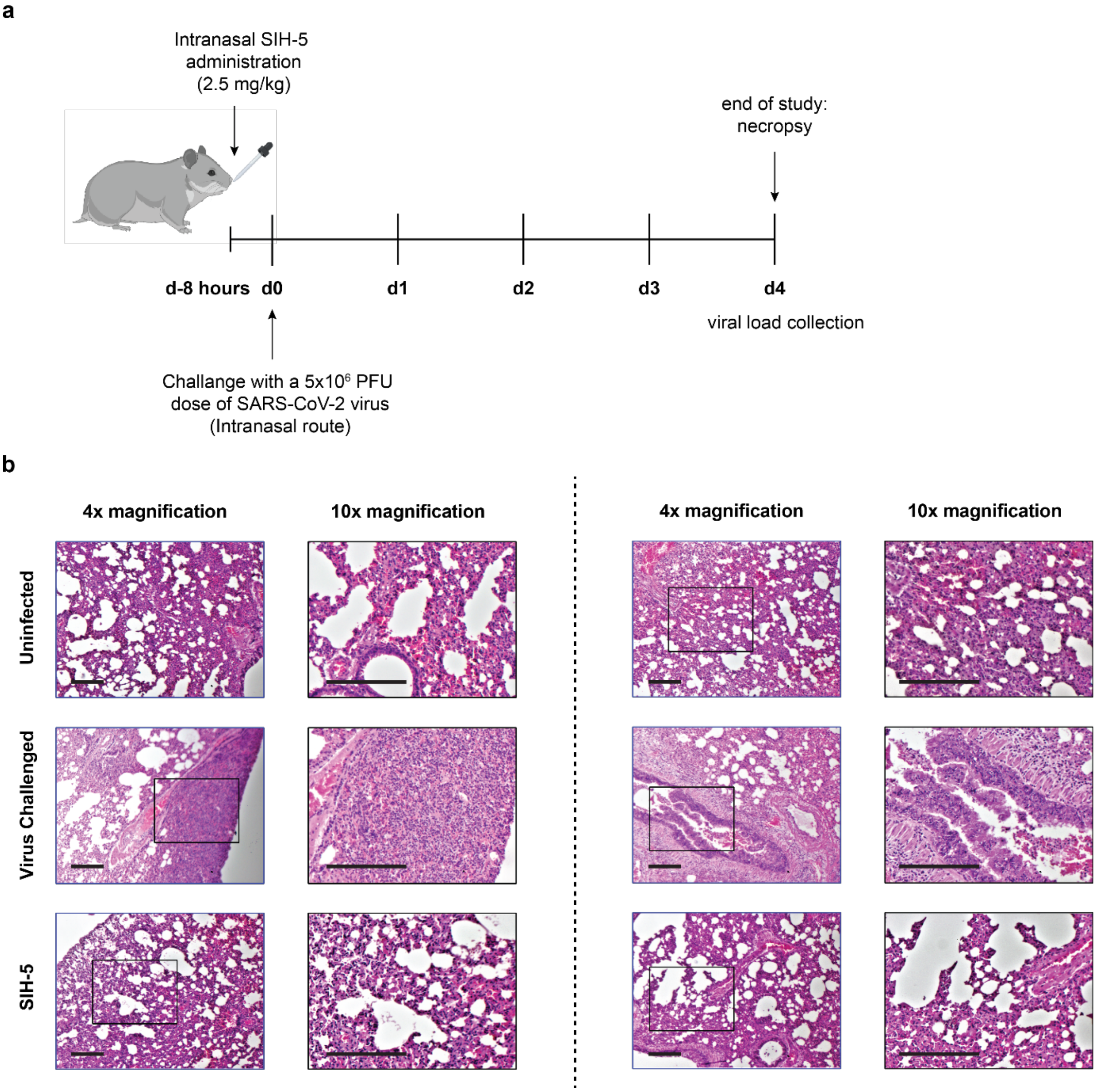

Supplementary Fig. 17. In vivo prophylactic activity of SIH-5 against SARS-CoV-2 in Syrian hamsters. a, Overview of experimental design. The hamsters were divided into three groups: (i) control-PBS treated (n=3), (ii) challenged with 5x10^{6} PFU dose of SARS-CoV-2 (n=5), and (iii) SIH-5 treated (day 0 at -8 h) and challenged (n=5). **b**, Representative images of histopathology of lungs from PBS-treated, virus-challenged, and SIH-5 peptide treated at 4 dpi. The scale bars represent 50 µm.

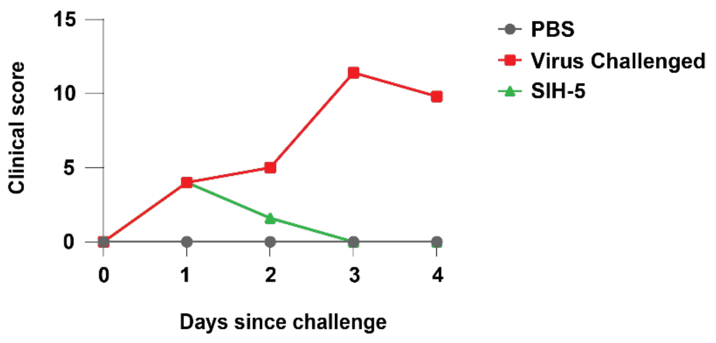

Supplementary Fig. 18. Comparison of average clinical score in the three different groups. The 14-point system used for the average clinical score is discussed in Supplementary Table 4.

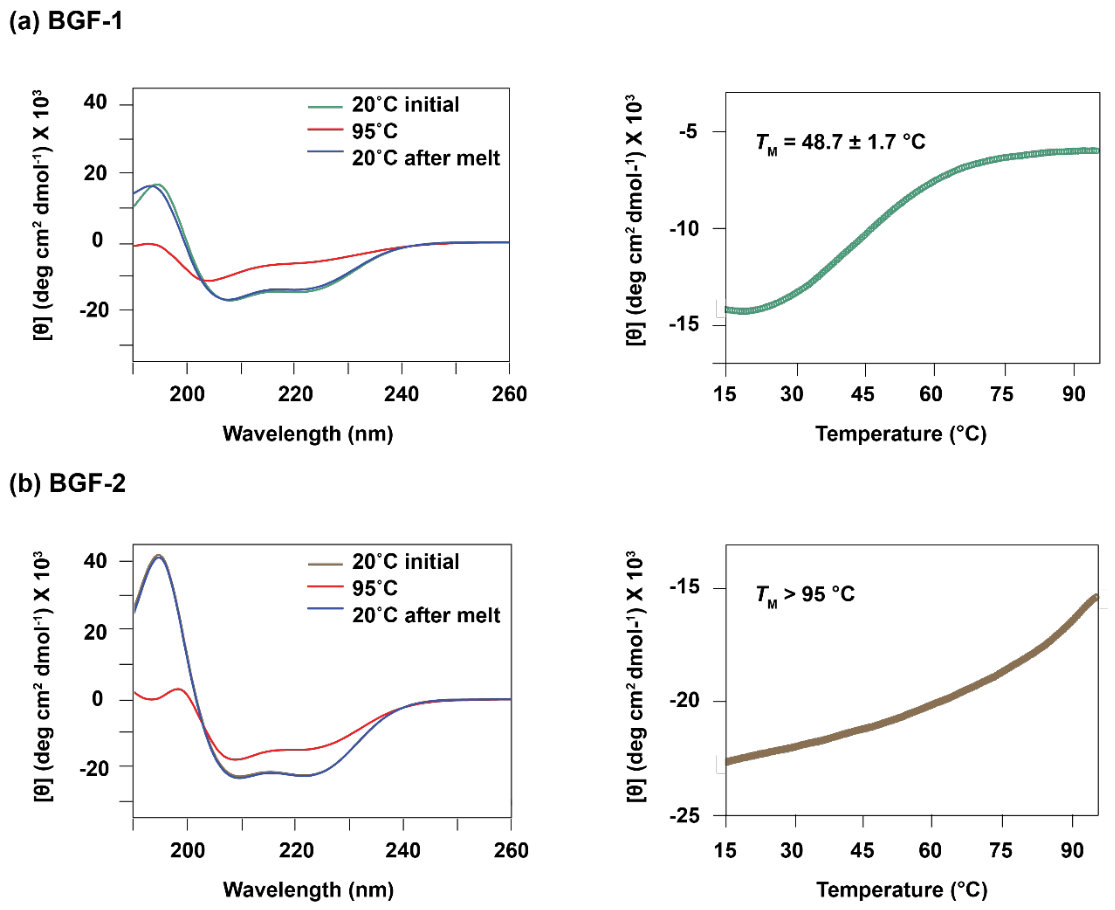

Supplementary Fig. 19. Thermal stability and reversibility of BGF variants. Circular dichroism (CD) spectra of the synthetic peptides at different temperatures (left panel). The structural reversibility after removal of thermal stress is observed by comparing the CD spectrum at 20 °C and 20 °C after melt. CD thermal unfolding was monitored at 222 nm (right panel). Data were fit to a two-state unfolding model to obtain the melting temperature. The thermal denaturation data (**Fig. 6c**) are replicated for comparison.

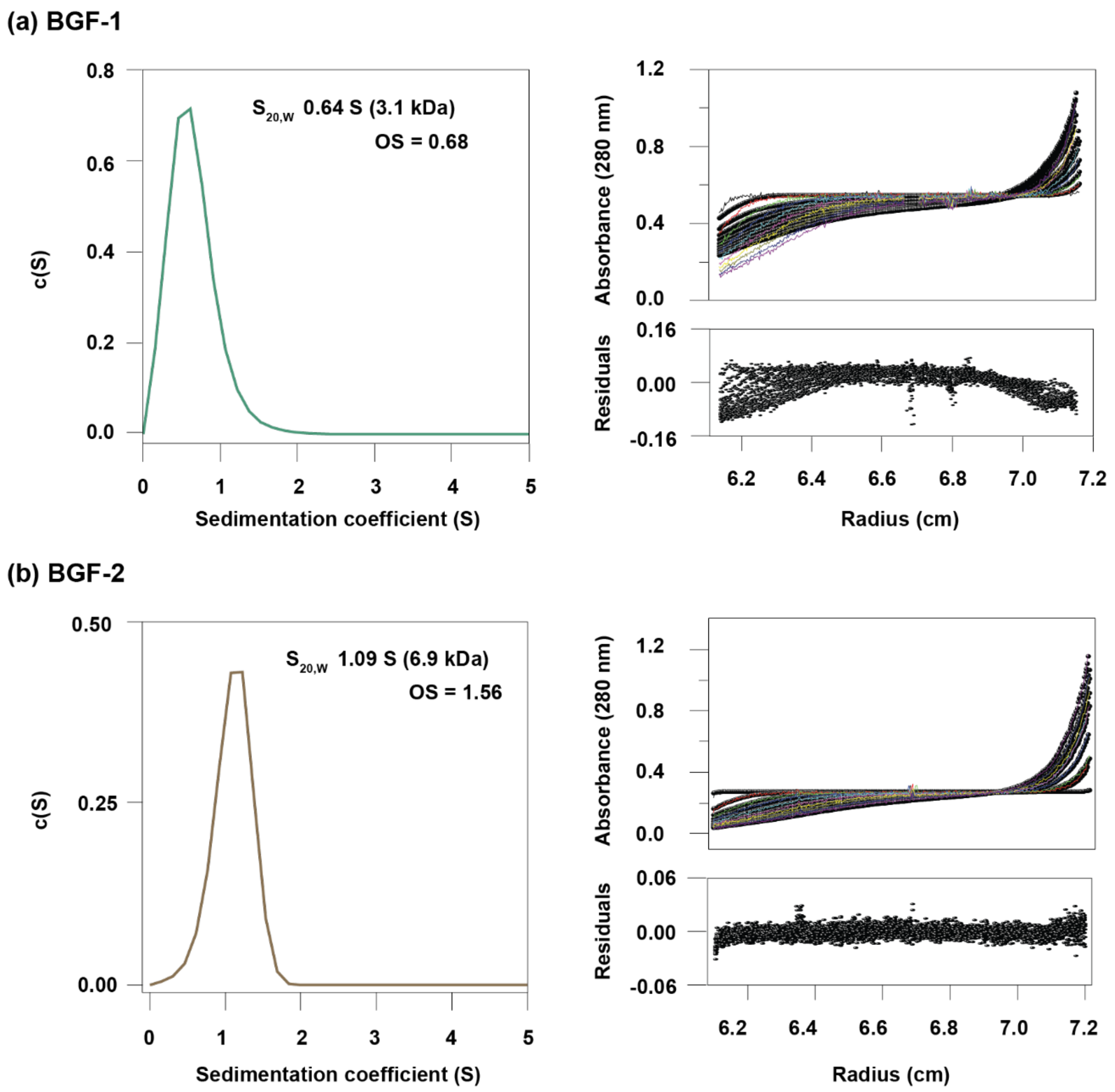

Supplementary Fig. 20. Oligomeric state of helix-hairpin peptides. Sedimentation-velocity analytical ultracentrifugation data of BGF-1 and BGF-2 in sodium phosphate buffer (pH 7.4). *OS – oligomeric state.

Supplementary Table 1. Peptide concentration in saturated solution. The solubility was assessed in sodium phosphate buffer, pH 7.4.

**Peptide**	**Concentration (µM)**
SIH-1	17
SIH-2	32
SIH-3	95
SIH-4	220
SIH-5	>700

Supplementary Table 2. Cryo-EM data collection, refinement and validation statistics.

	Single trimeric spike protein with SIH-5 (EMDB-33042) (PDB 7X7N)	Dimeric Spike protein complex with SIH-5 (EMDB-32388)
**Data collection and processing**		
Magnification	42,200	42,200
Voltage (kV)	200	200
Electron exposure (e–/Å^2)	80	80
Defocus range (μm)	-0.75 to -2.5	-0.75 to -2.5
Pixel size (Å)	1.17	1.17
Symmetry imposed	C1	C1
Initial particle images (no.)	1,057,624	1,057,624
Final particle images (no.)	101296	101296
Map resolution (Å)	4.47	5.4
FSC threshold	0.143	0.143
Map resolution range (Å)	3.5-4.47	4.8-5.4
**Refinement**		
Initial model used (PDB code)	7KMS	-
Model resolution (Å)	4.47	-
FSC threshold	0.143	-
Model resolution range (Å)	3.5-4.47	-
Map sharpening *B* factor (Å^2)	-120	-
Model composition		
Non-hydrogen atoms	26493	-
Protein residues	3314	-
Ligands	-	-
B factors (Å^2)		
Protein	-	-
Ligand	-	-
R.m.s. deviations		
Bond lengths (Å)	0.013	-
Bond angles (°)	1.032	-
Validation		
MolProbity score	2.68	-
Clashscore	28	-
Poor rotamers (%)	0.52	-
Ramachandran plot		
Favored (%)	80.27	-
Allowed (%)	19.61	-
Disallowed (%)	0.12	-

Supplementary Table 3. Aggregation assessment for pseudovirus (control) and pseudovirus incubated with LCB1, SIH-5, and SIH-11. The average particle size and polydispersity index ± s.d. are obtained from three independent DLS measurements.

Sample	Size (diameter nm ± SD)	PD Index
Pseudovirus (control)	155.5 ± 0.5	0.24
Pseudovirus + LCB1	175.8 ± 7.5	0.21
Pseudovirus + SIH-5	271.2 ± 4.2	0.27
Pseudovirus + SIH-11	335.2 ± 19.2	0.31

Supplementary Table 4. Mean clinical scores for the hamsters. The scoring system for evaluating clinical symptoms is: lethargy (1 point), rough coat (1 point), sneezing (1 point), mucus discharge (1 point), huddling in the corner (1 point), ears laid back (1 point), half-closed eyes/watery eyes (1 point), head tilt (1 point), moderate dyspnoea (1 point), hunched back (1 point), weight loss 3-5% (1 point), weight loss 6-10% (2 points), weight loss 11-20% (3 points), and shaking or shivering (1 point). n=3 for Unchallenged, n=5 for virus-challenged, and SIH-5 treated.

No.	Clinical Signs	Unchallenged, PBS- treated					Challenged					SIH 5- treated (day 0 at -8 h) and challenged				
		Day					Day					Day				
		0	1	2	3	4	0	1	2	3	4	0	1	2	3	4
1	Lethargy and inactivity	0	0	0	0	0	0	1	1	1	1	0	1	0.2	0	0
2	Rough coat	0	0	0	0	0	0	1	1	1	1	0	1	0.2	0	0
3	Sneezing	0	0	0	0	0	0	0	0	1	1	0	0	0	0	0
4	Mucus discharge (nose/eyes)	0	0	0	0	0	0	0	0	1	0	0	0	0	0	0
5	Huddling in the corner	0	0	0	0	0	0	1	1	1	1	0	1	1	0	0
6	Ears laid back	0	0	0	0	0	0	0	0	1	1	0	0.4	0.2	0	0
7	Half closed eyes/watery eyes	0	0	0	0	0	0	0	0	1	1	0	0	0	0	0
8	Head tilt	0	0	0	0	0	0	0	0	1	1	0	0.2	0	0	0
9	Moderate dyspnoea	0	0	0	0	0	0	0	0	0.4	0.4	0	0	0	0	0
10	Hunched back	0	0	0	0	0	0	0	0	1	1	0	0	0	0	0
11	Weight loss 3-5%	0	0	0	0	0	0	1	0.8	0.8	0.2	0	0.4	0	0	0
12	Weight loss 6-10%	0	0	0	0	0	0	0	0	0	0	0	0	0	0	0
13	Weight loss 11-20%	0	0	0	0	0	0	0	0.6	0.6	0.6	0	0	0	0	0
14	Shaking or shivering	0	0	0	0	0	0	0	0.6	0.6	0.6	0	0	0	0	0
Total score		0	0	0	0	0	0	4	5	11.4	9.8	0	4	1.6	0	0

Supplementary Note

Analytical HPLC chromatograms and ESI-MS spectra of purified peptides

(a) LCB1

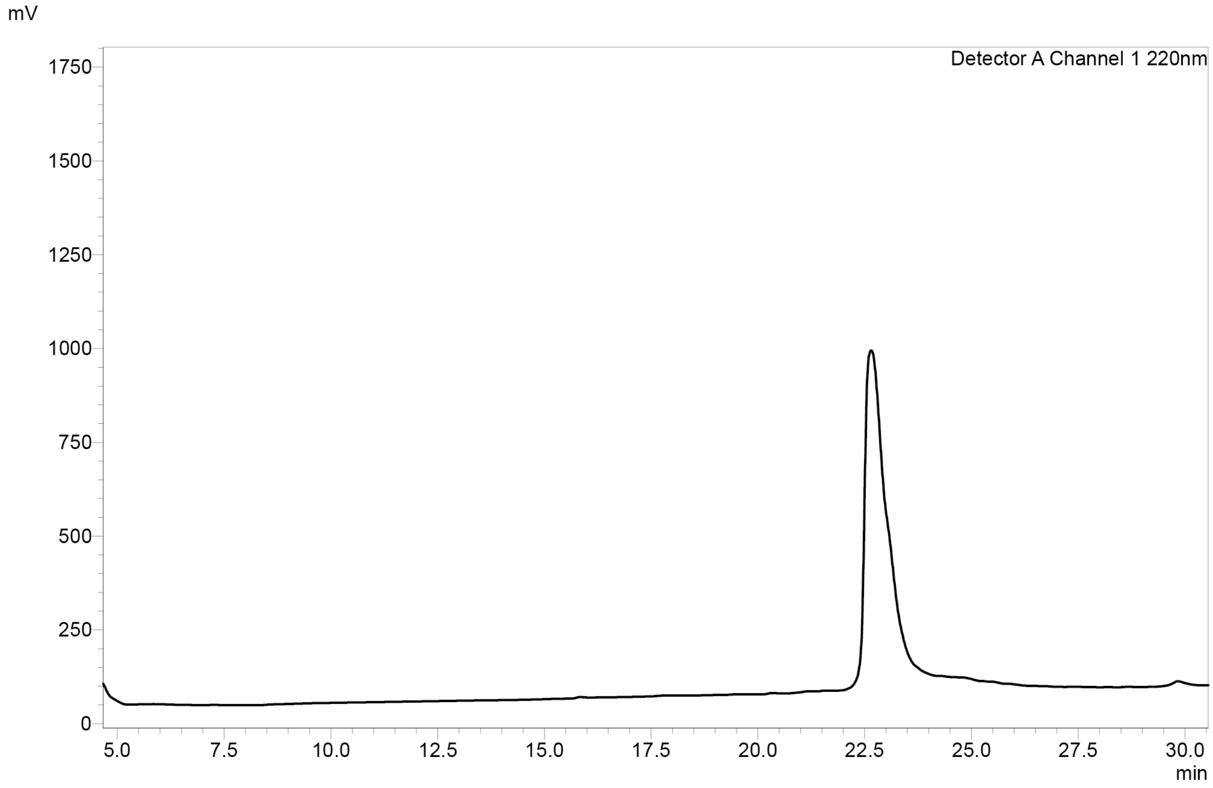

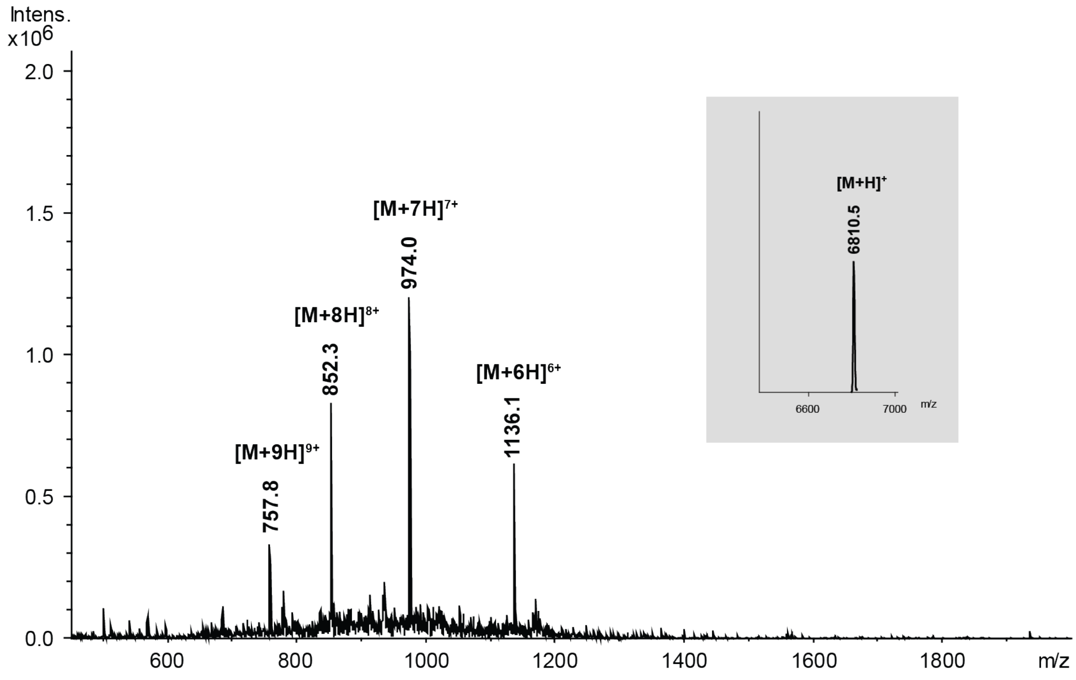

(b) SIH-1

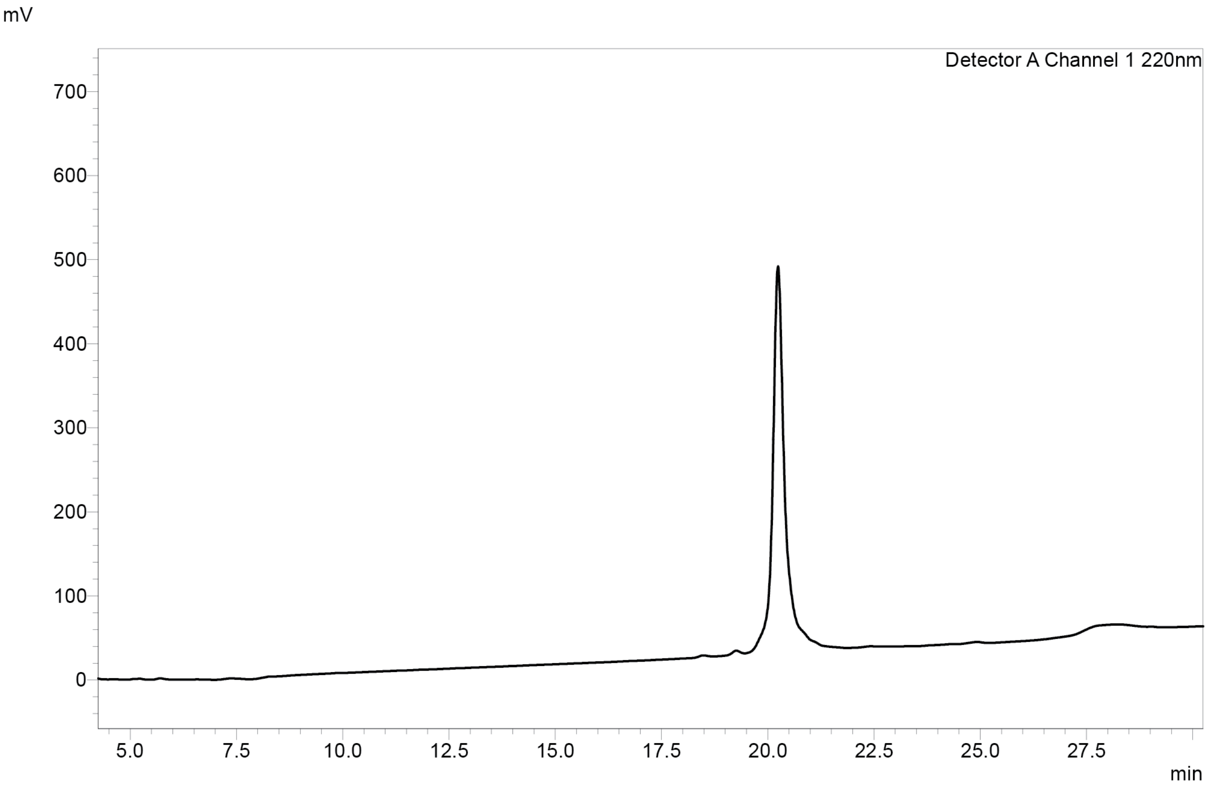
mV
Detector A Channel 1 220nm
700
600
500
400
300
200
100
0
5.0
7.5
10.0
12.5
15.0
17.5
20.0
22.5
25.0
27.5
min

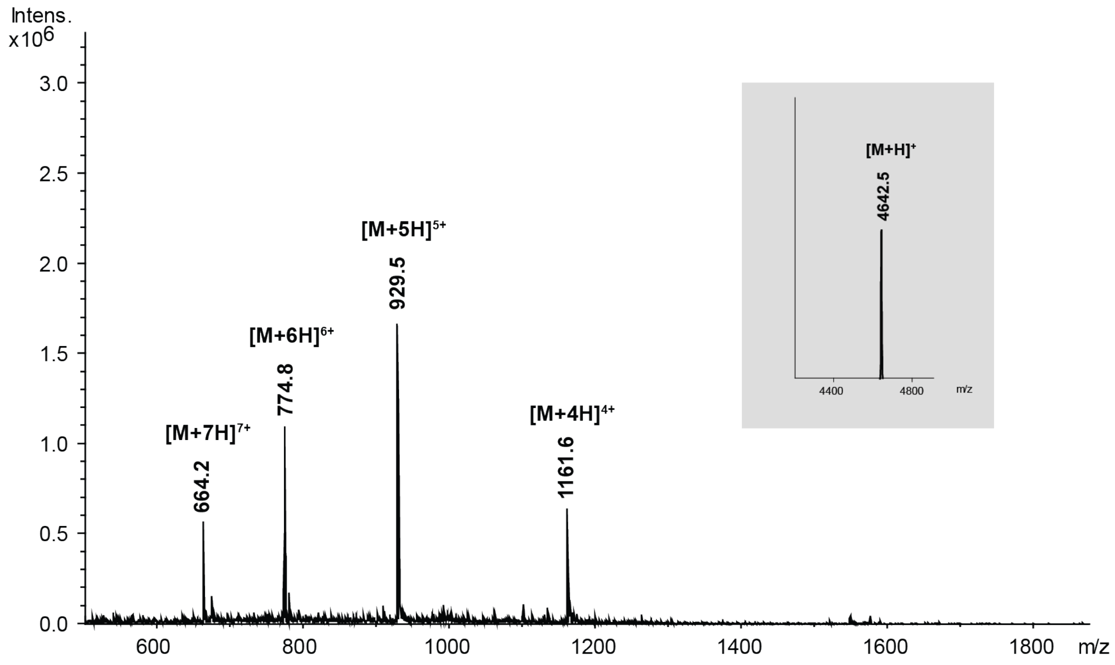
Intens.
x10^6
3.0
2.5
2.0
1.5
1.0
0.5
0.0
[M+7H]7+
664.2
[M+6H]6+
774.8
[M+5H]5+
929.5
[M+4H]4+
1161.6
[M+H]+
4642.5
4400
4800
m/z
600
800
1000
1200
1400
1600
1800
m/z

(c) SIH-2

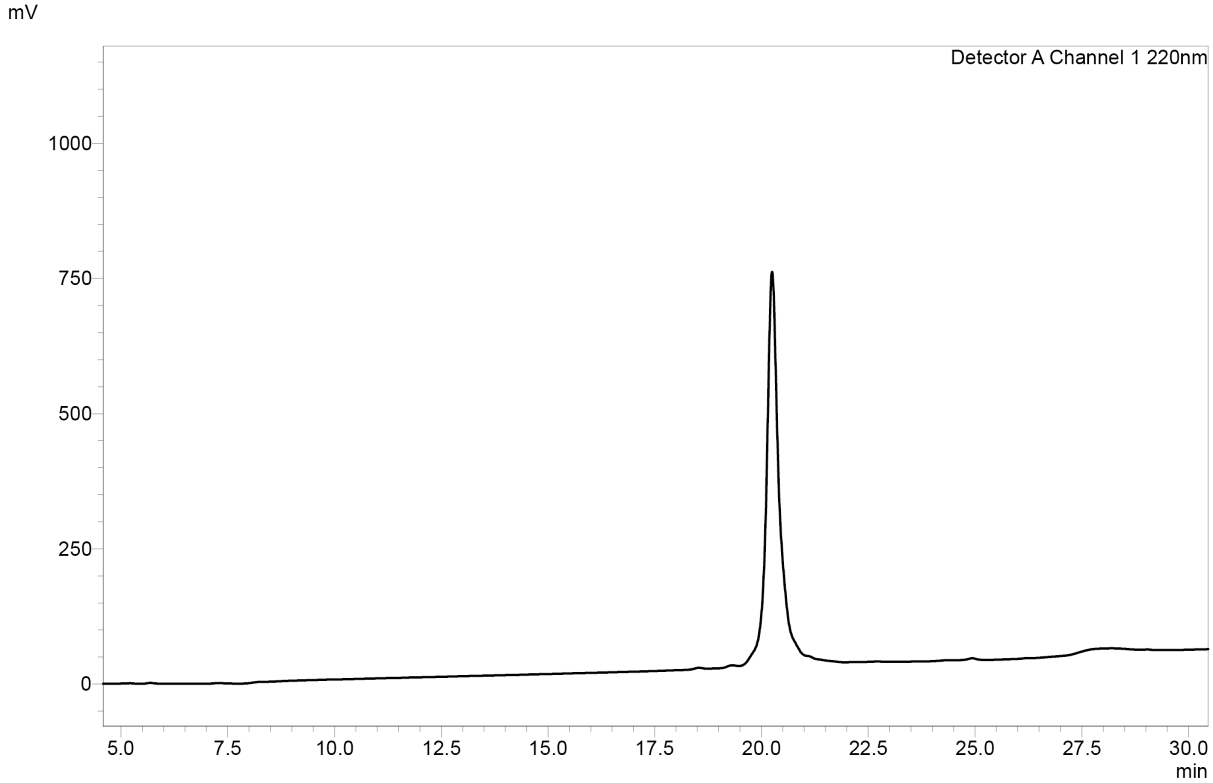

mV
Detector A Channel 1 220nm
1000
750
500
250
0
5.0
7.5
10.0
12.5
15.0
17.5
20.0
22.5
25.0
27.5
30.0
min

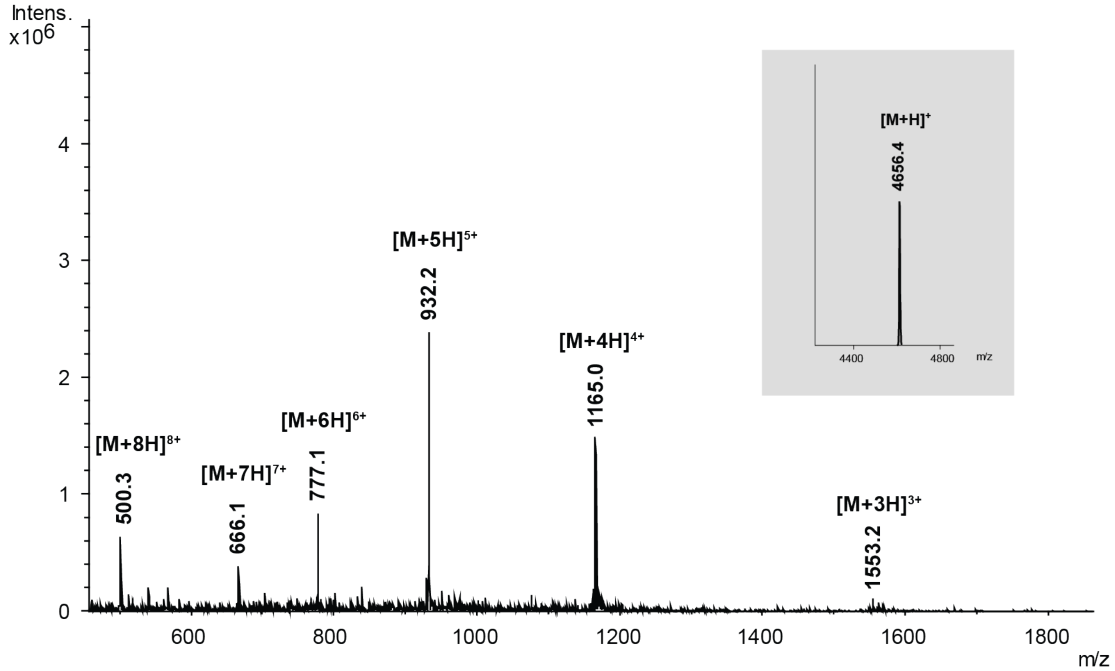

Intens.
x10^6
[M+H]+
4656.4
4400
4800
m/z
[M+5H]5+
932.2
[M+4H]4+
1165.0
[M+6H]6+
777.1
[M+8H]8+
500.3
[M+7H]7+
666.1
[M+3H]3+
1553.2
600
800
1000
1200
1400
1600
1800
m/z

(d) SIH-3

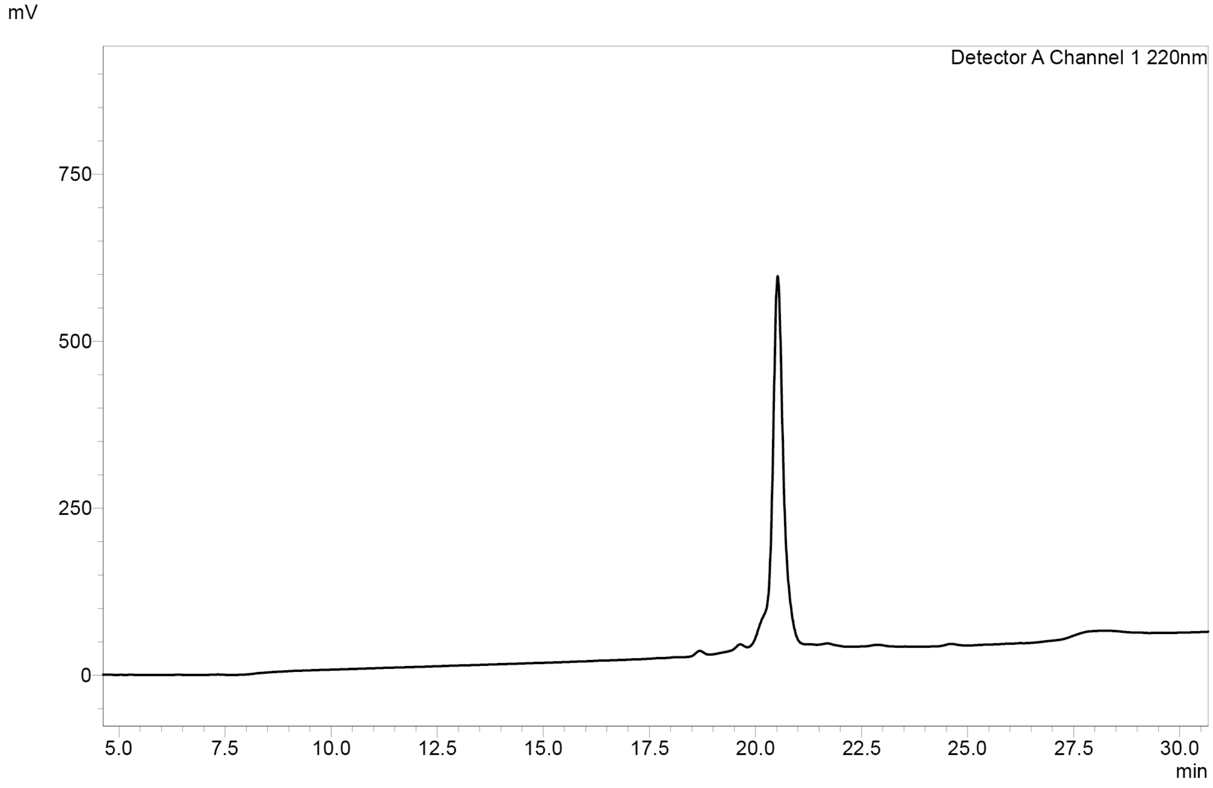

mV
Detector A Channel 1 220nm
750
500
250
0
5.0
7.5
10.0
12.5
15.0
17.5
20.0
22.5
25.0
27.5
30.0
min

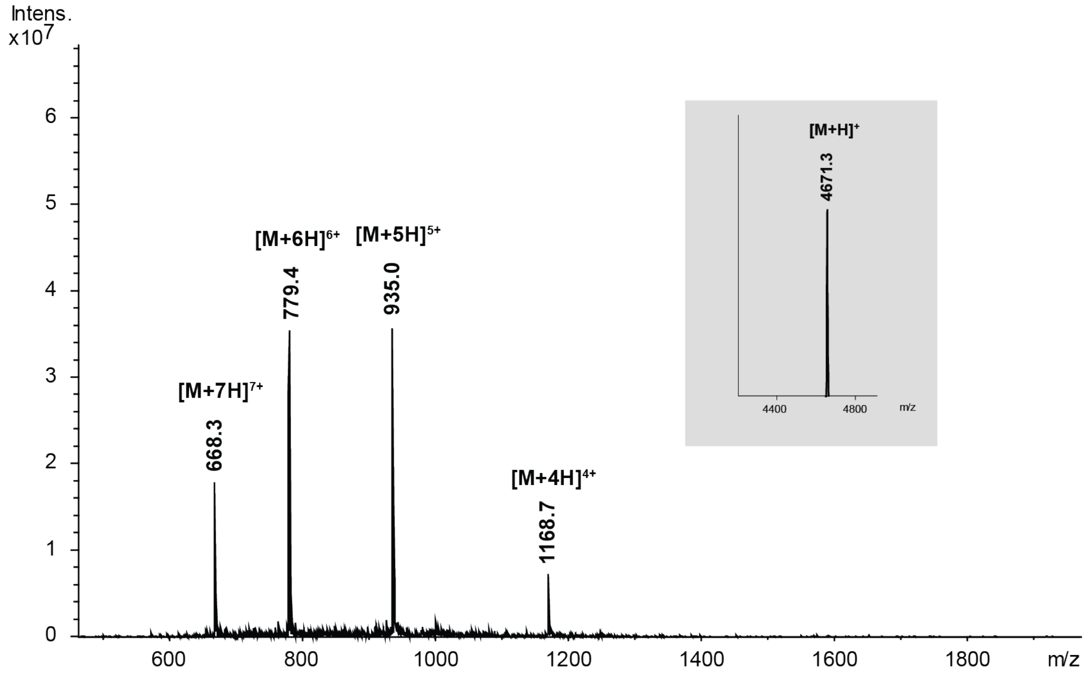

Intens.
x10^7
6
5
4
3
2
1
0
[M+7H]^7+
668.3
[M+6H]^6+
779.4
[M+5H]^5+
935.0
[M+4H]^4+
1168.7
[M+H]^+
4671.3
4400
4800
m/z
600
800
1000
1200
1400
1600
1800
m/z

(e) SIH-4

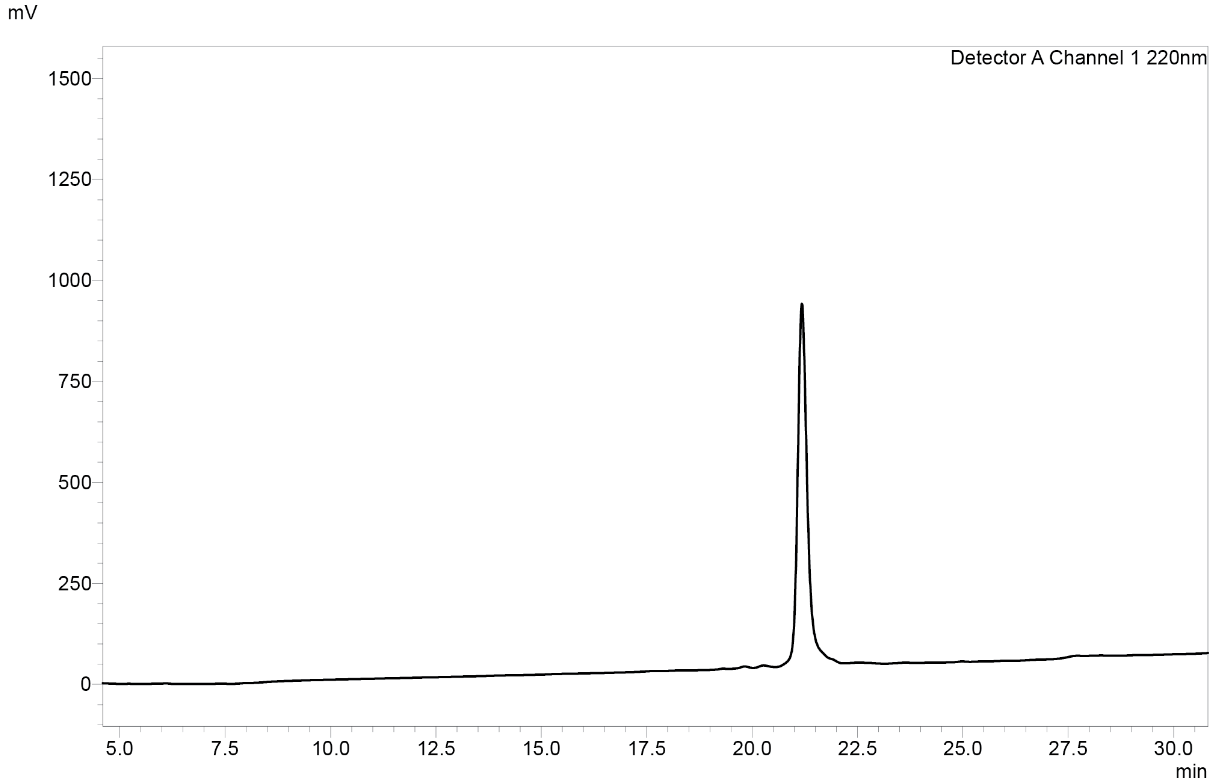
mV
Detector A Channel 1 220nm
1500
1250
1000
750
500
250
0
5.0
7.5
10.0
12.5
15.0
17.5
20.0
22.5
25.0
27.5
30.0
min

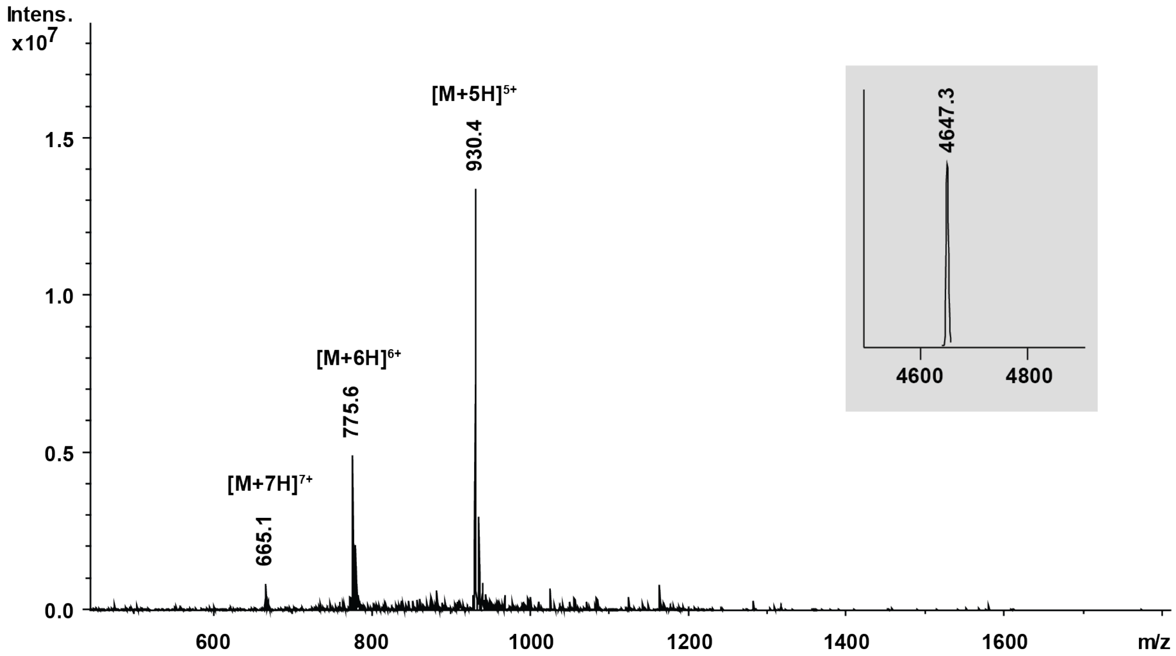
Intens.
x10^7
1.5
1.0
0.5
0.0
[M+5H]$^{5+}$
930.4
[M+6H]$^{6+}$
775.6
[M+7H]$^{7+}$
665.1
600
800
1000
1200
1400
1600
m/z
4647.3
4600
4800

(f) SIH-5

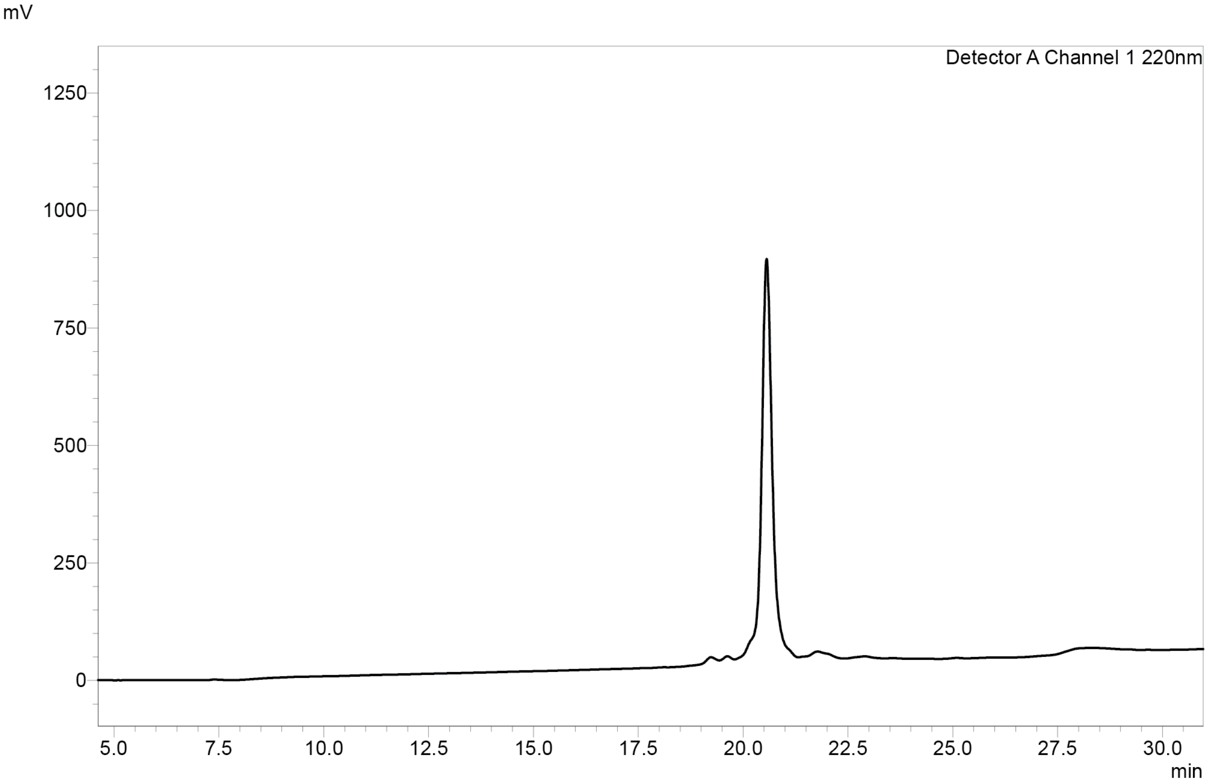

mV
Detector A Channel 1 220nm
1250
1000
750
500
250
0
5.0
7.5
10.0
12.5
15.0
17.5
20.0
22.5
25.0
27.5
30.0
min

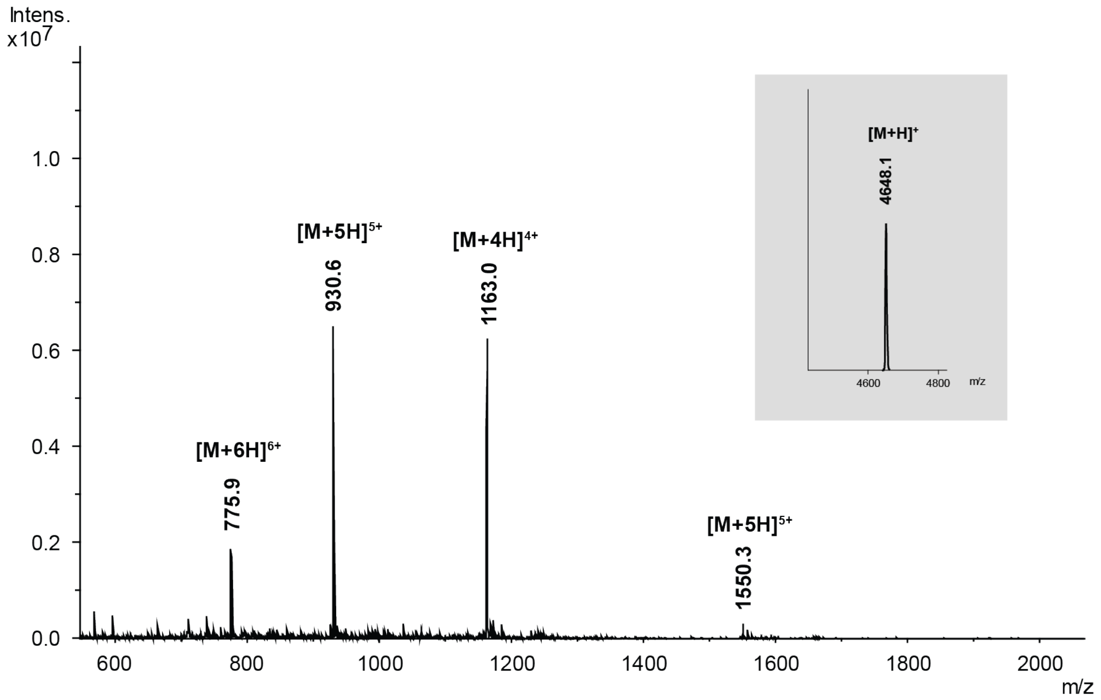

Intens.
x10^7
1.0
0.8
0.6
0.4
0.2
0.0
[M+6H]6+
775.9
[M+5H]5+
930.6
[M+4H]4+
1163.0
[M+5H]5+
1550.3
[M+H]+
4648.1
4600
4800
m/z
600
800
1000
1200
1400
1600
1800
2000
m/z

(g) SIH-6

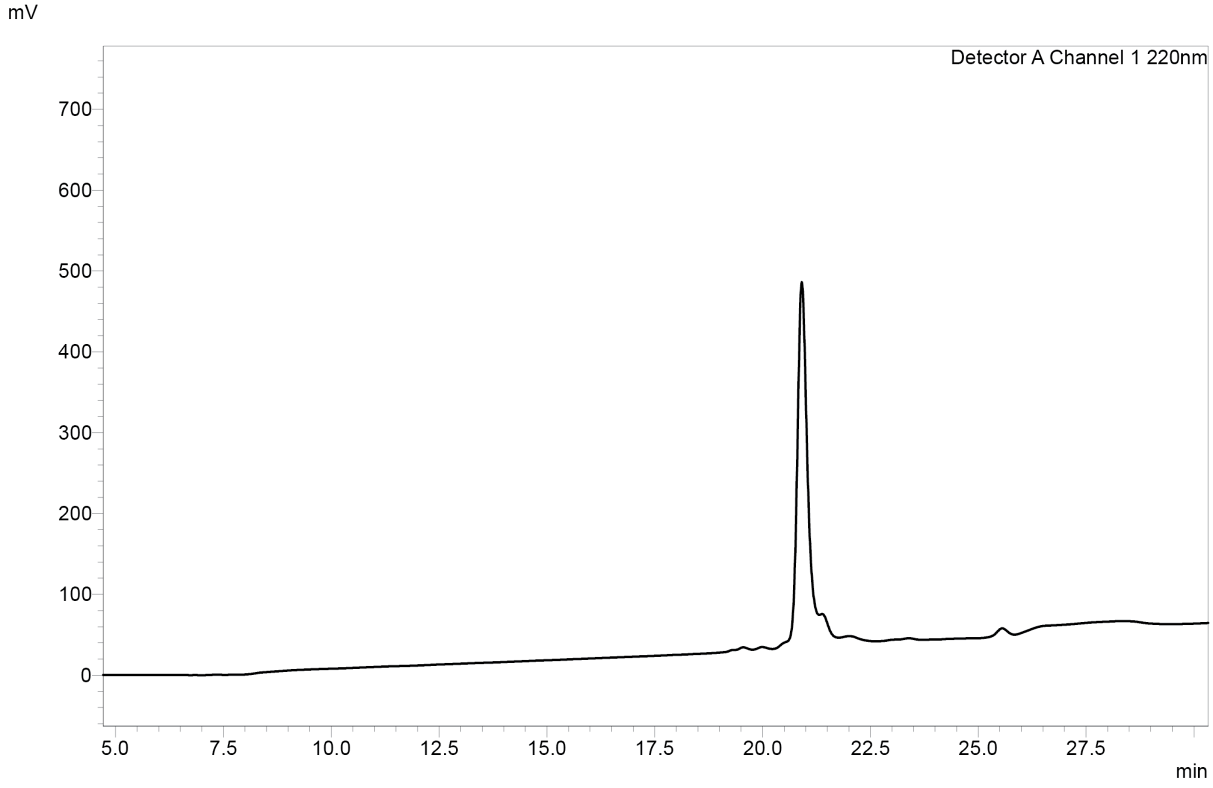

mV
Detector A Channel 1 220nm
700
600
500
400
300
200
100
0
5.0
7.5
10.0
12.5
15.0
17.5
20.0
22.5
25.0
27.5
min

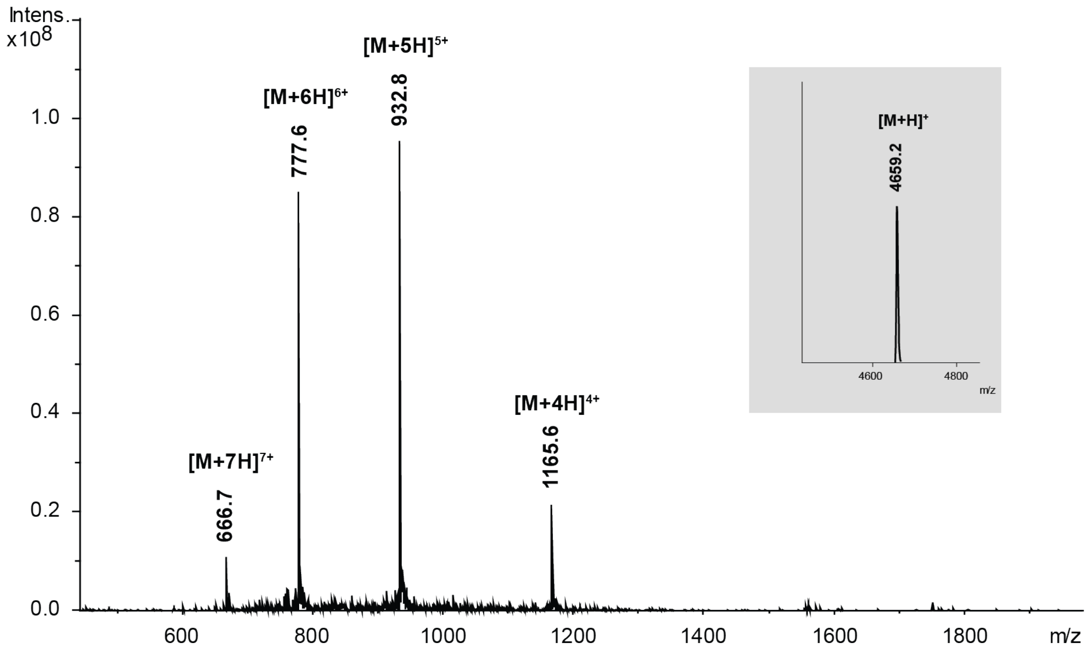

Intens.
x10^8
1.0
0.8
0.6
0.4
0.2
0.0
[M+7H]^7+
666.7
[M+6H]^6+
777.6
[M+5H]^5+
932.8
[M+4H]^4+
1165.6
[M+H]^+
4659.2
4600
4800
m/z
600
800
1000
1200
1400
1600
1800
m/z

(h) SIH-7

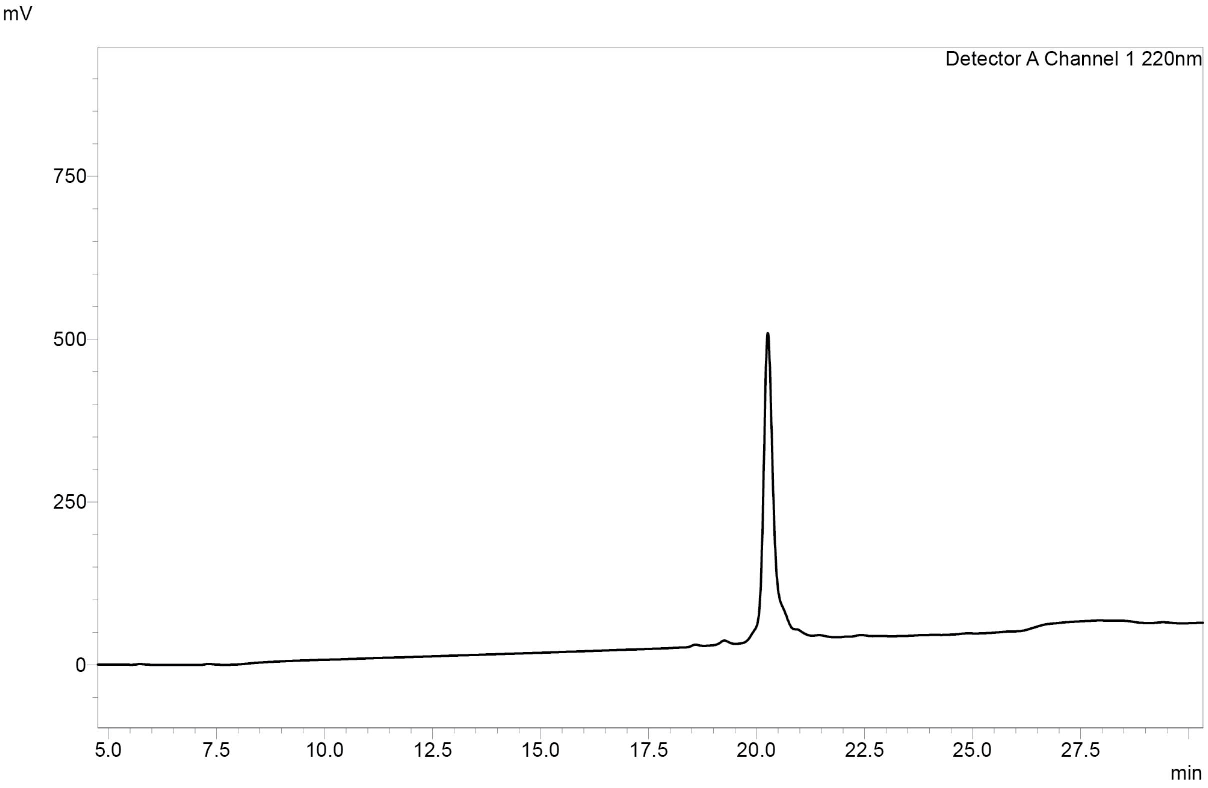

mV
Detector A Channel 1 220nm
750
500
250
0
5.0
7.5
10.0
12.5
15.0
17.5
20.0
22.5
25.0
27.5
min

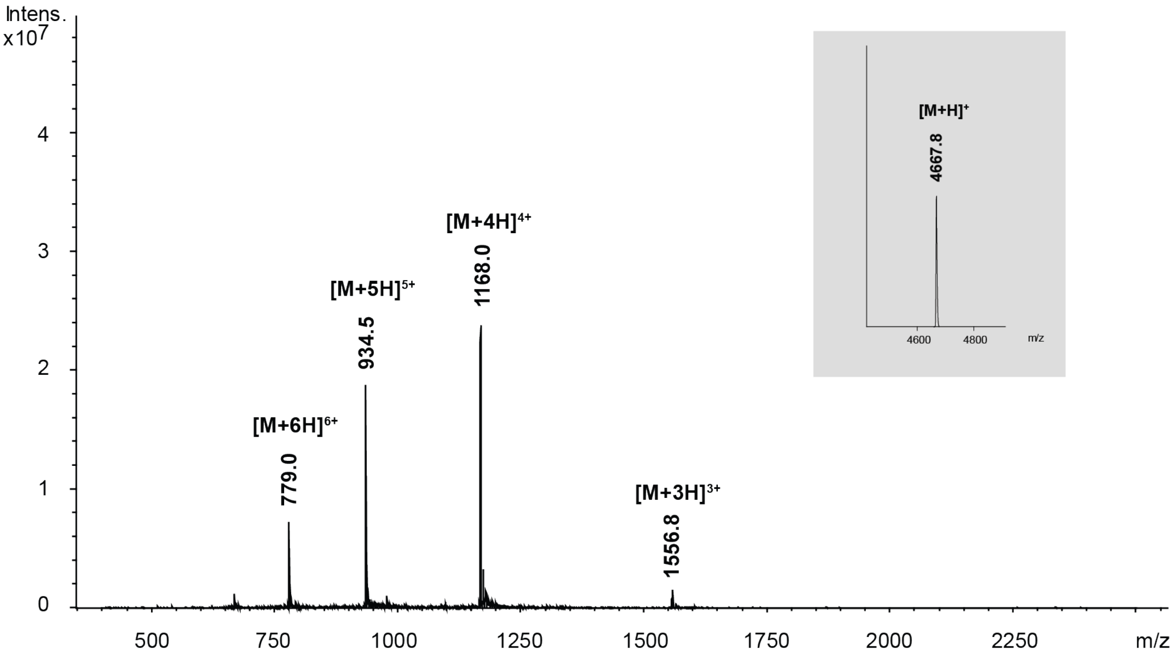

Intens.
x10^7
4
3
2
1
0
[M+6H]6+
779.0
[M+5H]5+
934.5
[M+4H]4+
1168.0
[M+3H]3+
1556.8
500
750
1000
1250
1500
1750
2000
2250
m/z
[M+H]+
4667.8
4600
4800
m/z

(i) SIH-8

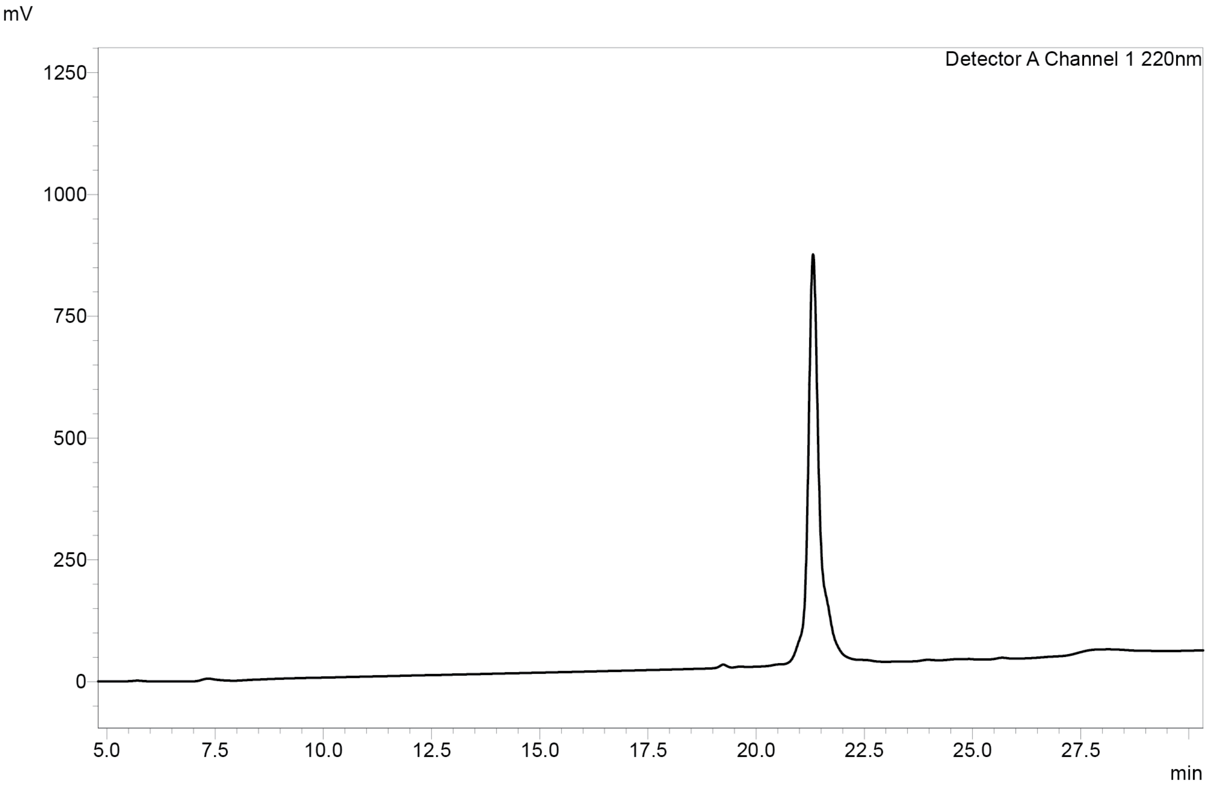

mV
Detector A Channel 1 220nm
1250
1000
750
500
250
0
5.0
7.5
10.0
12.5
15.0
17.5
20.0
22.5
25.0
27.5
min

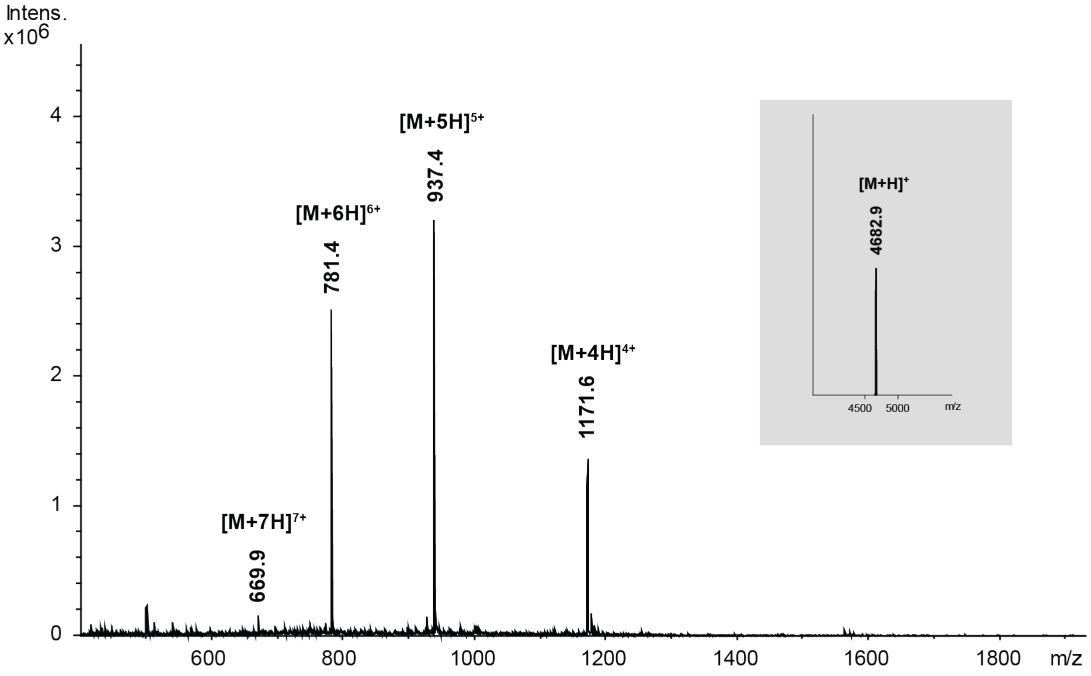

Intens.
x10^6
[M+7H]7+
669.9
[M+6H]6+
781.4
[M+5H]5+
937.4
[M+4H]4+
1171.6
[M+H]+
4682.9
4500
5000
m/z
600
800
1000
1200
1400
1600
1800
m/z

(j) SIH-9

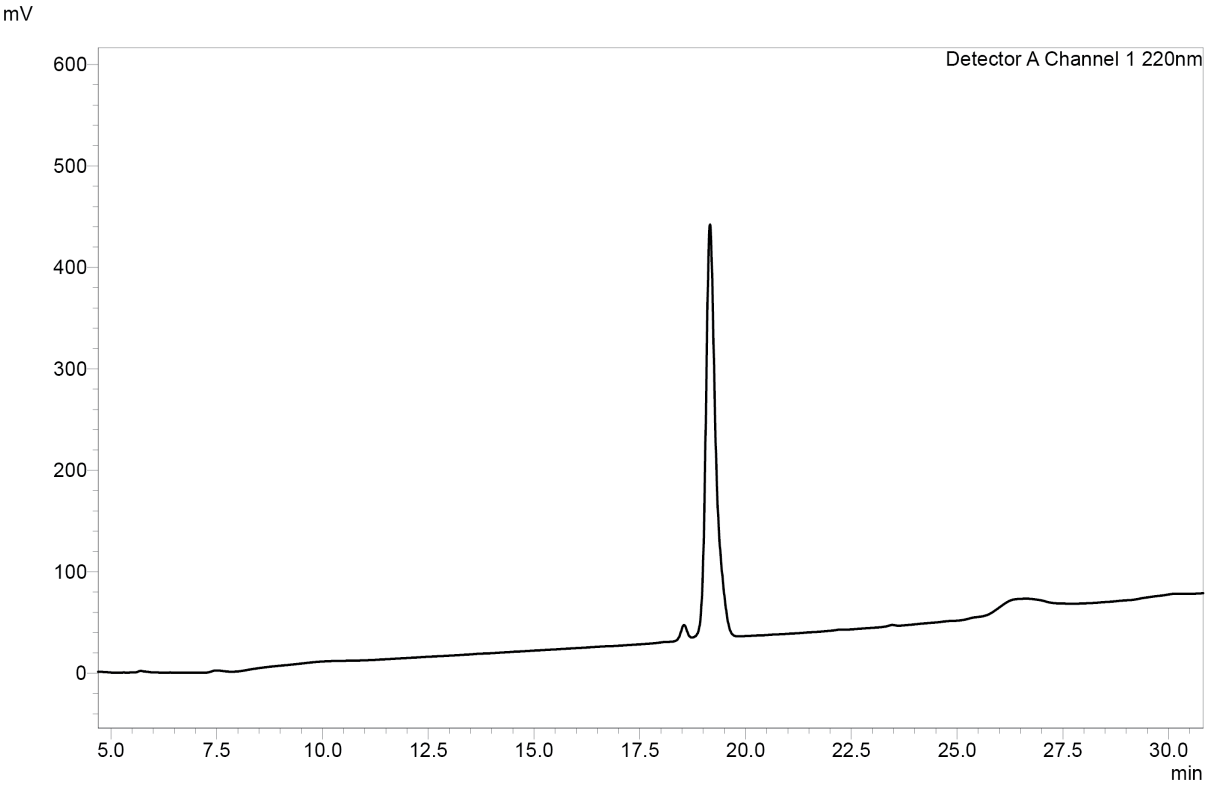
mV
Detector A Channel 1 220nm
600
500
400
300
200
100
0
5.0
7.5
10.0
12.5
15.0
17.5
20.0
22.5
25.0
27.5
30.0
min

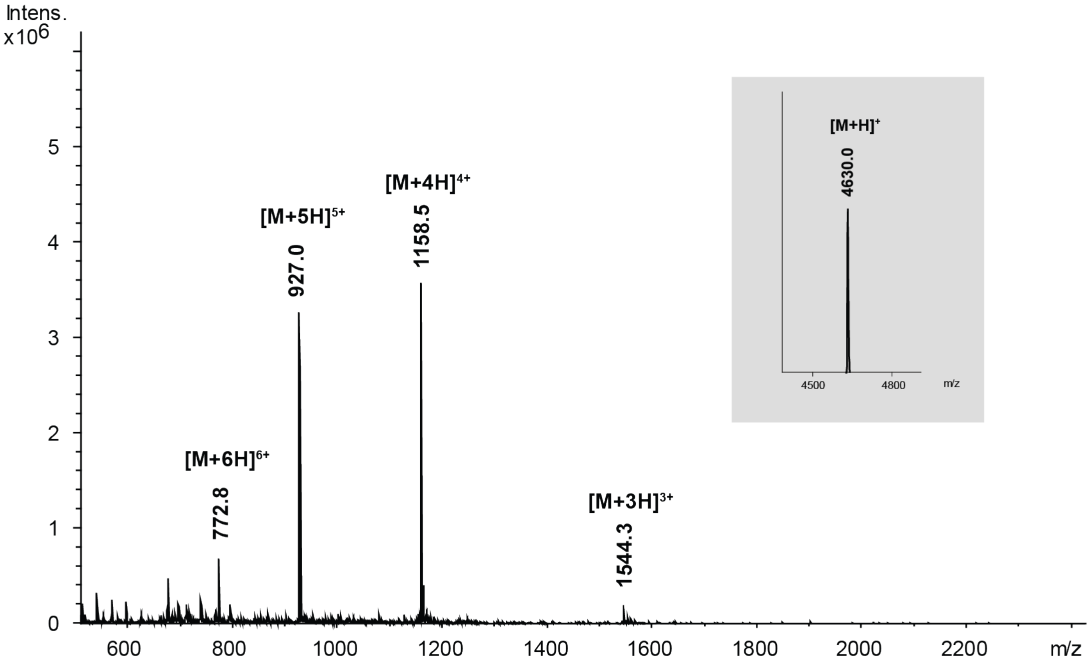
Intens.
x10^6
[M+6H]^6+
772.8
[M+5H]^5+
927.0
[M+4H]^4+
1158.5
[M+3H]^3+
1544.3
[M+H]^+
4630.0
4500
4800
m/z
600
800
1000
1200
1400
1600
1800
2000
2200
m/z

(k) SIH-10

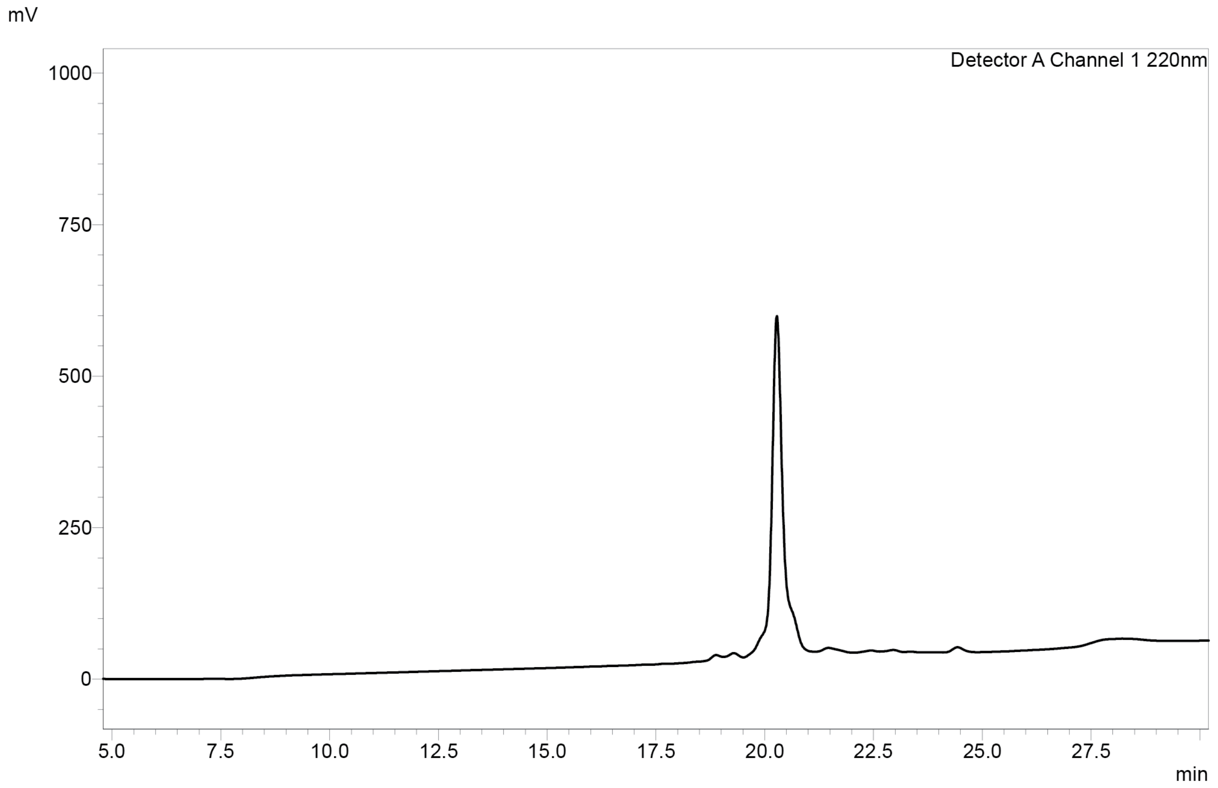
mV
Detector A Channel 1 220nm
1000
750
500
250
0
5.0
7.5
10.0
12.5
15.0
17.5
20.0
22.5
25.0
27.5
min

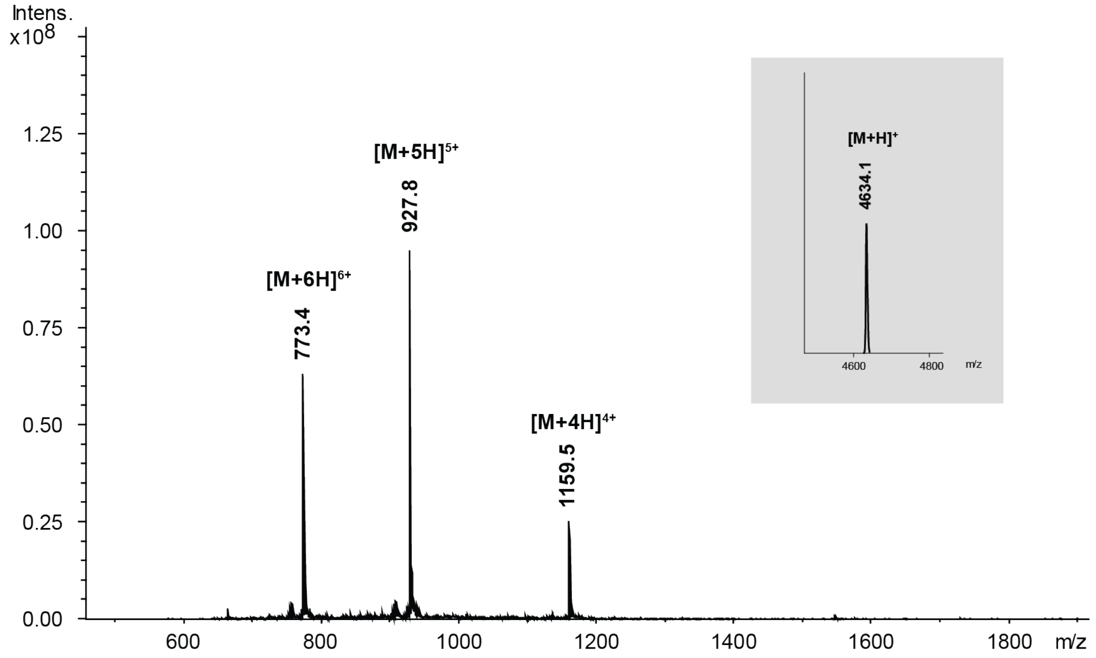
Intens.
x10^8
1.25
1.00
0.75
0.50
0.25
0.00
[M+6H]6+
773.4
[M+5H]5+
927.8
[M+4H]4+
1159.5
600
800
1000
1200
1400
1600
1800
m/z
[M+H]+
4634.1
4600
4800
m/z

(l) SIH-11

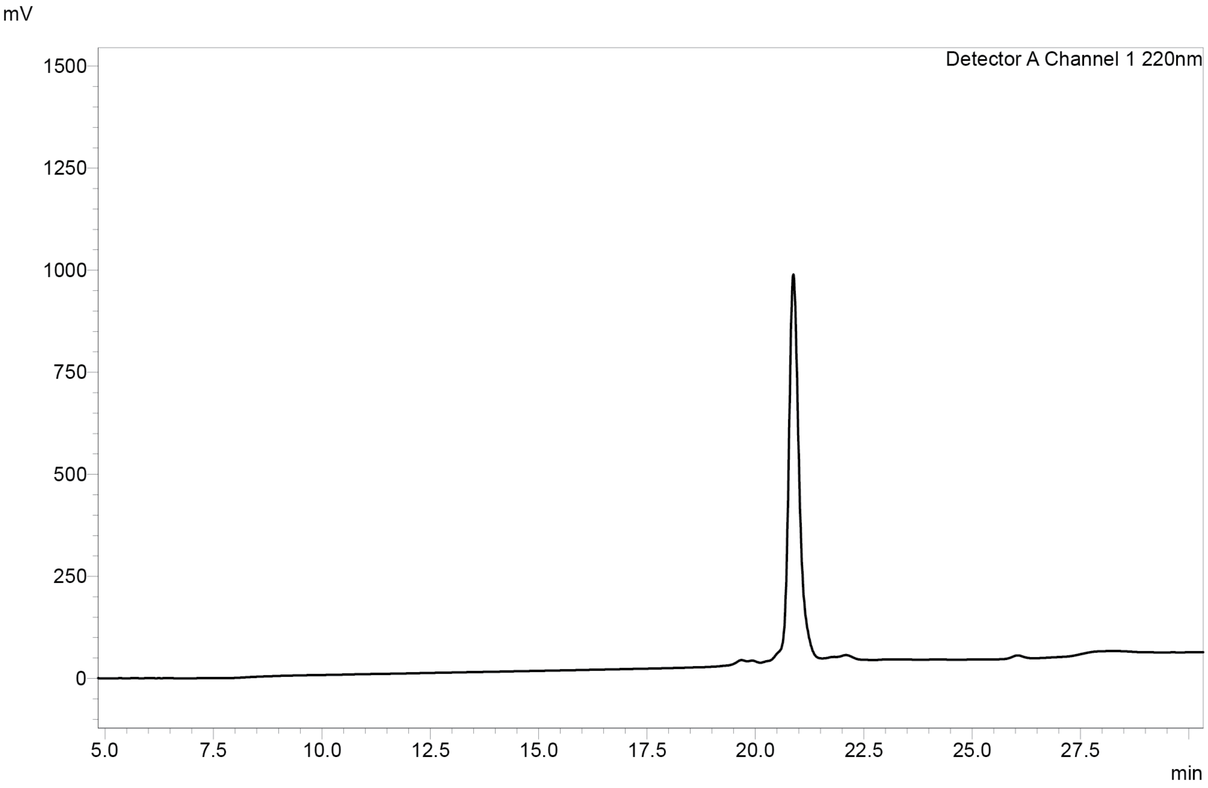
mV
1500
1250
1000
750
500
250
0
Detector A Channel 1 220nm
5.0
7.5
10.0
12.5
15.0
17.5
20.0
22.5
25.0
27.5
min

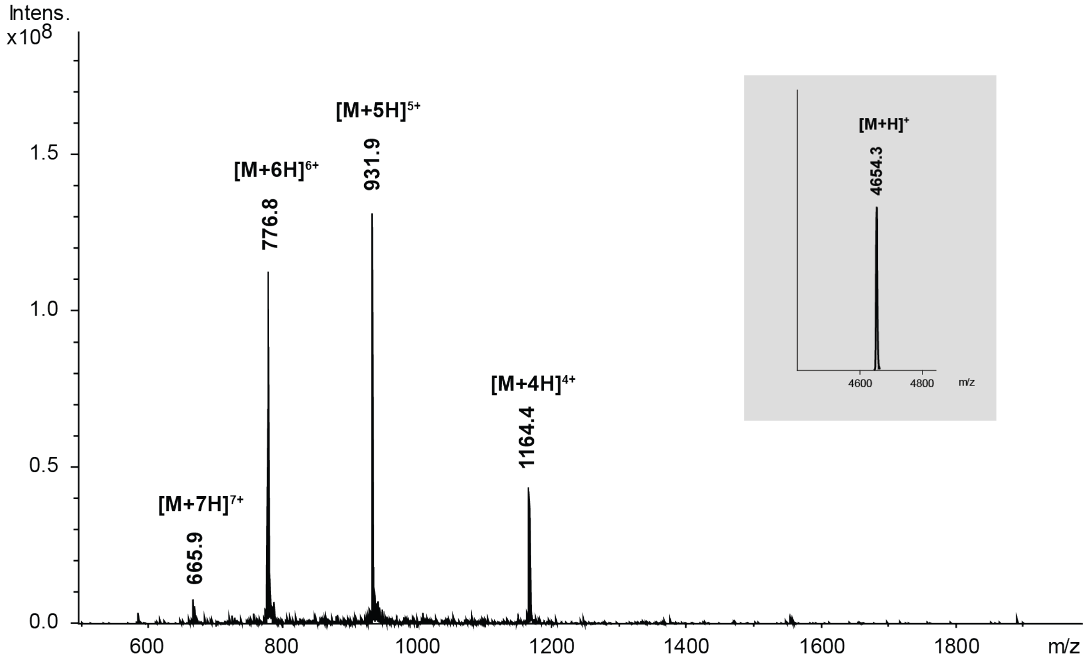
Intens.
x10^8
1.5
1.0
0.5
0.0
[M+7H]^7+
665.9
[M+6H]^6+
776.8
[M+5H]^5+
931.9
[M+4H]^4+
1164.4
600
800
1000
1200
1400
1600
1800
m/z
[M+H]^+
4654.3
4600
4800
m/z

(m) SIH-12

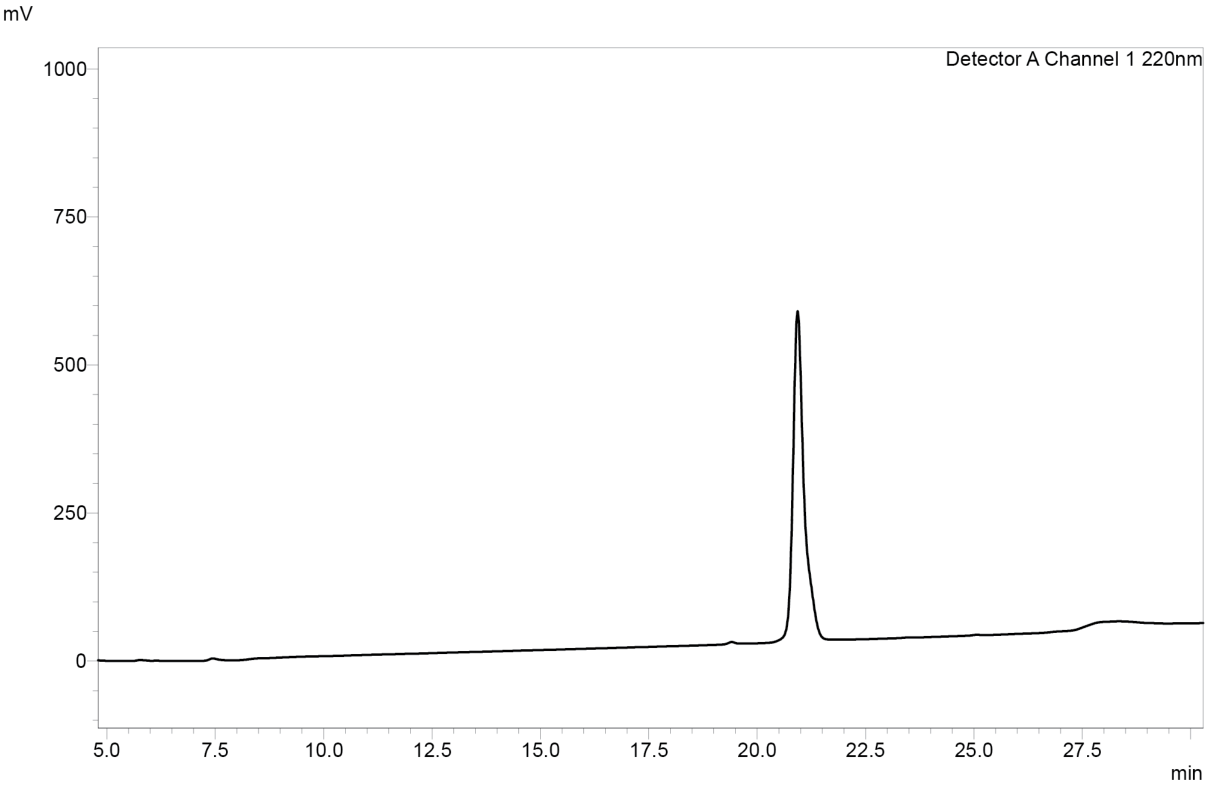
mV
Detector A Channel 1 220nm
1000
750
500
250
0
5.0
7.5
10.0
12.5
15.0
17.5
20.0
22.5
25.0
27.5
min

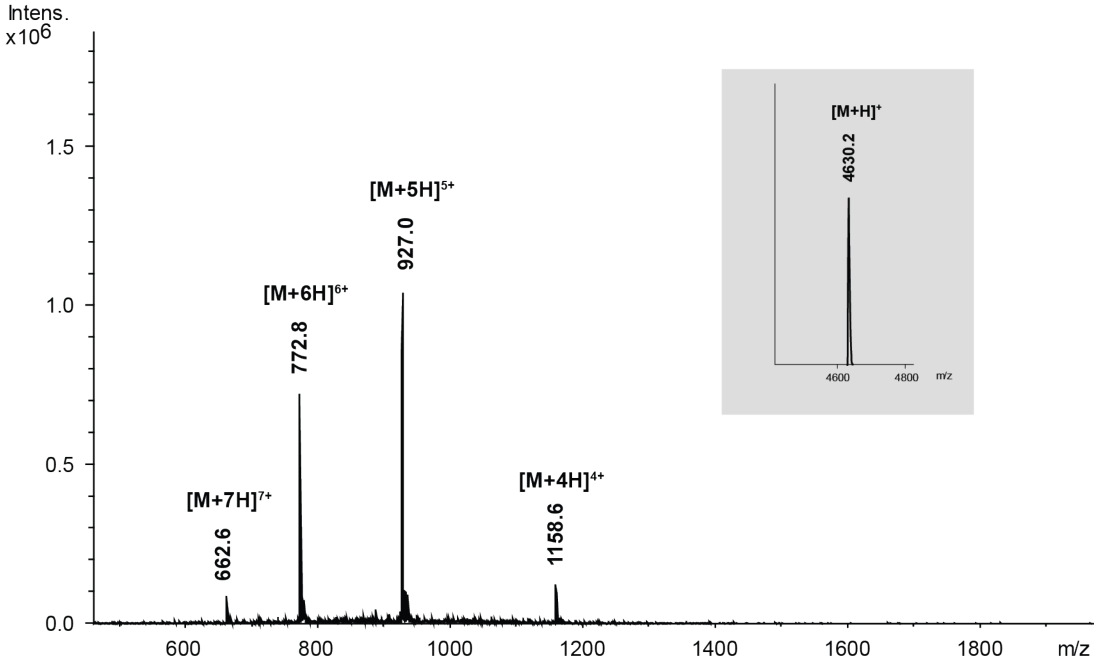
Intens.
x10^6
1.5
1.0
0.5
0.0
[M+5H]5+
927.0
[M+6H]6+
772.8
[M+7H]7+
662.6
[M+4H]4+
1158.6
[M+H]+
4630.2
4600
4800
m/z
600
800
1000
1200
1400
1600
1800
m/z

(n) SIH-13

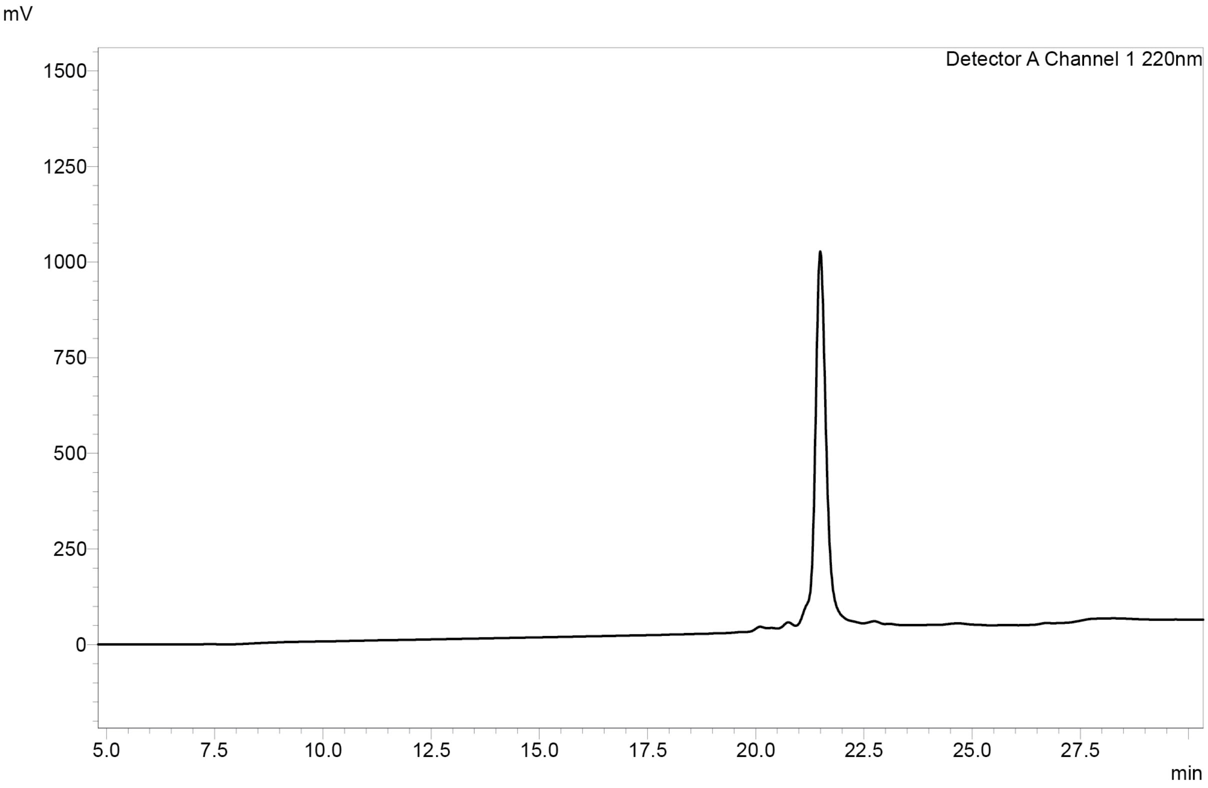
mV
Detector A Channel 1 220nm
1500
1250
1000
750
500
250
0
5.0
7.5
10.0
12.5
15.0
17.5
20.0
22.5
25.0
27.5
min

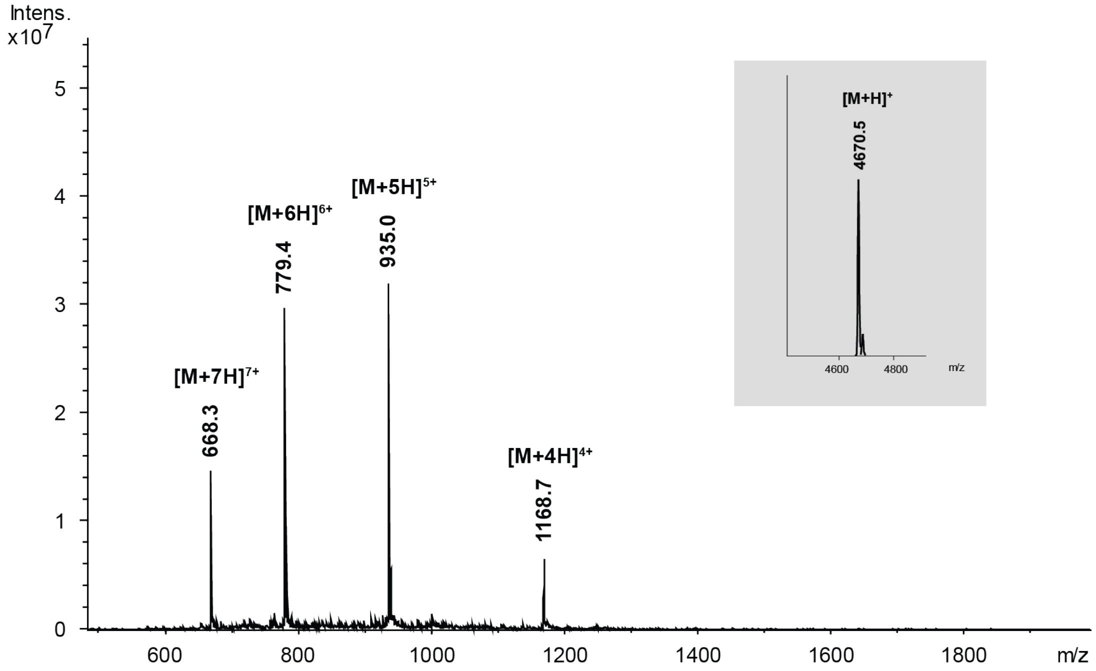
Intens.
x10^7
5
4
3
2
1
0
[M+7H]^7+
668.3
[M+6H]^6+
779.4
[M+5H]^5+
935.0
[M+4H]^4+
1168.7
[M+H]^+
4670.5
4600
4800
m/z
600
800
1000
1200
1400
1600
1800
m/z

(o) SIH-14

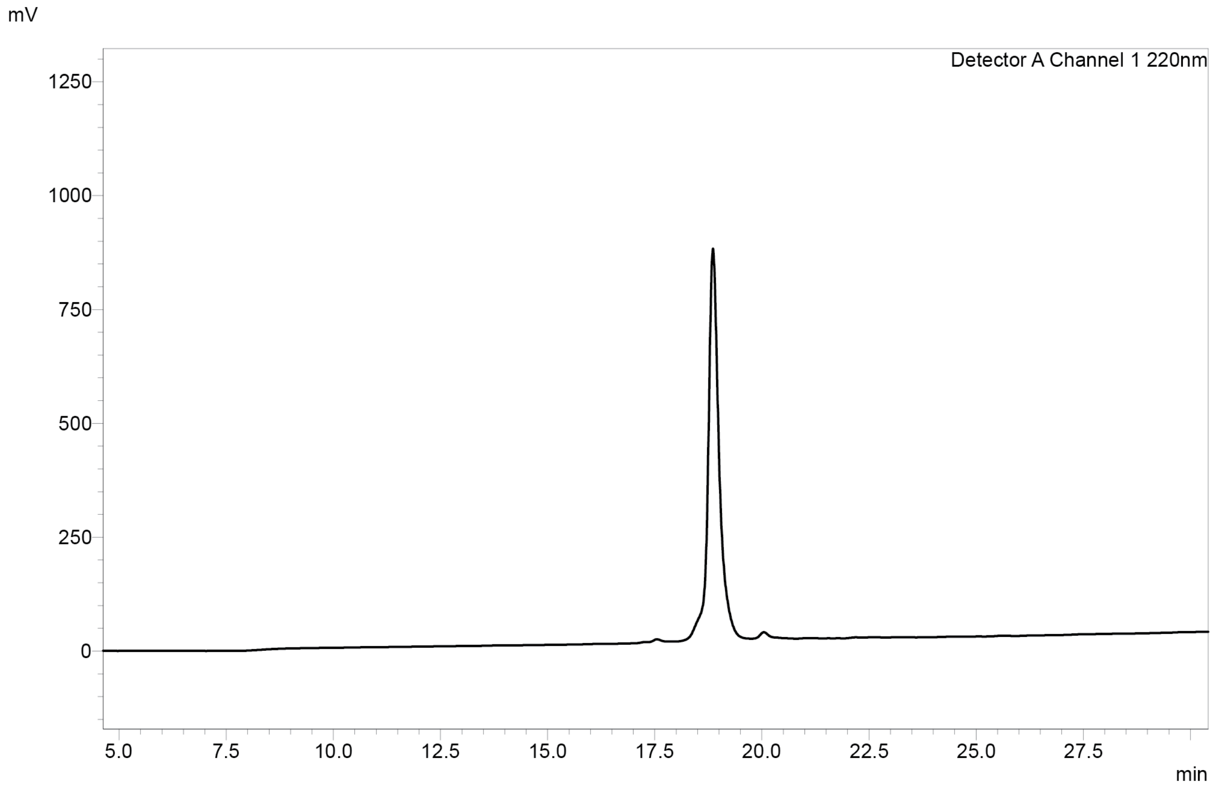

mV
Detector A Channel 1 220nm
1250
1000
750
500
250
0
5.0
7.5
10.0
12.5
15.0
17.5
20.0
22.5
25.0
27.5
min

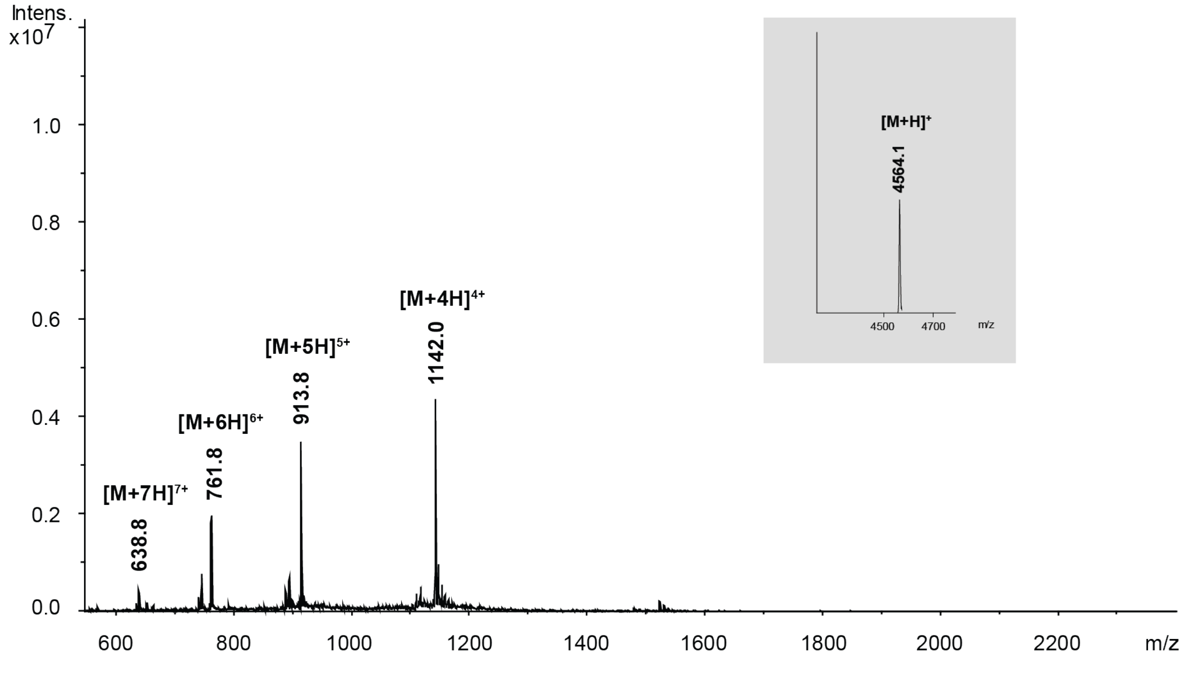

Intens.
x10^7
1.0
0.8
0.6
0.4
0.2
0.0
[M+7H]7+
638.8
[M+6H]6+
761.8
[M+5H]5+
913.8
[M+4H]4+
1142.0
600
800
1000
1200
1400
1600
1800
2000
2200
m/z
[M+H]+
4564.1
4500
4700
m/z

(p) SIH-15

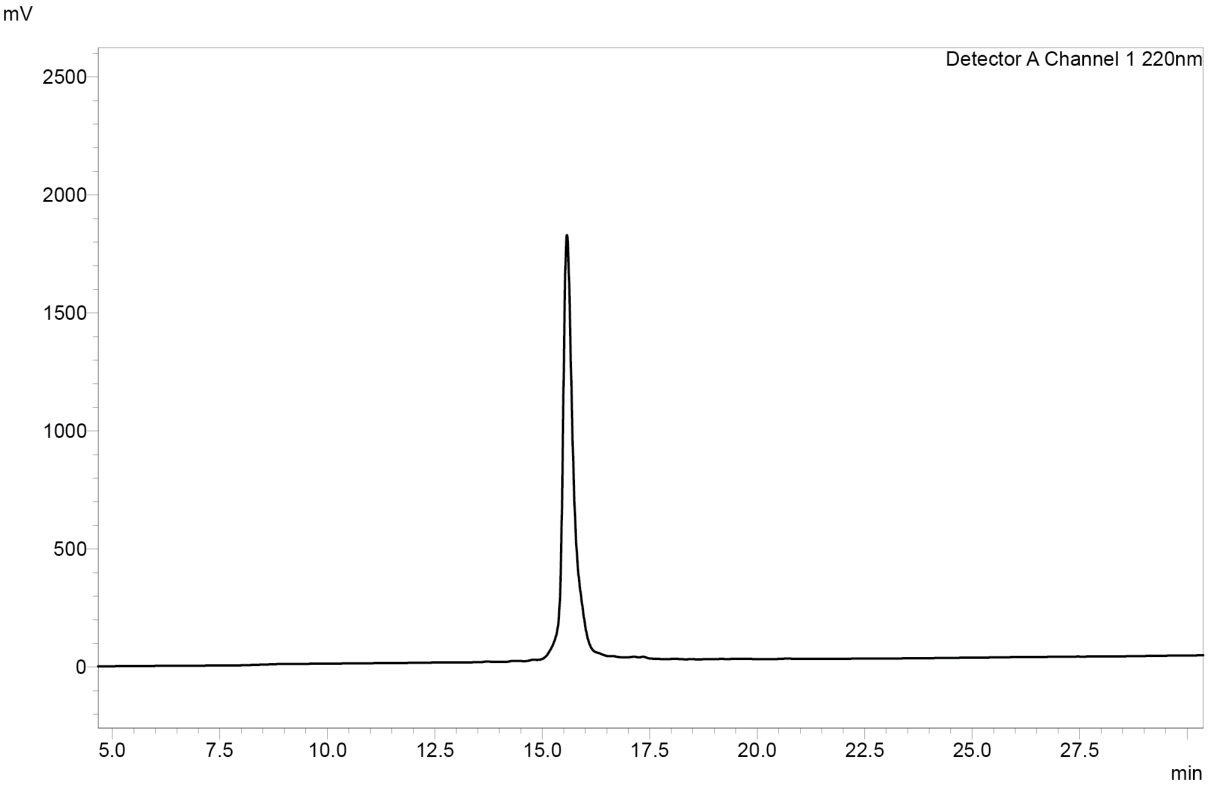

mV
Detector A Channel 1 220nm
2500
2000
1500
1000
500
0
5.0
7.5
10.0
12.5
15.0
17.5
20.0
22.5
25.0
27.5
min

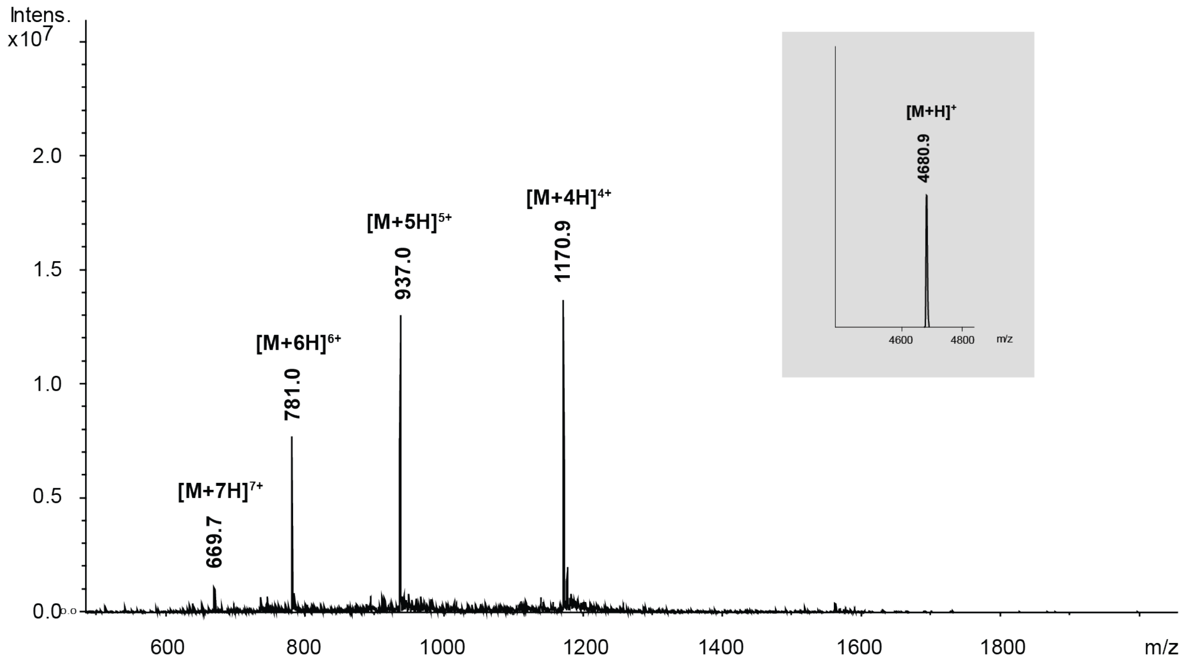

Intens.
x10^7
2.0
1.5
1.0
0.5
0.0
[M+7H]^7+
669.7
[M+6H]^6+
781.0
[M+5H]^5+
937.0
[M+4H]^4+
1170.9
[M+H]^+
4680.9
4600
4800
m/z
600
800
1000
1200
1400
1600
1800
m/z

(q) BGF-1

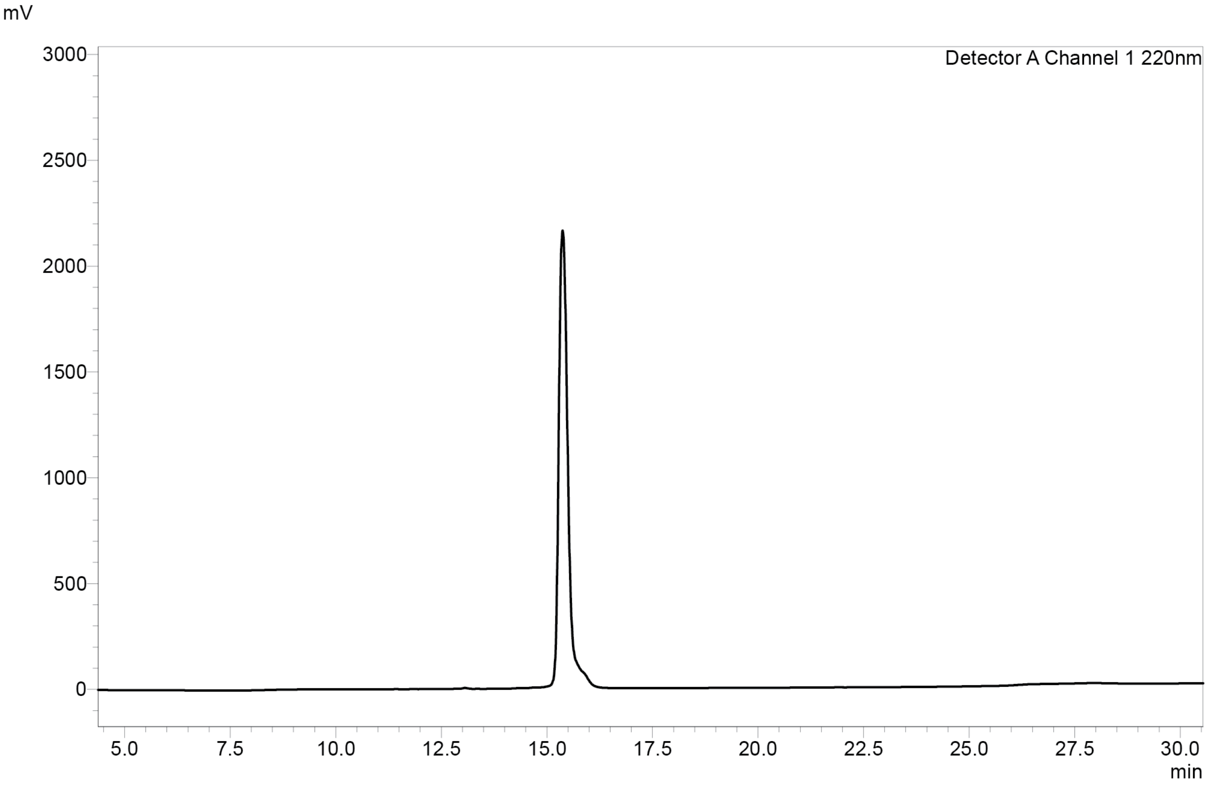

mV
3000
2500
2000
1500
1000
500
0
Detector A Channel 1 220nm
5.0
7.5
10.0
12.5
15.0
17.5
20.0
22.5
25.0
27.5
30.0
min

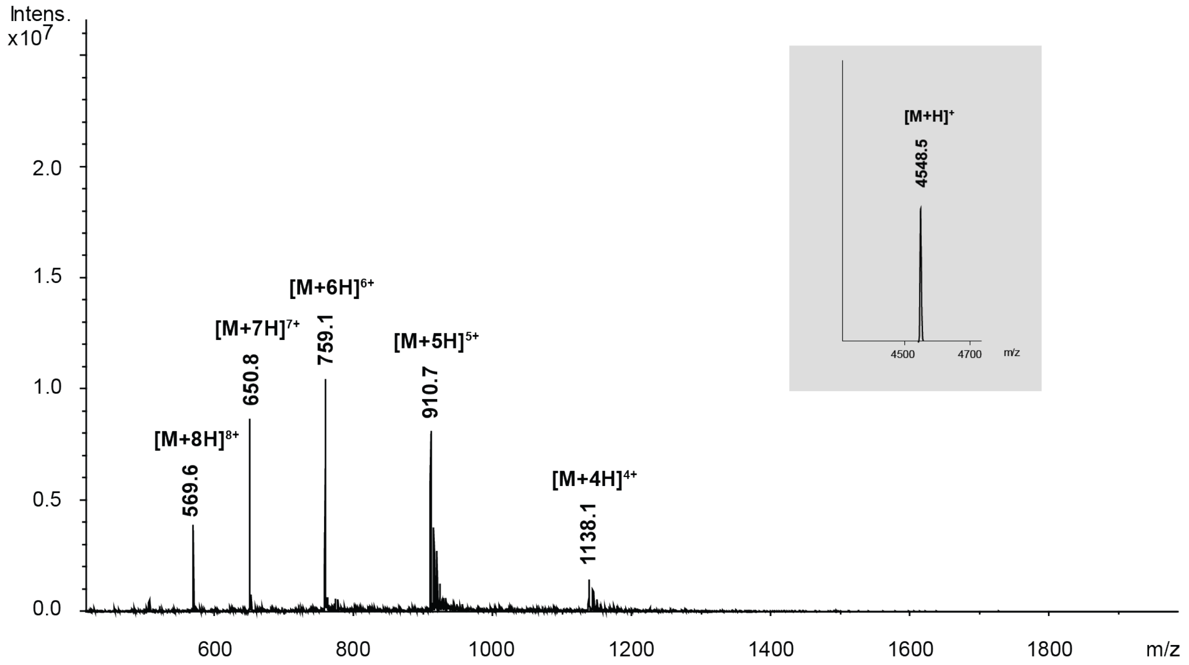

Intens.
x10^7
2.0
1.5
1.0
0.5
0.0
[M+8H]8+
569.6
[M+7H]7+
650.8
[M+6H]6+
759.1
[M+5H]5+
910.7
[M+4H]4+
1138.1
[M+H]+
4548.5
4500
4700
m/z
600
800
1000
1200
1400
1600
1800
m/z

(r) BGF-2

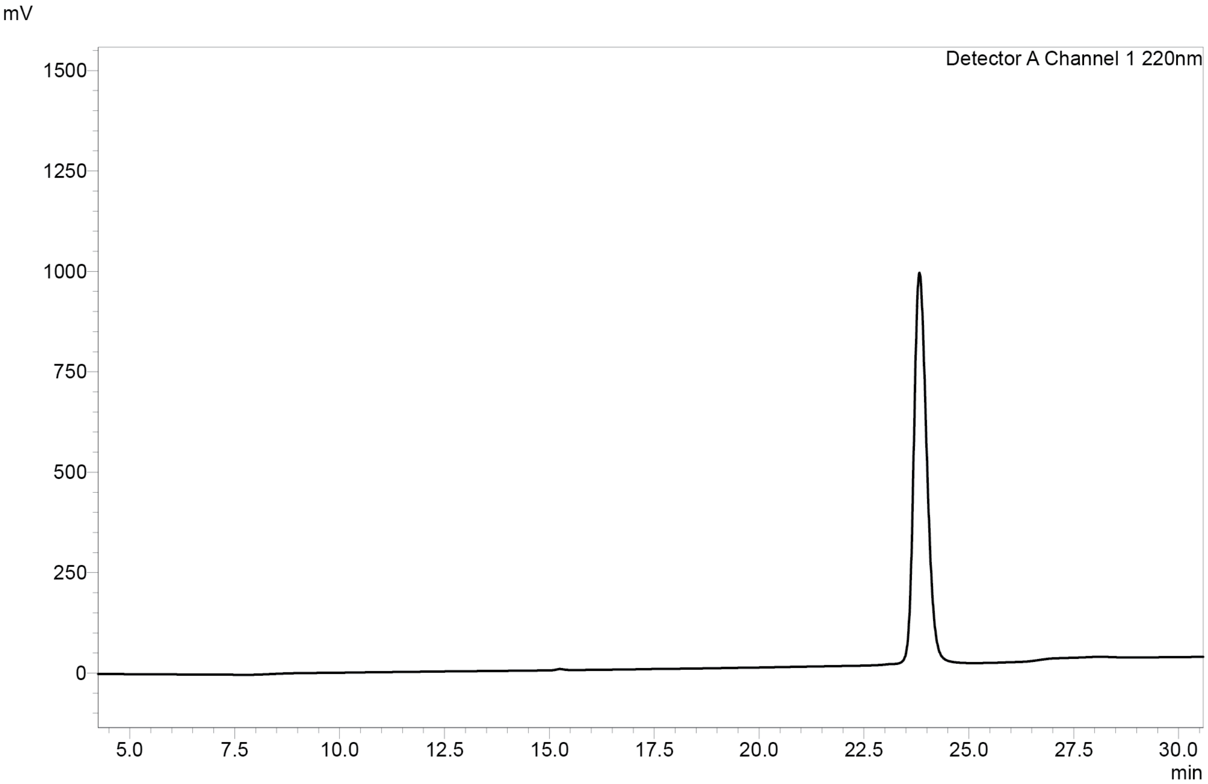

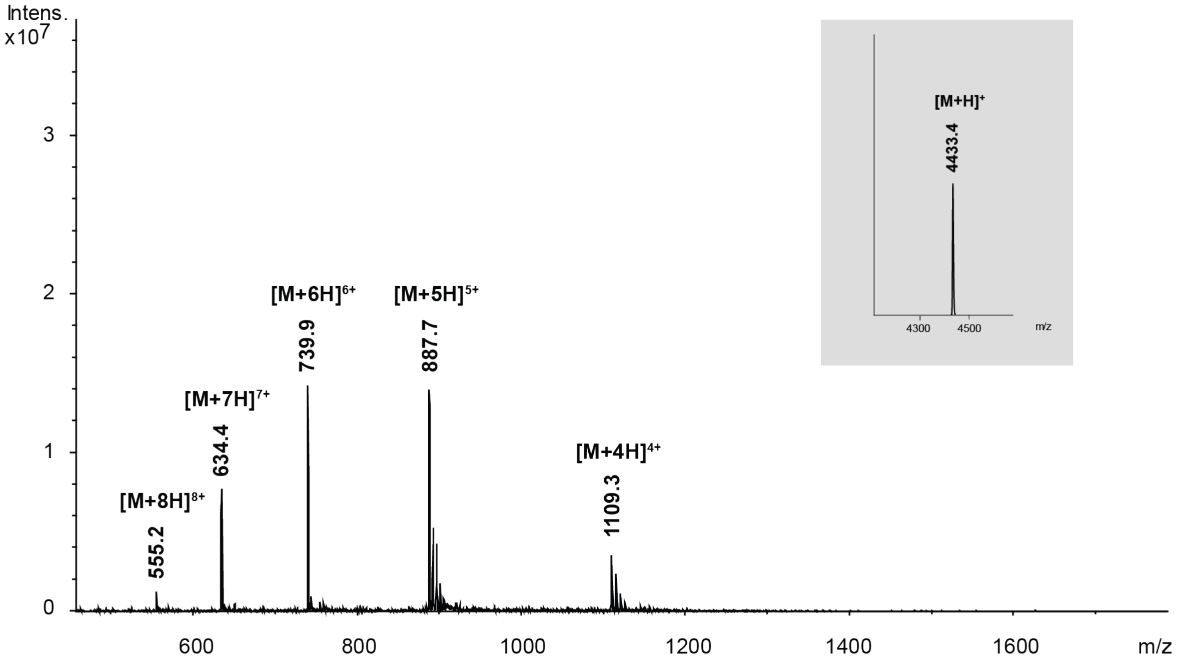

Analytical HPLC chromatograms (top panels) and ESI-MS spectrum (bottom panels) of purified peptides used in this study.

Supplementary References

1. Tang, G. et al. EMAN2: An extensible image processing suite for electron microscopy. *J. Struct. Biol.* **157**, 38-46 (2007).
2. Reboul, C.F., Eager, M., Elmlund, D. & Elmlund, H. Single-particle cryo-EM—Improved ab initio 3D reconstruction with SIMPLE/PRIME. *Protein Sci.* **27**, 51-61 (2018).
3. Li, X. et al. Electron counting and beam-induced motion correction enable near-atomic-resolution single-particle cryo-EM. *Nat. Methods* **10**, 584-590 (2013).
4. Scheres, S.H.W. RELION: Implementation of a Bayesian approach to cryo-EM structure determination. *J. Struct. Biol.* **180**, 519-530 (2012).
5. Pettersen, E.F. et al. UCSF Chimera--a visualization system for exploratory research and analysis. *J. Comput. Chem.* **25**, 1605-1612 (2004).
6. Pettersen, E.F. et al. UCSF ChimeraX: Structure visualization for researchers, educators, and developers. *Protein Sci.* **30**, 70-82 (2021).
7. Goddard, T.D. et al. UCSF ChimeraX: Meeting modern challenges in visualization and analysis. *Protein Sci.* **27**, 14-25 (2018).
8. Kucukelbir, A., Sigworth, F.J. & Tagare, H.D. Quantifying the local resolution of cryo-EM density maps. *Nat. Methods* **11**, 63-65 (2014).
