# Supplementary material for: A dimeric proteomimetic prevents SARS-CoV-2 infection by dimerizing the spike protein
Source: Nat Chem Biol. 2022 Jun 2;18(10):1046–55. doi: 10.1038/s41589-022-01060-0 (PMC9512702; doi:10.1038/s41589-022-01060-0)
Supplement: Supplementary file 2 — Reporting Summary [file 41589_2022_1060_MOESM2_ESM.pdf]

nature research

Corresponding author(s): Jayanta Chatterjee

Last updated by author(s): Apr 22, 2022

Reporting Summary

Nature Research wishes to improve the reproducibility of the work that we publish. This form provides structure for consistency and transparency in reporting. For further information on Nature Research policies, see our Editorial Policies and the Editorial Policy Checklist.

Statistics

For all statistical analyses, confirm that the following items are present in the figure legend, table legend, main text, or Methods section.

n/a	Confirmed	
☐	☒	The exact sample size (*n*) for each experimental group/condition, given as a discrete number and unit of measurement
☐	☒	A statement on whether measurements were taken from distinct samples or whether the same sample was measured repeatedly
☐	☒	The statistical test(s) used AND whether they are one- or two-sided<br>*Only common tests should be described solely by name; describe more complex techniques in the Methods section.*
☒	☐	A description of all covariates tested
☒	☐	A description of any assumptions or corrections, such as tests of normality and adjustment for multiple comparisons
☐	☒	A full description of the statistical parameters including central tendency (e.g. means) or other basic estimates (e.g. regression coefficient) AND variation (e.g. standard deviation) or associated estimates of uncertainty (e.g. confidence intervals)
☐	☒	For null hypothesis testing, the test statistic (e.g. *F*, *t*, *r*) with confidence intervals, effect sizes, degrees of freedom and *P* value noted<br>*Give P values as exact values whenever suitable.*
☒	☐	For Bayesian analysis, information on the choice of priors and Markov chain Monte Carlo settings
☒	☐	For hierarchical and complex designs, identification of the appropriate level for tests and full reporting of outcomes
☒	☐	Estimates of effect sizes (e.g. Cohen's *d*, Pearson's *r*), indicating how they were calculated

Our web collection on statistics for biologists contains articles on many of the points above.

Software and code

Policy information about availability of computer code

Data collection	NanoDrop 2000c 1.6 (ThermoFisher); LabSolutions 5.93 (Shimadzu); esquireControl 6.2 (Bruker Daltonik); JASCO Spectra Manager 1.1.53.00; Optima Proteome Lab XL-I (Beckman coulter); ProteOn Manager 3.1 (Bio-Rad); PR.ThermControl (NanoTemper Technologies); ASTRA 6.1 (Wyatt Technology); Gen5 (BioTek), Latitude-S 3.5 (Gatan), Malvern Zetasizer 7.11 (Malvern).
Data analysis	ESI Compass 1.3 DataAnalysis 4.0 (Bruker Daltonik) for MS, SEDNTERP 3.0.3, Sedfit 16-1c, WinREEDIT 0.999.0028, WINNONLIN 1.060 for AUC; ProteOn Manager 3.1 for SPR; ASTRA 6.1 (Wyatt Technology) for SEC-MALS; EMAN2.1, SIMPLE 2.0 package, RELION 3.1, MotionCor2 1.2.1, cisTEM 1.0.0-beta, CTFFIND 4.1.13, ResMap 1.1.4 for EM data processing; UCSF Chimera 1.13.1, UCSF Chimera X 1.2.5, PHENIX 1.18.2, COOT 0.9.8 for EM data analysis, PyMOL 2.4.0, GraphPad Prism 8.0.1.; Malvern Zetasizer 7.11 for DLS.

For manuscripts utilizing custom algorithms or software that are central to the research but not yet described in published literature, software must be made available to editors and reviewers. We strongly encourage code deposition in a community repository (e.g. GitHub). See the Nature Research guidelines for submitting code & software for further information.

Data

Policy information about availability of data

All manuscripts must include a data availability statement. This statement should provide the following information, where applicable:

- Accession codes, unique identifiers, or web links for publicly available datasets
- A list of figures that have associated raw data
- A description of any restrictions on data availability

ProtParam tool (ExPASY Server). PDB IDs: 7JZU, 1ROP, 1F4M, 1BGF, 7KMS. The cryo-EM maps and atomic models have been deposited in the Electron Microscopy Data Bank (EMDB) and the Protein Data Bank (PDB) with accession codes: EMD-32388, EMD-33042, and PDB entry ID 7X7N. All other data is present as Source Data Files or in the Supplementary Information.

April 2020

Field-specific reporting

Please select the one below that is the best fit for your research. If you are not sure, read the appropriate sections before making your selection.

☒ Life sciences ☐ Behavioural & social sciences ☐ Ecological, evolutionary & environmental sciences

For a reference copy of the document with all sections, see nature.com/documents/nr-reporting-summary-flat.pdf

Life sciences study design

All studies must disclose on these points even when the disclosure is negative.

Sample size	Statistical methods to determine sample size were not conducted. Sample size of data were chosen that yielded good fits or statistical significance of the data for clear interpretation.
Data exclusions	No data was excluded from analysis.
Replication	The biochemical and biophysical experiments were performed in duplicates or triplicates and repeated twice or thrice. All attempts at replication were successful and the deviation in the measurements are depicted in the graphs.
Randomization	Since the research did not involve experimental groups, randomization is inapplicable.
Blinding	Virus neutralization assays were blinded. In other experiments, blinding is not relevant as a sequential design approach to derive the SIH peptides was used.

Reporting for specific materials, systems and methods

We require information from authors about some types of materials, experimental systems and methods used in many studies. Here, indicate whether each material, system or method listed is relevant to your study. If you are not sure if a list item applies to your research, read the appropriate section before selecting a response.

Materials & experimental systems

n/a	Involved in the study	
☒	☐	Antibodies
☐	☒	Eukaryotic cell lines
☒	☐	Palaeontology and archaeology
☐	☒	Animals and other organisms
☒	☐	Human research participants
☒	☐	Clinical data
☒	☐	Dual use research of concern

Methods

n/a	Involved in the study	
☒	☐	ChIP-seq
☒	☐	Flow cytometry
☒	☐	MRI-based neuroimaging

Eukaryotic cell lines

Policy information about cell lines

Cell line source(s)	BEI Resources, 293T-hACE-2 cell line was submitted by Dr. Jesse Bloom in the resource. Expi293F (Gibco). HEK293T cells were obtained from Dr. John Moore, Weill Cornell Medicine, NY, USA. Vero E6 cell line was obtained from the cell repository of National Center for Cell Science (NCCS), Pune, India.
Authentication	The cell lines were not authenticated.
Mycoplasma contamination	HEK293T, 293T-hACE-2, and Vero E6 cell lines tested negative for mycoplasma contamination. Expi293F (Gibco) was not tested for mycoplasma contamination.
Commonly misidentified lines (See ICLAC register)	No commonly misidentified cell lines were used.

Animals and other organisms

Policy information about studies involving animals; ARRIVE guidelines recommended for reporting animal research

Laboratory animals	Syrian golden hamsters (Mesorectums auratus) aged 5-7 weeks of both sexes (80-100 gm of weight) were procured from CPCSEA registered, Biogen Laboratory Animal Facility (Bangalore, India).

Wild animals	No wild animals were used in the study.
Field-collected samples	No field collected samples were used in the study.
Ethics oversight	All animal experiments were performed in Virus BSL-3 laboratory at Centre for Infectious Disease Research, Indian Institute of Science, Bangalore, India, following CPCSEA (The Committee for the Purpose of Control and Supervision of Experiments on Animals) and ARRIVE guidelines. The work plans were reviewed and approved by the Indian Institute of Science, Institute Animal Ethical Committee (IAEC).

Note that full information on the approval of the study protocol must also be provided in the manuscript.
